# Multicomponent adsorption of heavy metals: competitive interaction and critical evaluation of reusability of magnetic activated carbon

**DOI:** 10.1039/d6ra07517g

**Published:** 2026-11-06

**Authors:** Gabriela A. Nogueira, Altair B. Moreira, Odair P. Ferreira, Márcia C. Bisinoti

**Affiliations:** a São Paulo State University (UNESP), Institute of Biosciences, Modern Languages and Exact Sciences (IBILCE), Department of Chemistry and Environmental Sciences (DQCA), Laboratory of Environmental Sciences Studies (LECA) 2265 Cristóvão Colombo St., Jardim Nazareth São José do Rio Preto SP ZIP code 15054-000 Brazil gabriela.aparecida@unesp.br altair.moreira@unesp.br; b State University of Londrina (UEL), Department of Chemistry, Advanced Functional Materials Laboratory (LaMFA) Celso Garcia Cid Highway, PR 445, km 380 Londrina PR ZIP code 86055-900 Brazil opferreira@uel.br

## Abstract

Magnetic activated carbons (MACs) derived from biomass have demonstrated considerable potential as a water treatment solution for the removal of heavy metals. In this study, sugarcane bagasse-derived MACs were produced by hydrothermal carbonization and subsequently thermochemically activated with KOH prior to application. Adsorption experiments were conducted using single- and multicomponent systems. The adsorption capacities were lower for the multicomponent systems due to interactions among heavy metals. Isotherm studies revealed an initial saturation of adsorption sites with Pb(ii), while Cd(ii) and Ni(ii) continued adsorbing at higher concentrations, resulting in adsorption capacities in the order of Ni(ii) > Cd(ii) > Pb(ii). Kinetic studies demonstrated that the pseudo-first order and intraparticle diffusion-chemisorption models best described the adsorption behavior, indicating an adsorption process controlled by multiple mechanisms. The equilibrium data was best fitted by the Sips isotherm model, indicating adsorption on energetically heterogeneous active sites characterized by Freundlich-like behavior at lower concentrations and progressive Langmuir-like saturation at higher metal concentrations. Optimal adsorption capacities were achieved at pH 5.0 with a dosage of 0.50 g L^−1^ and an equilibrium time of 30 min (0.79, 1.06, and 1.45 mmol g^−1^ for Pb(ii), Cd(ii), and Ni(ii), respectively). Thermodynamic parameters confirmed the spontaneous nature of the adsorption, which was exothermic for Pb(ii) and endothermic for Cd(ii) and Ni(ii). The material was recycled for three cycles before beginning to lose its magnetism. These results demonstrate the potential of this magnetic activated carbon as an efficient and sustainable multifunctional adsorbent for heavy metal removal.

## Abstract 2

Optimized magnetic activated carbon (MAC) from sugarcane bagasse effectively removes heavy metals over three cycles. Progressive Fe leaching reduces magnetic stability, emphasizing the need for critical reusability evaluation in water treatment.

## Graphical abstract

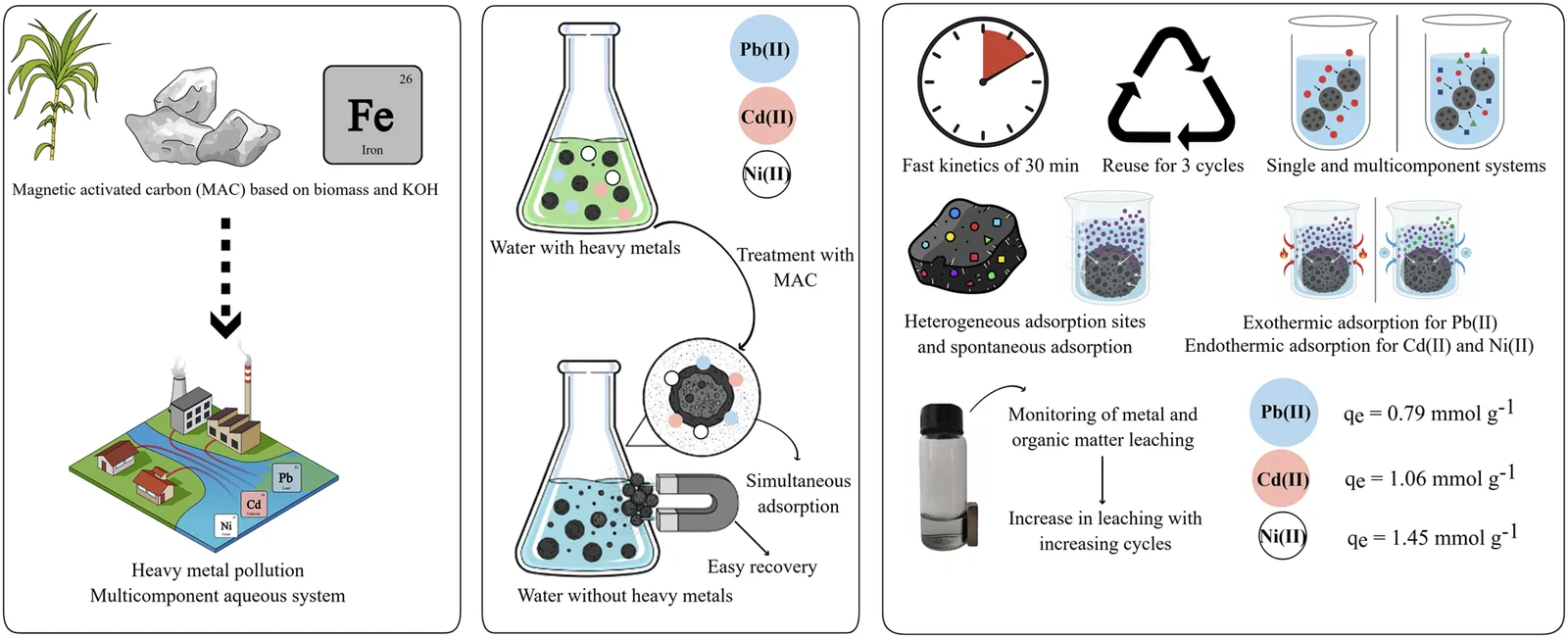

## 1 Introduction

The treatment of contaminated water containing heavy metals remains a critical global concern due to the high persistence and bioaccumulative potential of these contaminants across multiple trophic levels.^1^ Among the most common toxic metals in aqueous effluents are lead (Pb), cadmium (Cd), and nickel (Ni), primarily originating from industrial processes such as paint manufacturing, battery production, electroplating of iron and steel, and tanning.^2–4^ These metals are included in the Agency for Toxic Substances and Disease Registry's Priority List of Hazardous Substances, based on three key factors, which are toxicity, frequency of occurrence, and potential risk to human exposure.^5^

Several processes have been utilized for the purpose of metal removal, including adsorption, filtration, precipitation, coagulation, ion-exchange membranes, and electrodialysis.^6–9^ Among these, adsorption garnered significant scientific interest due to its simplicity, cost-effectiveness, and eco-friendliness. Notwithstanding the challenges, adsorption processes continue to be a subject of significant research interest. These challenges include the need for efficient regeneration, sensitivity to pH and temperature variations, and limited selectivity.^10,11^ Despite these limitations, adsorption is widely recognized in the scientific literature as one of the most effective processes for metal removal, prompting extensive research into the development of advanced adsorbent materials with high specific surface areas and adsorption capacities.^12,13^

The increasing demand for sustainable adsorption is driving the utilization of agro-industrial waste, such as sugarcane bagasse, in the production of reusable magnetic adsorbents, including magnetic carbons (MCs). These absorbents ensure efficient recovery.^14,15^ In our research group, MCs have been prepared by HTC, exhibiting magnetic properties of approximately 4.2 emu g^−1^,^16^ a homogeneous particle distribution, a low specific surface area of ∼9–14 m^2^ g^−1^, and a limited adsorption capacity for hexavalent chromium (3.3–4.6 mg g^−1^).^15^ To enhance the adsorption properties, thermochemical activation with KOH was employed, resulting in a 50-fold increase in specific surface area and a threefold improvement in hexavalent chromium adsorption capacity in magnetic activated carbon (MAC).^15^

A variety of materials have been used in the removal of single components of heavy metals from aqueous solutions.^13,15,17^ However, relatively few studies have investigated multicomponent adsorption systems.^18,19^ Kothavale *et al.*^20^ used carboxyl- and thiol-functionalized magnetic nanoadsorbents for ternary multicomponent to demonstrate the adsorption capacity of a ternary system: 0.31 mmol g^−1^ for Pb(ii), 0.48 mmol g^−1^ for Cd(ii), and 0.46 mmol g^−1^ for Ni(ii). Similarly, zeolites have been used in multicomponent systems containing several metals, exhibiting an adsorption capacity of 0.21 mmol g^−1^ for Cd(ii), 0.27 mmol g^−1^ for Pb(ii), and 0.52 mmol g^−1^ for Cu(ii).^21^ Furthermore, biogenic hydroxyapatites have demonstrated a high affinity for Pb(ii) in a system containing Cd(ii) and Cu(ii), reaching an adsorption capacity of 1.06 mmol g^−1^ under competitive adsorption conditions.^22^

Despite the extensive literature on the development of adsorbents, a critical gap persists between idealized laboratory experiments and the inherent complexity of effluents.^18,19^ The majority of studies prioritize single-component evaluations, failing to capture the competitive interactions typical of real-world wastewater.^18,19^ While our previous work established the material's production, characterization, and performance with anionic species, understanding its behavior toward cationic contaminants in competitive matrices is essential to evaluate its operational versatility.^15^ To address this limitation, the present study evaluates the competitive adsorption behavior of Pb(ii), Cd(ii), and Ni(ii) in multicomponent systems using MACs. Additionally, while the reuse of magnetic adsorbents is well-documented,^23^ many studies neglect to address the critical issues such as secondary pollution and material degradation over consecutive cycles. Specifically, the leaching of total organic carbon (TOC) and iron (Fe) can compromise effluent quality and can lead to a progressive loss of the adsorbent's magnetic recovery efficiency. Rigorously monitoring these frequently overlooked parameters during reuse cycles, this work ensures a realistic approach to water purification and aligns with Sustainable Development Goals 6 and 12 by promoting the safe and circular application of magnetic materials.

## 2 Materials and methods

### 2.1 Chemicals and materials

Information on the sugarcane bagasse used, as well as the purity of the chemicals and gases, can be found in the SI (Section 1.1).

### 2.2 MAC production

Two MCs were prepared using the Nogueira *et al.*^15^ method *via* HTC. The prepare involved keeping 1.8 g of iron(iii) nitrate nonahydrate, 60.0 mL of ultrapure water, and 3.0 g of sugarcane bagasse within in a stainless-steel reactor (PARR, 4598) at 230 °C (MC-230) or 270 °C (MC-270) for 12 h. The MCs were subsequently thermochemically activated using the previously reported procedure,^15^ resulting in four MACs. For this, the MCs were mixed with KOH at a 1 : 2 mass ratio (w/w) in a pretreatment. The resulting solids were transferred to alumina boats and subjected to thermal treatment in a tubular furnace (EDG, FT-HI/20/Bipartite) under a nitrogen (N_2_) flow of 300 mL min^−1^ at 500 and 700 °C for 1 h.

Four control materials (MACs-C) were prepared using the same procedure, but without an activation agent (*i.e.*, KOH). The MACs were labeled MAC-*x-y-z*, where *x* is the prepared temperature (230 °C or 270 °C), *y* is the KOH ratio (2 for a ratio of 1 : 2 or 0 for no KOH), and *z* is the activation temperature (5 for 500 °C or 7 for 700 °C).

### 2.3 Batch adsorption studies of Pb(ii), Cd(ii), and Ni(ii)

In all batch experiments, single- and multicomponent solutions were prepared by diluting a stock solution of lead(ii) nitrate, cadmium(ii) nitrate hexahydrate, and nickel(ii) nitrate tetrahydrate (0.02–100.0 mmol L^−1^). In all multicomponent (ternary) adsorption experiments, the specified initial concentrations strictly refer to the individual concentration of each metal ion [Pb(ii), Cd(ii), and Ni(ii)] present in the mixture. Molar units were deliberately employed instead of mass-based concentrations (mg L^−1^) to ensure an equimolar stoichiometric competition among the metal species. Due to the substantial differences in atomic weights between Pb (207.2 g mol^−1^), Cd (112.4 g mol^−1^), and Ni (58.7 g mol^−1^), evaluating multicomponent systems on a mass basis can misrepresent the true site-binding affinity and artificially overestimate species with higher molar masses. The adsorbents and metal solutions were suspended and subjective to ultrasonication for a duration of 1 min (Ultronique, Q3.0/37A). Thereafter, the samples were agitated at a frequency 180 rpm on an orbital shaker (Solab, SL-180/DT) for a predetermined duration of contact. Subsequently, the solids were separated by vacuum filtration using PVDF membranes (Chromastore, 0.22 µm) prior to analysis by flame atomic absorption spectrometry (FAAS, Section 2.4). All experiments were conducted in triplicate using Milli-Q® ultrapure water (18.2 MΩ cm^−1^).

#### 2.3.1 Evaluation of multi- and single-component adsorption

A preliminary multicomponent batch adsorption experiment was performed with all adsorbents. The tests were conducted for 24 h at a temperature of 21.0 ± 0.4 °C and a pH of 4.5 (due to the possibility of precipitation of all metals, especially Pb(ii)). The tests utilized 15.0 mL of ternary solutions containing Pb(ii), Cd(ii), and Ni(ii) at 0.02, 0.25, or 2.0 mmol L^−1^, with 15.0 mg of each adsorbent (adsorbent dose: 1.0 g L^−1^). Control experiments, consisting of metal solutions without adsorbents, were conducted at the same concentrations (0.02–2.0 mmol L^−1^). The adsorbed amount of each metal (*q*_*t*_, mmol g^−1^) was calculated according to eqn (1).1
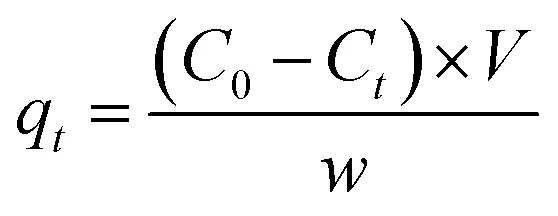
where *V* denotes the volume of the solution (L), *w* represents the mass of the adsorbent (g), and *C*_0_ and *C*_*t*_ are the initial and final concentrations of total Pb, Cd, or Ni at time *t* (mmol L^−1^), respectively.

Furthermore, a single-component adsorption experiment was conducted under the same conditions, employing Pb(ii), Cd(ii), and Ni(ii) solutions at 0.25 mmol L^−1^ to assess the adsorption capacities (*q*_*t*_) in single-metal systems. The adsorbed amounts were also calculated using eqn (1).

#### 2.3.2 Effect of pH

MAC-270-2-7 was selected for multicomponent adsorption experiments with Pb(ii), Cd(ii), and Ni(ii) at 21.0 ± 0.4 °C for 24 h. The initial metal concentration was 0.25 mmol L^−1^, and the pH was adjusted to 2.0, 3.0, 4.0, 5.0, and 6.0. An adsorbent dose of 1.0 g L^−1^ was applied. Control experiments were also conducted, containing only the multicomponent metal solutions at identical concentrations and pH, but without adsorbent. The amount of each metal that was adsorbed (*q*_*t*_, mmol g^−1^) was determined according to eqn (1).

#### 2.3.3 Effect of adsorbent dose

MAC-270-2-7 was subjected to multicomponent adsorption experiments with Pb(ii), Cd(ii), and Ni(ii) under the same conditions described for the pH effect study (Section 2.3.2). The pH was maintained at 5.0, and the adsorbent dose was varied by 0.25, 0.50, 0.75, and 1.00 g L^−1^. Control experiments were also performed, containing only MAC-270-2-7 at the same doses. The adsorbed amount of each metal (*q*_*t*_, mmol g^−1^) was calculated according to eqn (1). Iron leaching and total organic carbon (TOC) release were monitored by FAAS (Section 2.4) and using a TOC analyzer equipment (Shimadzu, TOC-VCSN), respectively, across the different adsorbent doses to verify the stability of the material.

#### 2.3.4 Effect of contact time

The contact time (kinetic) experiment was conducted under the same conditions described in Section 2.3.3, except for the initial metal concentrations, which were set at 0.25, 10.0, and 100.0 mmol L^−1^. The adsorbent dose was set at 0.50 g L^−1^, and the contact times ranged from 5 to 1440 min. Control experiments containing only the multicomponent solutions of Pb(ii), Cd(ii), and Ni(ii) at the same concentrations were likewise subjected to agitation for the same time intervals. The amount of each metal adsorbed at time *t* (*q*_*t*_, mmol g^−1^) was calculated using eqn (1). The nonlinear theoretical kinetic models of Pseudo-First Order (PFO, eqn S1), Pseudo-Second Order (PSO, eqn S2), Intraparticle Diffusion (ID, eqn S3), and Diffusion-Chemisorption (DC, eqn S4) were adjusted to the experimental data.^24,25^ The adjusted coefficient of determination (Adj. *R*^2^, eqn S6), root mean square error (RMSE, eqn S6), and chi-square (*χ*^2^, eqn S7) functions were calculated to validate the model fit.^24,26,27^ Further details are provided in the SI (Section 1.3.1).

#### 2.3.5 Effect of concentration

The adsorption isotherm study was conducted under the conditions described in Section 2.3.4, with the exception of the contact time, which was set at 30 min, and the initial concentrations of the multicomponent solutions of Pb(ii), Cd(ii), and Ni(ii), which ranged from 0.02 to 100.0 mmol L^−1^. To ensure the validity of the findings, control experiments were conducted. The control experiments consisted of multicomponent solutions of the same concentrations. The equilibrium adsorption capacity for each metal (*q*_e_, mmol g^−1^) was calculated using eqn (2).2
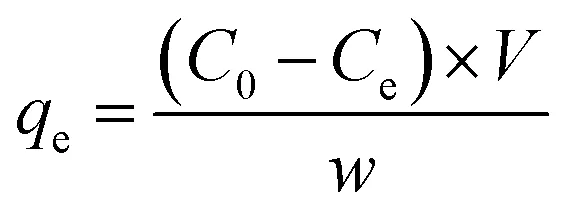
where *V* denotes the volume of the solution used (L), *w* indicates the mass of the adsorbent (g), and *C*_0_ and *C*_e_ are the initial and equilibrium concentrations of total Pb, Cd, or Ni, respectively.

The adsorption isotherm was evaluated using the nonlinear models of the Freundlich (FR, eqn S8), Langmuir (LA, eqn S9), Extended Langmuir (ELA, eqn S10), Sips (SI, eqn S11), Extended Sips (ESI, eqn S12), and Temkin (TE, eqn S13).^25,28–30^ The error functions Adj. *R*^2^, RMSE, and *χ*^2^ were calculated to validate the isotherm model fittings, as defined by eqn S5–S7.^26–28^ An analogous experiment was conducted under optimized adsorption conditions using a buffered solution of 0.5 mol L^−1^ acetic acid and 0.9 mol L^−1^ sodium acetate. Further elucidation can be found in the SI (Section 1.3.2).

#### 2.3.6 Thermodynamic parameters of multicomponent adsorption

An experiment was conducted to evaluate the influence of temperature on the multicomponent adsorption of Pb(ii), Cd(ii), and Ni(ii). The experiment was designed to allow the determination of thermodynamic parameters, including enthalpy (Δ*H*; J mol^−1^), entropy (Δ*S*; J mol^−1^ K^−1^), and Gibbs free energy (Δ*G*; J mol^−1^). The protocol delineated in Section 2.3.5 was meticulously adhered to, with adsorption temperatures set at 21.0 ± 0.4 °C, 29.0 ± 0.4 °C, 34.0 ± 0.8 °C, and 44.0 ± 0.7 °C (corresponding to 294.15 K, 302.15 K, 307.15 K, and 317.15 K, respectively). Further details concerning the calculations can be found in the SI (Section 1.3.3).

#### 2.3.7 Reusability

##### 2.3.7.1 Evaluation of desorption efficiencies with different eluents

To assess the reusability of MAC-270-2-7, a cyclic multicomponent adsorption–desorption experiment was performed. The experimental conditions were consistent with adsorption isotherm procedure delineated in Section 2.3.5, with the initial metal ion concentration fixed at 100.0 mmol L^−1^. A control experiment was conducted using a multicomponent solution of Pb(ii), Cd(ii), and Ni(ii) at the same concentration, but without adsorbent. After achieving equilibrium, the amount of each metal adsorbed (*q*_e_, mmol g^−1^) was determined through the implementation of eqn (2). PVDF membranes containing the material with adsorbed metals were dried at 65 °C to constant weight, and the adsorbent was then removed by scraping. Subsequently, the multicomponent desorption efficiency over 24 h was evaluated using a dose of 0.50 g L^−1^ in the following eluents: 0.5 mol L^−1^ HCl, 0.5 mol L^−1^ CH_3_COOH, 0.5 mol L^−1^ HNO_3_, and ultrapure water (18.2 MΩ cm^−1^).^31^ The controls solutions, which consisted exclusively of the respective eluent solutions, were used under conditions that were identical to those of the experimental solutions.

After desorption, the adsorbent was separated by vacuum filtration, washed with ultrapure water (18.2 MΩ cm^−1^) until a stable pH was achieved, dried at 65 °C to a constant weight, and removed from the membranes by scraping. Iron leaching was monitored by FAAS (Section 2.4), and TOC release was evaluated using a total organic carbon analyzer (Shimadzu, TOC-VCSN). The desorption efficiency (%) percentage was determined throughout the cycles using eqn (3).3
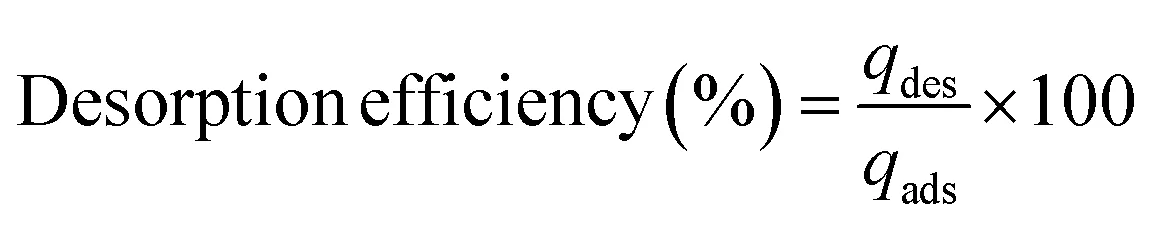
where *q*_ads_ denotes the amount adsorbed (mg), while *q*_des_ signifies the amount desorbed (mg).

##### 2.3.7.2 Cycle adsorption using acetic acid as eluent

Cyclic multicomponent adsorption experiments were conducted using acetic acid as the eluent, adhering to the conditions outlined in Section 2.3.7.1. However, the experiment was modified by employing a 0.5 mol L^−1^ acetic acid solution as the desorbing agent. After the attainment of equilibrium, the amount of each metal adsorbed at equilibrium (*q*_e_, mmol g^−1^) was calculated using eqn (2). PVDF membranes containing the adsorbent with the adsorbed metals were then subjected to drying at 65 °C to constant weight. This was followed by the removal of the material by scraping. The desorption efficiency of Pb(ii), Cd(ii), and Ni(ii) was then evaluated using an acetic acid solution with a concentration of 0.5 mol L^−1^ and an adsorbent dose of 0.50 g L^−1^. After the desorption step, the same procedure described Section 2.3.7.1 was employed.

### 2.4 Quantification of total Pb, Cd, Ni, Fe, and K by FAAS

The concentrations of total Pb, Cd, and Ni, as well as total Fe (Sections 2.4.3 and 2.4.7) and total K (Section 2.3.7), in the residual solutions from the adsorption experiments were determined by FAAS (Varian, AA240FS). Absorbance measurements were carried out at wavelengths of 283.3 nm for total Pb, 228.8 nm for total Cd, 352.5 nm for total Ni, and 248.3 nm for total Fe. The total K quantification was performed in emission mode at 766.5 nm. Analytical calibration curves were constructed by diluting standard solutions (1000.0 mg L^−1^, Fluka™) within the following concentration ranges: 0.0–30.0 mg L^−1^ for total Pb; 0.0–1.8 mg L^−1^ for total Cd; 0.0–20.0 mg L^−1^ for total Ni; 0.0–3.0 mg L^−1^ for total Fe; and 0.0–1.5 mg L^−1^ for total K. Instrumental operating conditions, including gas flow rates and flame parameters, were detailed in Table S1 and followed the manufacturer's specifications. Analytical curves are provided in Fig. S1–S5.

## 3 Results and discussion

### 3.1 Adsorption studies

#### 3.1.1 Evaluation of multi- and single-component adsorption

Preliminary single- and multi-component adsorption screening tests were conducted to evaluate the performance of the produced materials, textural properties of MACs-C and establish optimal operational parameters (detailed in Section S3.1., Fig. S6–S8, and Table S2, SI). Among the evaluated initial concentrations (0.02, 0.25, and 2.00 mmol L^−1^), 0.25 mmol L^−1^ was selected as the optimal working concentration to assess competitive dynamics without complete analyte depletion. Across both single- and multi-component systems, MAC-270-2-7 consistently exhibited the highest uptake capacities for Pb(ii), Cd(ii), and Ni(ii), which is attributed to its high surface area and accessible mesoporous network. Consequently, MAC-270-2-7 was identified as the most efficient adsorbent and, alongside the 0.25 mmol L^−1^ concentration, was selected for all subsequent studies.

#### 3.1.2 Effect of pH

The multicomponent adsorption of Pb(ii), Cd(ii), and Ni(ii) onto MAC-270-2-7 as a function of pH is demonstrated in Fig. 1. As the pH level increased from 2.0 to 6.0, the adsorption capacities for Pb(ii), Cd(ii), and Ni(ii) increased by 61.1-, 5.5-, and 1.6-fold, respectively. This enhancement is mainly attributed to the progressive increase in electrostatic attraction as the adsorbent surface becomes more negatively charged at elevated pH levels.^32,33^ This phenomenon is in full accordance with the point of zero charge (pH_pzc_) as determined in our previous characterization study.^15^ Analogous pH-dependent behavior has been extensively documented for the adsorption of cationic species on magnetic metal–organic frameworks (MOFs)^34^ and other magnetic systems.^17,35^

**Fig. 1:**
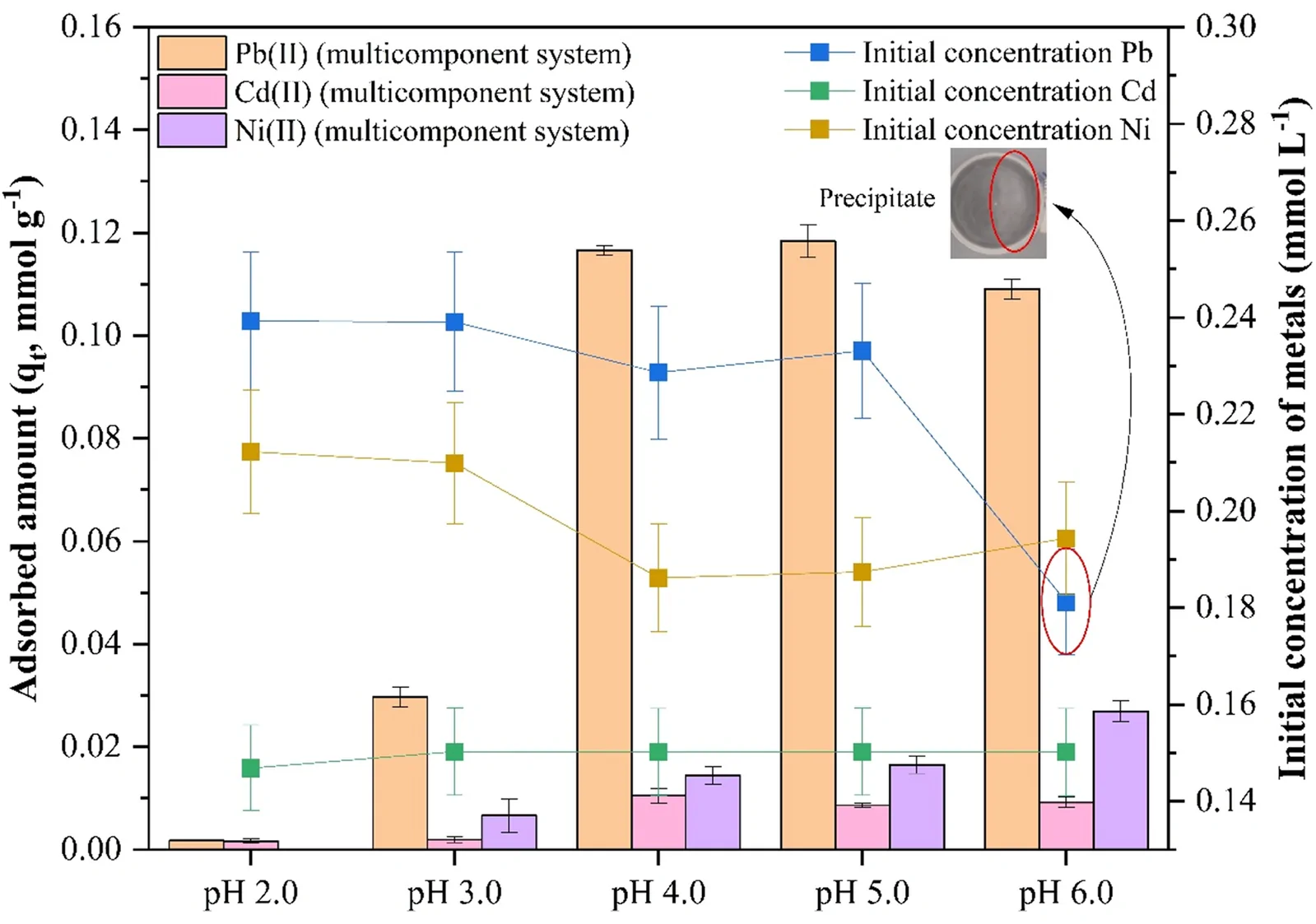
Multicomponent adsorption of Pb(ii), Cd(ii), and Ni(ii) as a pH function (2.0–6.0). Contact time: 24 h, initial concentration of each metal in the multicomponent solution: 0.25 mmol L^−1^, MAC-270-2-7 dose: 1.00 g L^−1^, and temperature: 21.0 ± 0.4 °C. The inset indicates the Pb(OH)_2_ precipitation. The initial pH was adjusted to nominal target values of 2.0, 3.0, 4.0, 5.0, and 6.0 (real measured initial and final pH values are detailed in Table S3).

At a pH of 6.0, partial precipitation of Pb(ii) as Pb(OH)_2_ was observed, as evidenced by a decrease in the initial total Pb concentration (Fig. 1). This finding is consistent with the results reported by Han *et al.*^36^ suggests that further testing at this or higher pH levels is not necessary. With respect to Cd(ii) and Ni(ii), the minor concentration variations observed at pH 3.0 and 4.0 (Fig. 1) are attributable to experimental error, as each solution at a specific pH was prepared separately. This confirms that no precipitation occurred for these elements. Consequently, pH 5.0 was identified as the optimal condition, ensuring the concurrent adsorption of all three metals without the occurrence of precipitation.

The final pH value following adsorption differed from the pH values of the controls containing only the multicomponent Pb(ii), Cd(ii), and Ni(ii) solutions (Table S3). Initial pH values of 2.0, 3.0, and 4.0 exhibited an increase in the final pH, while at pH 5.0 and 6.0 demonstrated a decrease. This behavior is attributed to heterogeneous acid–base reactions involving surface acid functional groups of MAC-270-2-7,^15^ which undergo protonation at low pH, consuming H^+^ and increasing the equilibrium pH, and deprotonation at higher pH, releasing H^+^ and decreasing the final pH, as previously reported.^37^

#### 3.1.3 Effect of adsorbent dose

The optimal MAC-270-2-7 dosage was assessed in multicomponent Pb(ii), Cd(ii), and Ni(ii) adsorption (Fig. 2). Pb(ii) adsorption decreased by 19% when the dosage increased from 0.25 to 1.00 g L^−1^, likely due to particle agglomeration and the consequent overlapping of active sites. This phenomenon is consistent with the morphological characteristics we reported in previous works.^15,38,39^ In contrast, Cd(ii) removal at 0.25 g L^−1^ was 66% lower than at 1.00 g L^−1^, while Ni(ii) was not adsorbed at 0.25 g L^−1^. The removal of all three metals was achieved with varying degrees of efficacy, with Pb(ii) demonstrating the highest degree of adsorption. It is noteworthy that elevating the dosage beyond 0.50 g L^−1^ did not result in any clear improvement within the experimental uncertainty for Cd(ii) and Ni(ii) uptake. Consequently, 0.50 g L^−1^ was identified as the optimal dosage, achieving a balance between maximum removal efficiency and minimal material consumption.

**Fig. 2:**
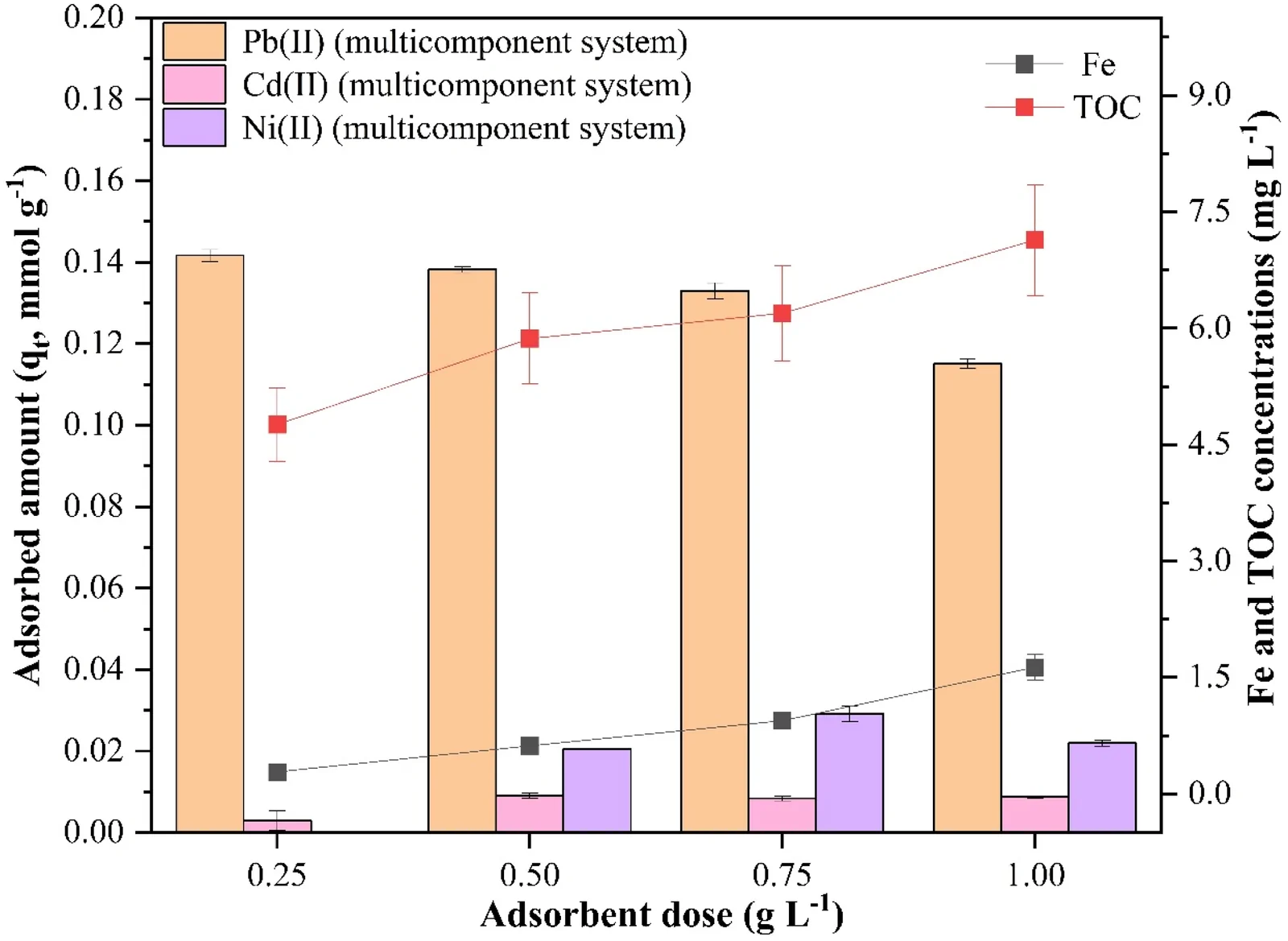
Multicomponent adsorption amount for Pb(ii), Cd(ii), and Ni(ii), and TOC and Fe leaching as a function of the MAC-270-2-7 dose (0.25–1.00 g L^−1^). Initial concentration of each metal in the multicomponent solution: 0.25 mmol L^−1^, contact time: 24 h, initial pH: 5.0, and temperature: 21.0 ± 0.4 °C.

Furthermore, iron (Fe) leaching and Total Organic Carbon (TOC) content were monitored to evaluate the risk of secondary environmental pollution and to assess the structural stability of the magnetic composite. The adsorbent dosage was positively correlated with the Fe and TOC contents, as illustrated in Fig. 2. At a concentration of 0.25 g L^−1^, the leaching levels were recorded at 0.3 mg L^−1^ for Fe (detection limit: 0.030 mg L^−1^) and 4.8 mg L^−1^ for TOC, exhibiting a 5.7- and 1.5-fold increase, respectively, at a concentration of 1.00 g L^−1^.

Despite this increase, all measured Fe concentrations remained well below the maximum limit for industrial wastewater discharge (15.0 mg L^−1^) established by CONAMA Resolution 430/2011.^40^ To provide these values into a broader environmental context, the leached Fe levels at the optimized dosage (0.50 g L^−1^, ∼0.6 mg L^−1^ Fe) are also remarkably low when compared to aesthetic guidelines for drinking water quality, such as the Secondary Drinking Water Standards from the United States Environmental Protection Agency (USEPA, 0.3–1.0 mg L^−1^ for iron).^41^ This further confirms that iron release poses negligible environmental risk.

While TOC is often subject to broad regional environmental regulations rather than universal single-value limits, monitoring the leaching of both inorganic and organic species is a highly relevant issue for carbon-based adsorbents, particularly at high dosages.^42^ Excessive TOC leaching introduces undesired organic loads into the treated effluent, compromising water quality.

Of particular concern is the revelation that continuous Fe leaching not only poses a secondary environmental risk but also progressively degrades the magnetic core of the adsorbent, directly limiting its separation efficiency and practical reusability over multiple cycles. Although the monitoring of these parameters is critical for real-world applications, there is a significant lack of studies in the literature that simultaneously evaluates Fe and TOC leaching in carbonaceous magnetic adsorbents.

The range of TOC contents reported for surface and drinking waters typically falls between 2.0 and 8.7 mg L^−1^.^43,44^ However, lower values are generally preferred to restrict microbial growth and limit the formation of disinfection by-products. These hazardous by-products, including trihalomethanes (THMs) and haloacetic acids (HAAs), are formed when residual organic matter reacts with chemical disinfectants such as chlorine during downstream water treatment processes.^44,45^ Regarding structural stability, Fe leaching from MAC-270-2-7 was minimal (<0.6%) across all tested dosages, indicating highly effective Fe encapsulation within the carbon matrix. In consideration of the simultaneous removal of Pb(ii), Cd(ii), and Ni(ii) while minimizing both TOC and Fe release, a dosage of 0.50 g L^−1^ was determined to be optimal (Fig. 2). While lower dosages can enhance metal-surface interactions and accessibility to active sites,^38,39^ the 0.50 g L^−1^ dosage was found to provide an optimal balance between minimizing material consumption and maximizing the overall adsorption amount for all three metals.

#### 3.1.4 Effect of contact time

The equilibrium time is a critical component in evaluating the practical applicability of an adsorbent in multicomponent systems. Kinetic experiments were conducted at initial concentrations of 0.25, 10.0, and 100.0 mmol L^−1^ (Fig. 3, S9a and b). It is noteworthy that for MAC-270-2-7, the adsorption of Pb(ii), Cd(ii), and Ni(ii) had already attained, or was nearing, its equilibrium plateau by the first measured point of 5 min for all tested concentrations. This extremely rapid removal in the initial minutes indicates that the adsorption phase corresponds to an almost instantaneous mass transfer and high accessibility to surface sites. To ensure complete equilibration, it is necessary to verify the absence of subsequent desorption. In addition, to maintain experimental consistency for subsequent assays, 30 min was selected as the standard contact time.

This ultrafast kinetic performance is particularly noteworthy when contrasted with the findings of earlier studies, which documented considerably longer equilibrium times. The adsorption of Pb(ii) on magnetic alginate carbon and hydroxyapatite occurs within a time frame of 80 to 120 min,^22,46^ while the adsorption of Cd(ii) on activated red mud and functionalized carbon nanotubes takes place over a period of 4 to 120 min.^47,48^ The adsorption of Ni(ii) on functionalized carbon microspheres and modified sulfonated graphene oxide composites occurs within a time frame of 60 to 480 min.^49,50^ The rapid adsorption kinetics observed for these systems can be attributed to the highly accessible mesoporous network of MAC-270-2-7, which demonstrates its exceptional mass transfer efficiency and highlighting its strong potential for practical and rapid multicomponent wastewater treatment.

At an initial concentration of 0.25 mmol L^−1^ (Fig. S9a), Pb(ii) dominated adsorption due to its smaller hydration radius and higher polarizability. This phenomenon is attributed to the enhanced diffusivity and stronger interactions with surface acid groups,^35,51–54^ primarily the phenolic and lactonic groups that had been previously characterized in MAC-270-2-7 by Boehm titration.^15^ With increasing concentrations (from 10 to 100 mmol L^−1^; Fig. 3 and S9b, respectively), Pb(ii) adsorption reached a state of saturation. This limitation can be attributed to the rapid occupation of specific high-energy sites by Pb(ii), which is accompanied by the larger dehydrated ionic radius of Pb(ii) (∼1.19 Å). The resultant steric hindrance, caused by the surface packing effect, restricts the accommodation of additional Pb(ii) ions. In this scenario of Pb(ii) saturation, Cd(ii) and, especially, Ni(ii) have become more competitive.

Notably, Ni(ii) exhibited the highest adsorption capacity at 100 mmol L^−1^ (Fig. 3). At this elevated concentration, Ni(ii) benefits from its diminished dehydrated ionic radius (∼0.69 Å) and augmented charge density, experiencing less steric hindrance on the material's heterogeneous surface. This behavior is consistent with literature reports indicating that oxygen-containing groups enhance Ni(ii) adsorption through a combination of electrostatic interactions and inner-sphere surface complexation,^55,56^ outcompeting both Cd(ii) and Pb(ii), which followed in decreasing order of adsorption capacity. In addition, the smaller ionic size of Ni(ii) enables it to occupy the accessible pore volume more efficiently through additional surface and molar packing interactions. The observed trends suggest that the selectivity of adsorption in multicomponent systems is determined by multiple factors. These factors include the ionic size and hydration energy of the adsorbate, as well as structural accessibility to the pores, steric constraints, the specific nature of the adsorbent surface functionalities and coordination preferences at varying concentration levels.

**Fig. 3:**
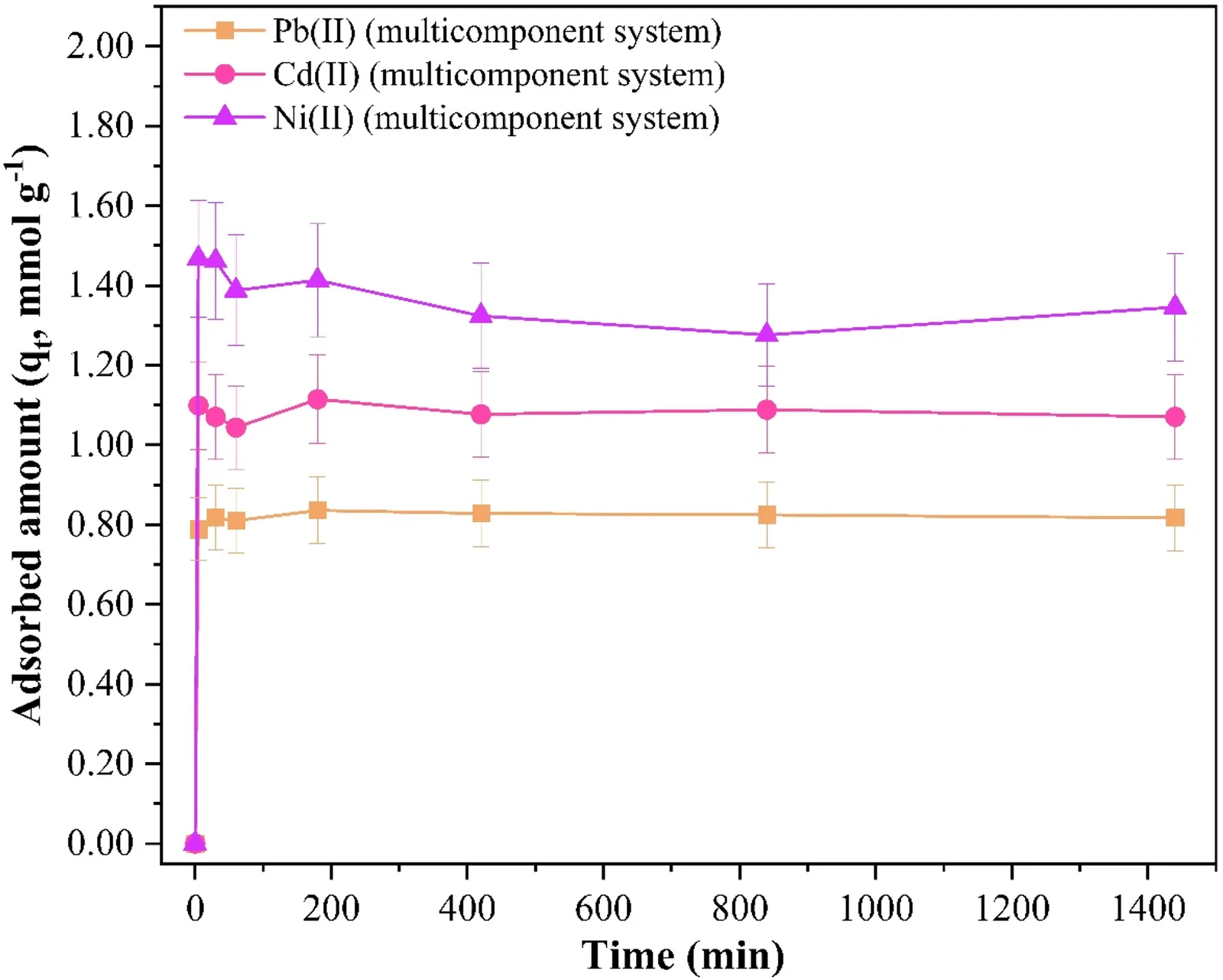
Effect of contact time in multicomponent adsorption of Pb(ii), Cd(ii), and Ni(ii) (5–1440 min). Initial pH: 5.0, MAC-270-2-7 dose: 0.50 g L^−1^, initial concentration of each metal in the multicomponent solution: 100.0 mmol L^−1^. Temperature: 21.0 ± 0.4 °C.

The experimental data were fitted to the pseudo-first order (PFO) and pseudo-second order (PSO) models, along with the intraparticle diffusion (ID) and diffusion-chemisorption (DC) models (Fig. S10–S12). It has been demonstrated that a significant number of adsorption studies are not executed with the employment of appropriate error functions to validate nonlinear models, which consequently results in misinterpretation of the data. Therefore, the performance of the models in this study was rigorously evaluated using the adjusted coefficient of determination (Adj. *R*^2^), root mean square error (RMSE), and chi-square (*χ*^2^). The fitted parameters are summarized in Table S4.

As the initial concentration increased from 0.25 to 100.0 mmol L^−1^, the Adj. *R*^2^ values of the non-linear fits approached 1.0, yielding smoother kinetic curves and lower overall error functions (Table S4). Even though all three concentrations were maintained to provide a comprehensive evaluation of kinetic behavior under varying competitive pressures, the 100.0 mmol L^−1^ system was selected as the primary focus for the mechanistic evaluation due to its superior mathematical convergence. For all metals, the PFO, PSO, and DC models exhibited analogous calculated adsorption capacity in the equilibrium (*q*_e_) and Adj. *R*^2^ values. However, the PFO and DC models yielded lower RMSE and *χ*^2^ values, indicating a more precise alignment between the calculated and experimental *q*_e_ (Table S4).

The PFO model delineates adsorption rates that are governed by the availability of active surface sites, a behavior that is characteristic of physisorption on heterogeneous materials.^24,53,57^ In contrast, the PSO model posits that the rate is contingent on the square of unoccupied sites, a phenomenon that is characteristic of chemisorption and that generally predominates at later stages of the process.^24,53,57^ Furthermore, the ID model attributes the rate-limiting step strictly to intraparticle diffusion, whereas the DC model integrates diffusion and chemisorption effects by incorporating external film diffusion with intraparticle transport to the reactive sites.^24,53,57^

The superior fit of the PFO and DC models (Table S4) is highly consistent with the heterogeneous nature of sugarcane bagasse-derived carbonaceous materials, which exhibit intrinsic structural complexity, as detailed in our previous characterization work.^15,58–60^ In contrast, the PSO model typically better describes highly homogeneous systems. These findings suggest the coexistence of PFO- and DC-related mechanisms in this multicomponent system. In essence, the simultaneous adsorption of Pb(ii), Cd(ii), and Ni(ii) onto MAC-270-2-7 is consistent with a combination of physisorption, intraparticle diffusion, and chemisorption. This process is further characterized by electrostatic interactions and inner-sphere surface complexation with the oxygenated functional groups (phenolic, and lactonic) present on the material surface.^15^

#### 3.1.5 Effect of concentration

The equilibrium concentration and adsorption capacity of MAC-270-2-7 were determined through multicomponent adsorption isotherms of Pb(ii), Cd(ii), and Ni(ii) (Fig. 4). At a concentration of 100.0 mmol L^−1^, a trend toward complete saturation of active adsorption sites was observed, with the highest adsorption capacity recorded for Ni(ii) (1.45 mmol g^−1^), followed by Cd(ii) (1.06 mmol g^−1^) and Pb(ii) (0.79 mmol g^−1^). To ensure full site saturation, experiments at concentrations above 100.0 mmol L^−1^ were attempted. However, metal precipitation occurred at higher concentrations (see inset in Fig. 4). Consequently, the experimental framework was conducted up to a concentration of 100.0 mmol L^−1^.

**Fig. 4:**
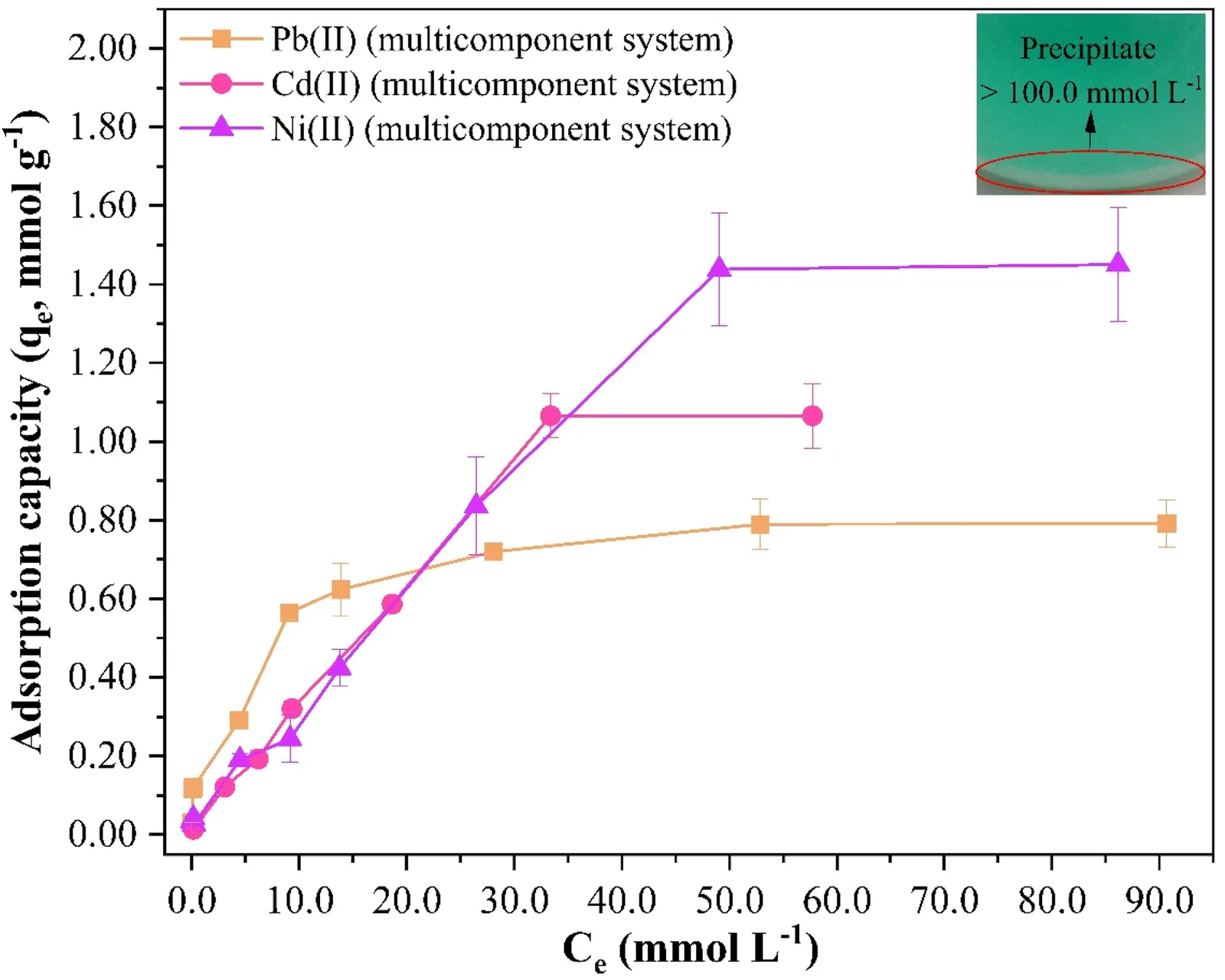
Multicomponent adsorption isotherm for Pb(ii), Cd(ii), and Ni(ii) (concentration of each metal in the multicomponent solution: 0.02–100.0 mmol L^−1^). MAC-270-2-7 dosage: 0.50 g L^−1^, initial pH: 5.0, contact time: 30 min, and temperature: 21.0 ± 0.4 °C. The inset shows that metal precipitation occurs at higher concentrations.

The experimental data were fitted a variety of classical isotherm models, including Freundlich (FR) and Langmuir (LA), as well as Extended Langmuir (ELA), Sips (SI), Extended Sips (ESI), and Temkin (TE) models (Fig. S13a–c). The performance of the model was evaluated using Adj. *R*^2^, RMSE, and *χ*^2^, with the obtained parameters summarized in Table S5.

The LA model posits that adsorption occurs on homogeneous, energetically equivalent sites, thereby forming a monolayer without adsorbate–adsorbate interactions. Equilibrium is achieved when the rates of adsorption and desorption rates are equal.^25,27,28^ The ELA model introduces the concept of competitive adsorption among multiple species.^25,30^ Conversely, the FR model postulates assumes adsorption on heterogeneous surfaces with non-uniform energy distribution and multilayer formation, disregarding surface saturation. It is only valid only when lateral interactions are negligible.^25,27,28^ The SI model amalgamates LA and FR characteristics, reflecting site energy heterogeneity. It exhibits behavior reminiscent of FR at low and that of LA at high concentrations, under the assumption of a finite adsorption capacity, rendering it suitable for heterogeneous systems.^25,27,28^ The ESI model further incorporates competitive adsorption among multiple species.^25,30^ On the other hand, the TE model delineates adsorbent–adsorbate interactions with a linear decrease in adsorption heat. In highly heterogeneous or complex systems, surface coverage is limited.^25,27,28^

Based on the parameters in Table S5, the Sips (SI) model best described the multicomponent adsorption of Pb(ii), Cd(ii), and Ni(ii) by MAC-270-2-7. At low concentrations, adsorption occurred preferentially on high-energy heterogeneous sites, leading to apparent multilayer behavior. At higher concentrations, progressive site occupation resulted in surface saturation and a finite adsorption capacity. This transition in the adsorption mechanism elucidates the observed change in the selectivity among the metals. The *n*_s_ value for Pb(ii) was <1.0, indicating a high degree of affinity at low concentrations, a phenomenon typical of surfaces with selective high-energy sites, such as phenolic hydroxyl groups. However, as the concentration increases, the rapid saturation of these specific sites, combined with the larger ionic radius of Pb(ii), limits the further retention of this cation due to steric hindrance.

For Cd(ii) and Ni(ii), an *n*_s_ > 1.0 suggested a highly heterogeneous surface with sites of varying energies,^27,28,61^ where a cooperative adsorption process occurred. This finding indicates that, while Pb(ii) is constrained by the saturation capacity of high-energy sites, Ni(ii) and Cd(ii), which benefit from their smaller ionic size and higher charge density, can access secondary sites and porous volumes at elevated equilibrium concentrations. Consequently, the Sips model quantitatively corroborates the reversal in the order of maximum adsorption capacity from Pb(ii) > Cd(ii) > Ni(ii) at low concentrations to Ni(ii) > Cd(ii) > Pb(ii) at high concentrations.

To definitively confirm that the high removal efficiency observed at the optimal pH of 5.0 was driven by actual adsorption rather than metal precipitation, an additional experiment was conducted under strictly buffered conditions at pH 5.0. Under these optimized buffered conditions, the adsorption capacities were 0.82, 1.03, and 1.46 mmol g^−1^ for Pb(ii), Cd(ii), and Ni(ii), respectively. A comparison of these values with those of the unbuffered system revealed no substantial differences. This negligible buffering effect confirms that the progressive metal removal is genuinely attributed to interactions with the active surface sites of the material, successfully ruling out precipitation as a false positive. Finally, a comparison with literature data (Table 1) demonstrates that MAC-270-2-7 exhibits similar or superior adsorption capacities, underscoring its substantial potential as a highly effective and versatile adsorbent for complex aqueous matrices. It is imperative to acknowledge that Table 1 is designed to establish a contextual benchmark rather than to offer a direct performance ranking. This is due to the fact that adsorption capacities across diverse studies are strongly influenced by varying experimental parameters, including pH, adsorbent dosage, initial concentration ranges, and competing ions.

**Table 1:** Comparison of the adsorption results obtained by the optimal material in this study (MAC-270-2-7) with other materials presented in the literature

Materials	Coexisting metals	Target metal	*q*_e_ (mmol g^−1^)	Reference
**MAC-270-2-7**	Pb(ii), Cd(ii) e Ni(ii)	Pb(ii)	0.79	This study
		Cd(ii)	1.06	
		Ni(ii)	1.45	
Coal ash	Pb(ii), Cd(ii) e Mg(ii)	Pb(ii)	0.04	62
Zeolite	Cu(ii), Zn(ii), Cd(ii) e Pb(ii)	Cd(ii)	0.21	21
		Cu(ii)	0.52	
		Pb(ii)	0.27	
Biosorbent based on immobilized extracellular polymer	Cr(vi), Cd(ii) e Pb(ii)	Cr(vi)	0.77	63
		Cd(ii)	0.32	
		Pb(ii)	0.21	
Biogenic hydroxyapatite	Pb(ii), Cd(ii) e Cu(ii)	Pb(ii)	1.06	22
		Cd(ii)	0.26	
		Cu(ii)	0.76	
Chitosan-based adsorbent modified with triethylenetetramine	Pb(ii), Cd(ii) e Ni(ii)	Pb(ii)	1.51	50
		Cd(ii)	0.75	
		Ni(ii)	1.46	
Magnetic nanoparticles (Fe_3_O_4_)	Pb(ii), Cd(ii) e Ni(ii)	Ni(ii)	0.46	20
		Pb(ii)	0.31	
		Cd(ii)	0.48	

#### 3.1.6 Thermodynamic parameters of multicomponent adsorption

The multicomponent adsorption capacities of Pb(ii), Cd(ii), and Ni(ii) increased by 69.7%, 15.1%, and 43.1%, respectively, as the temperature increased from 21 to 44 °C (Fig. S14a–c). These results were obtained using a fixed contact time of 30 min, which was determined in Section 3.1.4 to be sufficient to reach equilibrium. At a temperature of 44 °C, the maximum adsorption capacities were 2.63, 1.26, and 2.55 mmol g^−1^ for Pb(ii), Cd(ii), and Ni(ii), respectively. These results confirm the high efficiency of MAC-270-2-7 and its preferential affinity toward Pb(ii) and Ni(ii).

It is well established that elevated temperatures frequently exert a significant influence on the behavior ions within solution.^64^ However, the ultrafast kinetic equilibrium reached in 5 min indicates that diffusion limitations can be considered negligible. Instead, the enhanced adsorption capacity at elevated temperatures is attributable to the thermal energy providing the activation energy required for the partial dehydration of Pb(ii). It is noteworthy that, despite its larger bare ionic radius of 1.19 Å, Pb(ii) exhibits the smallest hydrated radius of 4.01 Å among the three metals. Owing to its lower charge density (*z*/*r*) and lower hydration energy, this desolvation process significantly reduces its effective size and facilitates access to high-affinity active sites, promoting stronger electrostatic interactions and complexation with the surface acid groups,^52–54^ such as phenolic and lactonic groups, which were previously identified on the adsorbent surface.^15^ Ni(ii) adsorption exhibited a significant increase, while Cd(ii) adsorption remained minimal, suggesting that Cd(ii) possesses a lower affinity for these specific sites even under higher thermal energy.

The data were also fitted to the isothermal models FR, LA, ELA, SI, ESI, and TE, as demonstrated in Fig. S15–S17. The thermodynamic parameters (Δ*H*°, Δ*S*°, and Δ*G*°) were determined from the Van't Hoff plots (Fig. S18) as described in Section 1.3.3 of the SI. It should be emphasized that because the ionic strength in the concentrated ternary system (*I* = 0.9 mol L^−1^) exceeds the strict quantitative validity threshold of the Debye–Hückel model (*I* < 0.1 mol L^−1^), the derived thermodynamic values represent model-dependent estimates rather than absolute thermodynamic parameters. Nonetheless, the comparative temperature trends and mechanistic conclusions remain fully valid. Additionally, the linear Van't Hoff fits exhibited *R*^2^ values ranging from 0.90 to 0.93 (Fig. S18), reflecting the inherent uncertainty and complexity of competitive adsorption on energetically heterogeneous surface sites.

The isothermal adsorption parameters and the temperature-dependent error functions are presented in Table S6. The Sips (SI) model demonstrated the optimal fit for the experimental data across all temperatures, exhibiting the most favorable outcomes in terms of Adj. *R*^2^, RMSE, and *χ*^2^. The heterogeneity parameter (*n*_s_) derived from this model, in conjunction with the calculated enthalpy changes, indicates a mixed physisorption–chemisorption mechanism. As previously established in the kinetic evaluation (Section 3.1.4), chemisorption in this context refers specifically to inner-sphere surface complexation between the metal ions and the oxygenated functional groups.

The negative values of the Δ*G*° for all three metals between 21 °C and 44 °C confirmed that the adsorption process was a spontaneous process (Table 2). It is noteworthy that while Pb(ii) adsorption became thermodynamically less spontaneous (*i.e.*, Δ*G*° became less negative) with increasing temperature, its actual adsorption capacity increased. This behavior in the multicomponent mixture is attributed to thermal effects and competitive site access. The application of thermal energy has been demonstrated to facilitate the partial dehydration of Pb(ii), which possesses the largest hydrated radius, 4.01 Å. This process enhances surface conformational flexibility, enabling Pb(ii) to effectively displace competing species and access high-affinity sites that were sterically constrained at lower temperatures. This physical phenomenon elucidates the observation that the apparent maximum adsorption capacity (*q*_ms_) escalates despite a decline in the Sips affinity constant (*K*_s_) with an increase in the temperature. This outcome corroborates the hypothesis that the negative Δ*H*° is indicative of the exothermic nature of the specific site binding, while the enhancement in *q*_ms_ is attributable to and augmented site accessibility. It is imperative to acknowledge that the thermodynamic parameters documented in this study are apparent values derived from isotherm fitting. Subsequent investigations employing an isosteric enthalpy approach across constant coverage levels will provide further insights into the energetic distribution of these heterogeneous surfaces.

**Table 2:** Thermodynamic parameters for Pb(ii), Cd(ii), and Ni(ii) adsorption

Ion	*T* (°C)	Δ*G*^0^ (kJ mol^−1^)	Δ*H*^0^ (kJ mol^−1^)	Δ*S*^0^ (kJ mol^−1^ K^−1^)
Pb(ii)	21.0	−18.96	−88.40	−0.24
	29.0	−16.83		
	34.0	−15.64		
	44.0	−13.28		
Cd(ii)	21.0	−9.11	49.56	0.20
	29.0	−10.91		
	34.0	−11.91		
	44.0	−13.91		
Ni(ii)	21.0	−5.31	63.29	0.23
	29.0	−7.42		
	34.0	−8.59		
	44.0	−10.92		

The calculated Δ*H*° was strongly negative for Pb(ii) (−88.40 kJ mol^−1^), indicating an exothermic process dominated by inner-sphere surface complexation with the oxygenated functional groups (phenolic and lactonic) previously identified on the surface.^15^ In contrast, Cd(ii) and Ni(ii) adsorption were thermodynamically favored at higher temperatures, becoming more spontaneous, which is consistent with literature reports.^64–66^ Overall, these opposing trends demonstrate that the magnitude of thermodynamic spontaneity (Δ*G*°) does not strictly dictate or directly correlate with the final adsorption capacity of the studied metals in this complex, heterogeneous multicomponent system.

The preponderance of the SI model suggests that adsorption occurs through a mixed physisorption–chemisorption mechanism on energetically heterogeneous sites. Specifically, the negative Δ*H*° for Pb(ii) (−88.40 kJ mol^−1^) confirms an exothermic process governed by strong specific interactions with surface functional groups, consistent with inner-sphere complexation. Conversely, Cd(ii) and Ni(ii) exhibited positive Δ*H*° values (49.56 and 63.29 kJ mol^−1^, respectively). Rather than indicating an intrinsically endothermic binding step, this net positive enthalpy change reflects the coupled energy demand required for ion desolvation and the displacement of water molecules co-adsorbed at the interface, which exceeds the heat released during metal-surface binding. It is important to note that these thermodynamic differences do not imply completely different adsorption pathways. Rather, they reflect variations in the relative contributions of physical and chemical driving forces, which shift depending on the specific metal affinity and system concentration.^61,64–66^

The entropy change (Δ*S*°) was found to be negative for Pb(ii) (−0.24 kJ mol^−1^ K^−1^), indicating the formation of a more ordered complex at the solid–liquid interface upon strong inner-sphere binding. In contrast, Δ*S*° for Cd(ii) and Ni(ii) was positive, with values of 0.20 and 0.23 kJ mol^−1^ K^−1^, respectively. As demonstrated in previous studies, heavy metals manifest as highly hydrated ions within aqueous solutions. This positive entropy change is primarily driven by the desolvation process, whereby Cd(ii) and Ni(ii) ions must shed their structured hydration shells to bind to the adsorbent surface. The release of these previously bound water molecules into the bulk solution creates a net increase in system randomness that overcomes the loss of entropy associated with the metal attachment to the surface.^64,65,67^ Overall, Pb(ii) adsorption is largely chemisorption-driven, while Cd(ii) and Ni(ii) adsorption is predominantly driven by entropy and physisorption, although contributions from both mechanisms coexist for all three metals.

Comparable thermodynamic behaviors have been documented in extant literature. For instance, Wu *et al.*^68^ observed spontaneous, endothermic adsorption of Cu(ii), Pb(ii), and Zn(ii) using sugarcane-based modified activated carbon with nitric acid, reporting negative Δ*G*° and positive Δ*H*° values that align closely with the Cd(ii) and Ni(ii) results of the current study. Furthermore, Hikmat *et al.*^69^ investigated Pb(ii) adsorption onto the petiole and fiber of a palm tree, documenting an exothermic process with a range of −17.34 to −12.25 kJ mol^−1^ for Δ*H*° and from −0.08 to −0.07 kJ mol^−1^ K^−1^ for Δ*S*°. In their study,^69^ adsorption was found to be more favorable at lower temperatures, a phenomenon behavior they attributed directly to specific structural changes on that adsorbent surface induced by the Pb(ii) binding.

#### 3.1.7 Reusability

##### 3.1.7.1 Evaluation of desorption efficiencies with different eluents

Desorption experiments were conducted using 0.5 mol L^−1^ HCl, HNO_3_, CH_3_COOH, and H_2_O as eluents (Fig. 5). For Pb(ii), desorption efficiency increased with eluent acidity (HCl > HNO_3_ > CH_3_COOH > H_2_O). The result can be attributed to the substance's high polarizability and strong sensitivity to protonation of surface functional groups, enabling metal release.^70^ However, despite their higher desorption efficiency, strong inorganic acids caused severe deterioration of the magnetic phase (see Fe contents in Fig. 5).

**Fig. 5:**
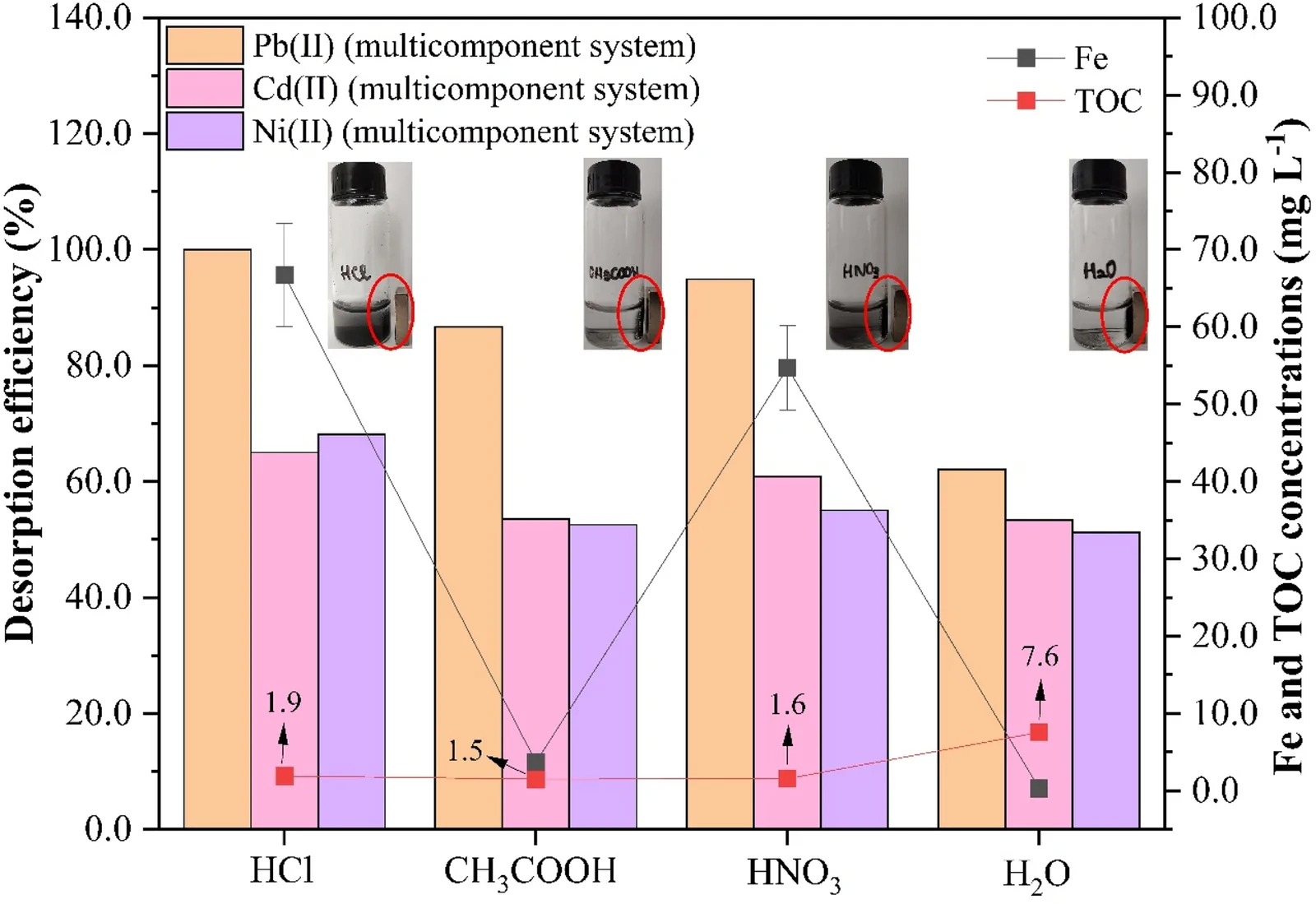
Multicomponent desorption efficiency for Pb(ii), Cd(ii), and Ni(ii) using different eluents (HCl 0.5 mol L^−1^, CH_3_COOH 0.5 mol L^−1^, HNO_3_ 0.5 mol L^−1^, and H_2_O), as well as the leaching of Fe and TOC. The insets indicate attraction to a magnet after the regeneration and elution cycle with the different acids.

The relative iron loss was calculated by using the initial iron mass content, as determined by FAAS in our previous characterization study,^15^ along with the leaching data obtained in this study (Fig. 5). It was found that only 2.9% of the total iron mass in the sample was leached during desorption with 0.5 mol L^−1^ CH_3_COOH, whereas HCl and HNO_3_ promoted substantial Fe losses (52.4% and 27.3%, respectively). This severe iron leaching resulted in a marked decrease in the magnetic response of the material (Table 3 and inset Fig. 5). In addition, CH_3_COOH resulted in a minimal TOC leaching level of 1.5 mg L^−1^. Although this value represents only a slight downward trend compared to the other eluents and falls near the margin of experimental error, it confirms that mild acid effectively prevents the degradation of the carbonaceous matrix. Consequently, while less aggressive acids may slightly decrease overall metal desorption, the 0.5 mol L^−1^ CH_3_COOH solution offers an optimal compromise between effective multicomponent desorption and the critical preservation of structural integrity and magnetism, thereby fully justifying its selection for the subsequent regeneration cycles.

**Table 3:** Percentages of Fe leaching in the preliminary desorption experiment with different eluents

Preliminary cycle	
Eluent	Fe(%)
HCl	52.4
CH_3_COOH	2.9
HNO_3_	27.3
H_2_O	0.2

##### 3.1.7.2 Cycle adsorption–desorption using aqueous solution of acetic acid as eluent

Using 0.5 mol L^−1^ CH_3_COOH as the eluent, the adsorption–desorption cycles exhibited a progressive decrease in multicomponent adsorption capacity from the first to the third cycle. This decline was accompanied by increasing levels of Fe, K, and TOC leaching in each subsequent cycle (Fig. 6a and b). Specifically, the material exhibited structural degradation and a substantial decrease in the total iron content of the material, which dropped from 27.9% to 15.1% after three cycles. This resulted in a severely reduced magnetic response (see inset in Fig. 6b). This physical limitation strictly restricts the material's practical reusability up to three cycles. While many studies have reported that magnetic adsorbents can be reused for five to eight cycles,^71–73^ most of these studies have not monitored secondary metal or TOC leaching. This significant gap in the literature can lead to an overestimation of the actual reuse potential of magnetic composites. This is because a magnetic composite becomes practically unviable for large-scale separation once it loses its magnetic core, even if active surface sites remain available.

**Fig. 6:**
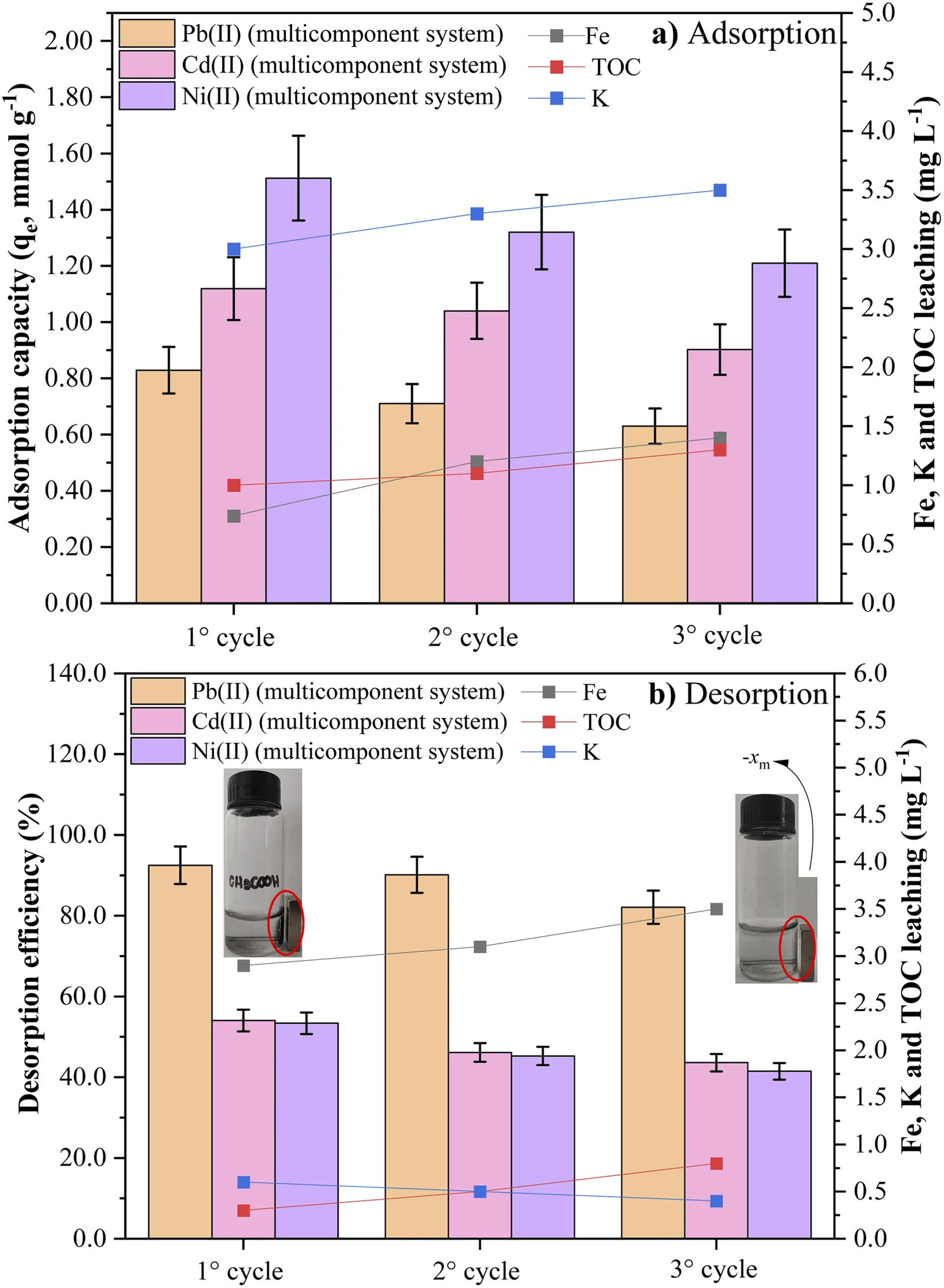
Multicomponent (a) adsorption and (b) desorption for Pb(ii), Cd(ii), and Ni(ii) as a function of the cycle numbers. MAC-270-2-7 dose: 0.50 g L^−1^, initial pH: 5.0, contact time: 30 min, initial concentration of each metal in the multicomponent solution: 100.0 mmol L^−1^, and temperature: 21.0 ± 0.4 °C. Insets in (b): photographs of the desorption vials showing the rapid magnetic separation of MAC-270-2-7 using a low adsorbent dosage, where the small mass of solid adsorbent is aggregated on the inner vial wall adjacent to the external magnet [red circle], yielding a clear supernatant solution. The -*χ*_m_ indicates magnetic loss.

Furthermore, potassium (K) leaching was monitored during these experiments because the MAC-270-2-7 composite was chemically activated with KOH. As previously indicated in our structural characterization study,^15^ this activation process results in the formation of an iron(iii) hexacyanoferrate (Fe_4_[Fe(CN)_6_]_3_) phase. This structure is precipitated during the acid washing of the material activated at 700 °C through the reaction between Fe^3+^ ions and [Fe(CN)_6_]^3−^ species generated during the activation step.^15^ Over the course of three cycles, 62.4% of the initial K was released, predominantly during the adsorption phase. This behavior suggests potential ion exchange mechanisms contributing to the removal of Cd(ii) and Ni(ii), although the simple mobilization and washing of this residual K-containing inorganic phase from the carbonaceous matrix cannot be fully ruled out. Additionally, the molar amount of leached K was inadequate to account for the total moles of adsorbed metals. This discrepancy confirms that ion exchange is not the sole adsorption pathway and that other simultaneous mechanisms, such as cation–pi interactions and the previously discussed inner-sphere surface complexation, are actively involved in this complex multicomponent system.

#### 3.1.8 Adsorption interactions

The multicomponent adsorption of Pb(ii), Cd(ii), and Ni(ii) on the heterogeneous surface exhibited mixed and competitive behavior, reflecting the coexistence of multiple interaction mechanisms. Pb(ii) exhibited a preferential affinity for the adsorbent, predominantly attributable to robust specific interactions, namely, inner-sphere surface complexation with oxygen-containing functional groups. This phenomenon resulted in saturation at lower concentrations when compared to Cd(ii) and Ni(ii). Conversely, the adsorption of Cd(ii) and Ni(ii) appears to be predominantly nonspecific physical interactions. These interactions primarily consist of electrostatic attraction between the metal cations and the negatively charged adsorbent surface, as well as cation–pi interactions with the carbon basal planes. While the leaching of K^+^ during the adsorption process suggests the possibility of minor ion exchange, the distinct lack of stoichiometric correspondence with the adsorbed metal ions, combined with the mobilization of the residual K-containing inorganic phase, indicates that ion exchange is not a primary pathway. The simultaneous removal of these metals suggests contributions from a complex interplay of physical and chemical mechanisms inherently associated with the structural heterogeneity of carbonaceous materials in realistic multicomponent systems.

## 4 Conclusion

The production of magnetic activated carbons (MACs) from sugarcane bagasse has been demonstrated to be a highly effective strategy for the simultaneous remediation of heavy metal-contaminated water. The optimized material (MAC-270-2-7) exhibited superior performance, attributable to its heterogeneous porous structure and a high density of active sites, which were tailored for competitive environments.

A distinguishing feature of this study was the rigorous evaluation of adsorption under multicomponent conditions, which revealed a concentration-dependent selectivity hierarchy. Pb(ii) exhibited preferential adsorption at lower concentrations, while Ni(ii) and Cd(ii) demonstrated distinct competitive affinities across the studied range. This competitive behavior was accurately captured by the nonlinear regression analysis of the isothermal and kinetic models, where the strict application of statistical error functions ensured the reliability of the fitted parameters. Specifically, the high correlation with the Sips isotherm model aligns with adsorption on energetically heterogeneous active sites undergoing progressive saturation, while the kinetic modeling reflects a combined contribution of physical and chemical interactions.

Furthermore, this research employed systematic monitoring of magnetic adsorbents by analyzing total organic carbon (TOC) and iron leaching. The findings of this study demonstrate that high adsorption capacity must be coupled with material stability to prevent secondary pollution, a factor that has been often overlooked in the extant literature. While the MAC exhibited effective magnetic recovery, the observed Fe leaching and the decrease in adsorption capacity across three regeneration cycles underscore the critical need for long-term stability assessments.

In summary, this work provides a comprehensive, self-contained evaluation of the material's performance and operational boundaries in cationic multi-component systems. The bench-scale insights gained regarding competitive interference, ion selectivity, and structural leaching establish the fundamental baseline required to guide practical implementation in complex real-world wastewater matrices. Moreover, the metal recovery potential and the observed leaching post-regeneration suggest that these materials could be repurposed for heterogeneous catalytic applications, aligning with the principles of the circular economy and sustainable water management.

## Author contributions

Gabriela A. Nogueira: writing – original draft, review & editing; methodology; investigation; conceptualization; data curation. Altair B. Moreira: resources; methodology. Odair P. Ferreira: writing – review & editing; methodology; investigation; conceptualization; data curation. Márcia C. Bisinoti: writing – review & editing; resources; supervision; investigation; conceptualization; data curation.

## Conflicts of interest

The authors declare that they have no known competing financial interests or personal relationships that could have appeared to influence the work reported in this paper.

## Supplementary Material

RA-OLF-D6RA07517G-s001

## Data Availability

The raw data is available at https://hdl.handle.net/11449/316663. Supplementary information (SI): reagent purity; adsorption procedures and equations; instrumental conditions for metal determination; nonlinear fitting parameters for kinetic and isotherm models; analytical calibration curves for metals; N_2_ adsorption–desorption isotherms for MACs-C; adsorbed amounts in preliminary single- and multicomponent experiments at different concentrations; multicomponent adsorption kinetics at different concentrations; multicomponent isotherms at different temperatures; linear fittings for thermodynamic parameter determination. See DOI: https://doi.org/10.1039/d6ra07517g.
