# Supplementary material for: Multicomponent adsorption of heavy metals: competitive interaction and critical evaluation of reusability of magnetic activated carbon
Source: RSC Adv. 2026 Nov 6. Online ahead of print. doi: 10.1039/d6ra07517g (PMC13741478; doi:10.1039/d6ra07517g)
Supplement: Supplementary file 1 [file RA-OLF-D6RA07517G-s001.pdf]

Supplementary Information (SI) for RSC Advances.
This journal is © The Royal Society of Chemistry



# SUPPORTING INFORMATION

# Multicomponent adsorption of heavy metals: Competitive interaction and critical evaluation of reusability of magnetic activated carbon

Gabriela A. Nogueira[a], Altair B. Moreira[a], Odair P. Ferreira[b], Márcia C. Bisinoti[a,*]

[a] *São Paulo State University (UNESP), Institute of Biosciences, Modern Languages and Exact Sciences (IBILCE), Department of Chemistry and Environmental Sciences (DQCA), Laboratory of Environmental Sciences Studies (LECA), 2265 Cristóvão Colombo St., Jardim Nazareth, São José do Rio Preto – SP, ZIP code 15054-000, Brazil.* gabriela.aparecida@unesp.br (G.A.N.); altair.moreira@unesp.br (A.B.M.).

[b] *State University of Londrina (UEL), Department of Chemistry, Advanced Functional Materials Laboratory (LaMFA), Celso Garcia Cid Highway, PR 445, km 380, Londrina – PR, ZIP code 86055-900, Brazil.* opferreira@uel.br (O.P.F.).

[*]*Corresponding author: marcia.bisinoti@unesp.br.*

# 1. Materials and methods

## *1.1. Chemicals and materials*

Sugarcane bagasse was supplied by a sugar and ethanol industry located in São Paulo State, Brazil. The sugarcane bagasse was washed with distilled water, oven-dried at 100 °C for 12 h (Tecnik's, NL80/10CEL), ground using a knife mill (Marconi, MA 340), homogenized (Vitalex, LI-02), sieved (Bertel, 0.25 mm), and stored at room temperature. Ferric nitrate nonahydrate ($Fe(NO_3)_3·9H_2O$, 98.0%), potassium hydroxide (KOH, 85.0%), hydrochloric acid (HCl, 37.0%), sodium hydroxide (NaOH, 99.0%), sodium acetate ($CH_3COONa$, 99.0%), acetic acid ($CH_3COOH$, 99.0%), and nitric acid ($HNO_3$, 37.0%) were purchased from Synth (Diadema, SP, Brazil). Lead(II) nitrate ($Pb(NO_3)_2$, 99.0%), cadmium(II) nitrate hexahydrate ($Cd(NO_3)_2·6H_2O$, 98.0%), and nickel(II) nitrate tetrahydrate ($Ni(NO_3)_2·4H_2O$, 97.0%) were obtained from Sigma Aldrich (Saint Louis, MO, United States of America). Standard solutions of total Pb, Cd, Ni, Fe, and K (1,000.0 mg $L^{-1}$ for all standards) used for analytical calibration curves, were acquired from Fluka (Charlotte, NC, United States of America). Nitrogen ($N_2$, 99.0%) and acetylene ($C_2H_2$, 99.8%) gases were supplied by White Martins (Rio de Janeiro, RJ, Brazil). All experimental procedures were conducted using Milli-Q® ultrapure water (18.2 MΩ $cm^{-1}$).

## *1.2. Characterization of MACs-C*

The MACs-C (MAC-230-0-5, MAC-230-0-7, MAC-270-0-5, and MAC-270-0-7) are novelty, and their specific surface area, pore volume, and pore diameter were characterized in the present study. For these, $N_2$ adsorption-desorption measurements were performed using a Quantachrome Nova 1200 analyzer following the same procedure used for MACs in another study.[1]

## *1.3. Adsorption equations and procedures*

### 1.3.1. Effect of contact time

The nonlinear theoretical kinetic models of Pseudo-First Order (PFO - Equation S1), Pseudo-Second Order (PSO - Equation S2), Intraparticle Diffusion (ID - Equation S3), and Diffusion-Chemisorption (DC - Equation S4) were adjusted to the experimental data.

$$q_t = q_e\left(1 - e^{(-K_1 t)}\right) \tag{S1}$$

$$q_t = \frac{K_2 (q_e)^2 t}{1 + K_2 q_e t} \tag{S2}$$

$$q_t = K_{ID}\sqrt{t} + C \tag{S3}$$

$$q_t = \frac{q_e K_{DC}\sqrt{t}}{K_{DC}\sqrt{t} + q_e} \tag{S4}$$

where $q_e$ is the amount of Pb(II), Cd(II), and Ni(II) adsorbed at equilibrium (mmol $g^{-1}$), $t$ is time (min), $K_1$ ($min^{-1}$) and $K_2$ (g $mmol^{-1}$ $min^{-1}$) are the rate constants for the pseudo-first order (PFO) and pseudo-second order (PSO) kinetic models, respectively; $K_{ID}$ (mmol $g^{-1}$ $min^{0.5}$) is the intraparticle diffusion rate constant and $C$ (mmol $g^{-1}$) is the intercept, representing the boundary layer thickness; $K_{DC}$ (mmol $g^{-1}$ $min^{-0.5}$) is the diffusion-chemisorption rate constant.

The adjusted coefficient of determination (*Adj.* $R^2$ - Equation S5), root mean square error (*RMSE* - Equation S6), and chi-square ($\chi^2$ - Equation S7) functions were calculated to validate the model fit.

$$\text{Adj. } R^2 = 1 - (1 - R^2)\left(\frac{n_{exp} - 1}{n_{exp} - n_p - 1}\right) \tag{S5}$$

$$RMSE = \sqrt{\frac{1}{n_{exp} - 1}\sum (q_{exp} - q_{cal})^2} \tag{S6}$$

$$\chi^2 = \sum \frac{(q_{exp} - q_{cal})^2}{(q_{cal})^2} \tag{S7}$$

where $R^2$ is the coefficient of determination, $n_{exp}$ is the number of experimental data points, $n_p$ is the number of parameters in each kinetic model (PFO, PSO, ID, and DC = 2 parameters), $q_{exp}$ is the experimentally adsorbed amount of Pb(II), Cd(II), and Ni(II) (mmol $g^{-1}$), and $q_{cal}$ is the amount adsorbed calculated for Pb(II), Cd(II), and Ni(II) (mmol $g^{-1}$) according to the kinetic models.

1.3.2. Effect of concentration

The adsorption isotherm was evaluated using the nonlinear models of Freundlich (FR - Equation S8), Langmuir (LA - Equation S9), Extended Langmuir (ELA - Equation S10), Sips (SI - Equation S11), Extended Sips (ESI - Equation S12), and Temkin (TE - Equation S13).

$$q_e = K_F \times C_e^{1/n_F} \tag{S8}$$

$$q_e = \frac{q_m \times K_L \times C_e}{1 + K_L \times C_e} \tag{S9}$$

$$q_{e,i} = \frac{q_{m,i} \times K_{L,i} \times C_{e,i}}{1 + \sum_{j=1}^{n} K_{L,i} \times C_{e,i}} \tag{S10}$$

$$q_e = \frac{q_{ms} \times K_s \times C_e^{ns}}{1 + K_s \times C_e^{ns}} \tag{S11}$$

$$q_{e,i} = \frac{q_{ms,i} \times K_{s,i} \times C_{e,i}^{ns,i}}{1 + \sum_{j=1}^{n} K_{s,i} \times C_{e,i}^{ns,i}} \tag{S12}$$

$$q_e = \frac{RT}{a} \ln(K_T C_e) \tag{S13}$$

where $K_F$ [(mmol $g^{-1}$)(mmol $L^{-1}$)$^{-1/n}$] and $n_F$ (dimensionless) are the Freundlich constants, $C_e$ is the equilibrium concentration (mmol $L^{-1}$), $q_m$ is the adsorption capacity estimated by the Langmuir model (mmol $g^{-1}$), $K_L$ (L $mmol^{-1}$) is the Langmuir constant, $q_{ms}$ is the adsorption capacity estimated by the Sips model (mmol $g^{-1}$), $K_s$ ($L^{ns}$ $mmol^{-ns}$) and $n_s$ (dimensionless) are the Sips constants, $i$ refers to the i-th component, and $j$ represents the remaining components. $R$ is the universal gas constant (8.314 J $mol^{-1}$ $K^{-1}$), $T$ is the absolute temperature (293.15 K), $a$ is a constant related to adsorption energy (J $mol^{-1}$), and $K_T$ is the Temkin constant related to adsorbate-adsorbent affinity (L $mmol^{-1}$).

For the Extended Langmuir (Equation S10) and Extended Sips (Equation S12) models, the constants and estimated adsorption capacities were calculated using the classical nonlinear models (Equations S9 and S11, respectively) and combined into three separate equations (one for each component in this ternary system), yielding new adsorption capacities ($q_{e,i}$), and nonlinear fits. The error functions Adj. $R^2$, RMSE, and $\chi^2$ were calculated to validate the isotherm model fittings, as defined by Equations S5-S7.

An analogous experiment to the concentration effect was performed under optimized adsorption conditions (pH 5.0; adsorbent dose: 0.5 g $L^{-1}$; contact time: 30 min; initial concentrations of Pb(II), Cd(II), and Ni(II): 100.0 mmol $L^{-1}$ of each metal) using a buffered solution of 0.5 mol $L^{-1}$ acetic acid and 0.9 mol $L^{-1}$ sodium acetate. This setup aimed to enable a comparative analysis between buffered and unbuffered systems and to evaluate the impact of pH stabilization at the optimal condition.

1.3.3. Thermodynamic parameters

The adsorption equilibrium constant ($K_{mod}$) was derived from the isotherm model that provided the best fit to the experimental data. However, since the constants provided by isotherm models are not dimensionless, conversion to a dimensionless form ($K$ₑ⁰) was required, using Equation S14.[2] To ensure thermodynamic consistency across different metal ions and temperatures, special attention was given to the dimensional analysis of the Sips isotherm parameter, $K_s$. The operational unit of $K_s$ depends directly on the fitted heterogeneity exponent $n_s$, being expressed as $(L\ mmol^{-1})^{n_s}$. To render this parameter dimensionless regardless of variations in $n_s$, $K_s$ was scaled using the standard reference concentration $[ADVT]^\circ = 1.0$ mol $L^{-1}$ = 1000.0 mmol $L^{-1}$ raised to the power of $n_s$ (Equation S14a).

$$K_{mod} = K_s \times (ADVT^\circ)^{ns} \quad \text{(S14a)}$$

The dimensional verification of Equation S14a demonstrates that $K_{mod}$ becomes inherently dimensionless:

$$[K_{mod}] = [K_s] \times [[ADVT]^\circ]^{n_s} = \left(\frac{L}{mmol}\right)^{n_s} \times \left(\frac{mmol}{L}\right)^{n_s} = \left(\frac{L}{mmol} \times \frac{mmol}{L}\right)^{n_s} = 1^{n_s} = 1 \quad \text{(S14b)}$$

Because the term inside the brackets cancels out completely to unity ($1^{n_s} = 1$), $K_{mod}$ is guaranteed to be dimensionless for any fitted exponent $n_s$, metal ion, or temperature system.

When activity coefficients (γ) are explicitly incorporated at equilibrium, the dimensionless equilibrium constant $K_e^\circ$ is defined as presented in Equation S14.

$$K_e{}^\circ = \frac{K_{mod} \times MM_{ADVT} \times [ADVT]^\circ}{□} \quad \text{(S14)}$$

where $K_{mod}$ (dimensionless) represents the equilibrium constant determined from the SI model, which provided the best fit to the experimental data at temperature $T$ (K); $[ADVT]^\circ$ is the standard adsorbate concentration (1.0 mol $L^{-1}$), $MM_{ADVT}$ is the adsorbate molecular weight (g $mol^{-1}$), and γ is the activity coefficient (dimensionless).

For highly dilute adsorbate solutions, the activity coefficient (γ) is considered unity. However, in the case of ternary solutions, it was necessary to determine γ at the equilibrium concentration (100.0 mmol $L^{-1}$ = 0.1 mol $L^{-1}$) based on the ionic strength ($I$), calculated by Equation S15, using the Debye-Hückel equation as presented in Equation S16.[2–6]

$$I = \frac{1}{2} ∑_i c_i Z_i{}^2 \quad \text{(S15)}$$

$$\log \gamma_i = -\frac{A Z_i^2 \sqrt{I}}{1 + B a_i \sqrt{I}} \quad \text{(S16)}$$

where $c_i$ is the molar concentration of the i-th ion (mol $L^{-1}$) and $Z_i$ is its charge, $A$ is a temperature-dependent constant (mol$^{-0.5}$ L$^{0.5}$ K$^{-1.5}$; 21 °C = 0.522; 29 °C = 0.517; 34 °C = 0.516; 44 °C = 0.506), $a_i$ is the effective hydrated ionic radius (Pb(II) = 0.401 nm; Cd(II) = 0.426 nm; Ni(II) = 0.404 nm), and B is also a temperature-dependent constant (nm$^{-1}$ mol$^{-0.5}$ L$^{0.5}$ K$^{-0.5}$; 21 °C = 0.331; 29 °C = 0.330; 34 °C = 0.329; 44 °C = 0.327). The calculated ionic strength (I) reached 0.9 mol $L^{-1}$ for the concentrated ternary metal system (100.0 mmol $L^{-1}$). Although the Debye–Hückel treatment (Equation S16) is strictly reliable for dilute solutions ($I < 0.1$ mol $L^{-1}$), it was applied here as a semi-quantitative approximation to capture electrostatic activity corrections in the multicomponent matrix. Consequently, the derived absolute thermodynamic parameters should be regarded as model-dependent estimates rather than exact thermodynamic quantities.

The combination of the Van't Hoff equation (Equation S17) with the equation derived from the Third Law of Thermodynamics (Equation S18) led to the formulation of Equation S19, which was used to determine ΔH and ΔS values through linear regression of the plot of $ln\ K_e^\circ$ versus $1/T$. The ΔG value was subsequently calculated after determining ΔH and ΔS using Equation S18.[2]

$$\Delta G^\circ = -RT \ln K_e^\circ \quad \text{(S17)}$$

$$\Delta G^\circ = \Delta H^\circ - T\Delta S^\circ \quad \text{(S18)}$$

$$\ln K_e^\circ = \left(\frac{-\Delta H^\circ}{R}\right)\frac{1}{T} + \frac{\Delta S^\circ}{R} \quad \text{(S19)}$$

where $R$ is the universal gas constant (8.314 J mol$^{-1}$ K$^{-1}$), $T$ is the absolute temperature (K), and $K_e^\circ$ is the dimensionless equilibrium constant.

## 2. Tables

**Table S1.** Instrumental conditions for the determination of total lead (Pb), cadmium (Cd), nickel (Ni), total iron (Fe), and total potassium (K) using FAAS, according to the manufacturer.

| Parameters | Elements | | | | |
|---|---|---|---|---|---|
| | Pb | Cd | Ni | Fe | K |
| Lamp current (mA) | 5.0 | 5.0 | 10.0 | 10.0 | - |
| Wavelength (nm) | 283.3 | 228.8 | 352.5 | 248.3 | 766.5 |
| Slot opening (nm) | 1.0 | 0.2 | 0.2 | 0.2 | 0.2 |
| Acetylene gas flow (mL min$^{-1}$) | 2.00 | 2.00 | 2.00 | 2.00 | 2.00 |

| | | | | | |
|---|---|---|---|---|---|
| Compressed air flow (mL min$^{-1}$) | 13.50 | 13.50 | 13.50 | 13.50 | 13.50 |
| Mode | AB* | AB* | AB* | AB* | EM** |

(-) Not employed; *Absorbance (AB); **Emission (EM).

**Table S2.** Textural properties of MACs-C obtained from $N_2$ adsorption-desorption isotherms at -196 °C.

| Materials | BET[a] | BJH[b] | |
|---|---|---|---|
| | Specific surface area (m$^2$ g$^{-1}$) | Total pore volume (cm$^3$ g$^{-1}$) | Average pore diameter (nm) |
| MAC-230-0-5 | 99 | 0.13 | 0.4 |
| MAC-230-0-7 | 220 | 0.20 | 0.4 |
| MAC-270-0-5 | 72 | 0.03 | 0.4 |
| MAC-270-0-7 | 236 | 0.21 | 0.4 |

[a] Calculated by the BET model; [b] Calculated by the BJH model.

**Table S3.** Adjusted initial and final pH values obtained in the multicomponent adsorption study of Pb(II), Cd(II), and Ni(II) by MAC-270-2-7 as a function of pH, including the controls (multicomponent solutions only).

| Target pH | Initial pH | Final pH of adsorption experiments | Final pH of the multicomponent solution controls |
|---|---|---|---|
| pH 2.0 | pH 2.0 | 2.1±0.04 | 1.9±0.04 |
| pH 3.0 | pH 3.0 | 3.3±0.02 | 3.2±0.06 |
| pH 4.0 | pH 4.1 | 4.5±0.08 | 4.2±0.08 |
| pH 5.0 | pH 4.9 | 4.6±0.04 | 4.9±0.04 |
| pH 6.0 | pH 6.1 | 4.8±0.06 | 5.5±0.06 |

**Table S4.** Kinetic adsorption parameters obtained by nonlinear regression of the PFO, PSO, ID, and DC kinetic models, as well as $R^2$, RMSE, and □². The underlined data are the main parameters evaluated in the decision-making about the model in which the data best fit; the highlights in bold blue represent the models with the best fit.

| Kinetic models | 0.25 mmol L$^{-1}$ | | | 10.0 mmol L$^{-1}$ | | | 100.0 mmol L$^{-1}$ | | |
|---|---|---|---|---|---|---|---|---|---|
| | Pb(II) | Cd(II) | Ni(II) | Pb(II) | Cd(II) | Ni(II) | Pb(II) | Cd(II) | Ni(II) |
| **PFO** | | | | | | | | | |
| $q_e$ (mmol g$^{-1}$) | 0.118 | 0.007 | 0.016 | 0.584 | 0.208 | 0.245 | **0.822** | **1.080** | **1.382** |
| $K_1$ (min$^{-1}$) | 0.587 | 15.718 | 0.309 | 378.92 | 80.38 | 1.168 | **0.640** | **50.26** | **2.856** |
| *$R^2$* | 0.983 | 0.738 | 0.875 | 0.987 | 0.928 | 0.981 | **0.999** | **0.997** | **0.982** |
| *Adj. $R^2$* | 0.975 | 0.607 | 0.813 | 0.981 | 0.892 | 0.972 | **0.999** | **0.996** | **0.973** |
| □² | 3.39*10$^{-5}$ | 2.02*10$^{-3}$ | 7.86*10$^{-4}$ | 8.76*10$^{-8}$ | 1.24*10$^{-6}$ | 1.78*10$^{-9}$ | **3.11*10$^{-5}$** | **1.38*10$^{-7}$** | **6.17*10$^{-9}$** |
| *RMSE* | 2.81*10$^{-4}$ | 1.28*10$^{-4}$ | 1.83*10$^{-4}$ | 7.06*10$^{-5}$ | 9.45*10$^{-5}$ | 1.34*10$^{-4}$ | **1.87*10$^{-3}$** | **1.64*10$^{-4}$** | **4.43*10$^{-5}$** |
| **PSO** | | | | | | | | | |
| $q_e$ (mmol g$^{-1}$) | 0.121 | 0.006 | 0.016 | 0.597 | 0.212 | 0.250 | 0.824 | 1.077 | 1.359 |
| $K_2$ (g mmol$^{-1}$ min$^{-1}$) | 16.09 | 7.04*10$^{25}$ | 57.37 | 1.04*10$^{22}$ | 4.32*10$^{22}$ | 21.67 | 5.183 | 1.57*10$^{23}$ | 9.66*10$^{14}$ |

| | | | | | | | | | |
|---|---|---|---|---|---|---|---|---|---|
| *$R^2$* | 0.982 | 0.700 | 0.863 | 0.983 | 0.926 | 0.979 | 0.999 | 0.997 | 0.980 |
| *Adj. $R^2$* | 0.973 | 0.550 | 0.795 | 0.975 | 0.889 | 0.972 | 0.999 | 0.996 | 0.970 |
| *$\square^2$* | $9.29*10^{-4}$ | $1.31*10^{-2}$ | $7.86*10^{-4}$ | $4.87*10^{-4}$ | $3.98*10^{-4}$ | $4.54*10^{-4}$ | $6.39*10^{-5}$ | $9.97*10^{-6}$ | $2.89*10^{-4}$ |
| *RMSE* | $1.51*10^{-3}$ | $2.80*10^{-4}$ | $1.83*10^{-4}$ | $5.38*10^{-3}$ | $1.73*10^{-3}$ | $2.17*10^{-3}$ | $2.69*10^{-3}$ | $1.39*10^{-3}$ | $9.43*10^{-3}$ |
| **ID** | | | | | | | | | |
| $K_{ID}$ (mmol $g^{-1}$ $min^{-0,5}$) | 0.002 | $2.06*10^{-5}$ | $1.78*10^{-4}$ | 0.007 | 0.002 | 0.003 | 0.010 | 0.012 | 0.012 |
| C (mmol $g^{-1}$) | 0.079 | 0.006 | 0.011 | 0.415 | 0.152 | 0.169 | 0.574 | 0.768 | 1.034 |
| *$R^2$* | 0.270 | 0.010 | 0.160 | 0.185 | 0.134 | 0.231 | 0.206 | 0.186 | 0.110 |
| *Adj. $R^2$* | -0.095 | -0.485 | -0.260 | -0.223 | -0.299 | -0.154 | -0.191 | -0.221 | -0.335 |
| *$\square^2$* | - | - | - | - | - | - | - | - | - |
| *RMSE* | - | - | - | - | - | - | - | - | - |
| **DC** | | | | | | | | | |
| $q_e$ (mmol $g^{-1}$) | 0.124 | 0.007 | 0.016 | 0.595 | 0.208 | 0.253 | **0.830** | **1.080** | **1.382** |
| $K_{DC}$ (mmol $g^{-1}$ $min^{-0,5}$) | 0.366 | $1.72*10^{45}$ | 0.043 | 123.16 | 4.819 | 1.544 | **7.320** | **$2.51*10^{41}$** | **$3.99*10^{44}$** |
| *$R^2$* | 0.984 | 0.738 | 0.851 | 0.984 | 0.928 | 0.980 | **0.999** | **0.997** | **0.979** |
| *Adj. $R^2$* | 0.976 | 0.607 | 0.777 | 0.976 | 0.892 | 0.970 | **0.999** | **0.996** | **0.969** |
| *$\square^2$* | $2.91*10^{-3}$ | $2.02*10^{-3}$ | $7.86*10^{-4}$ | $3.53*10^{-4}$ | $1.24*10^{-6}$ | $1.08*10^{-3}$ | **$2.30*10^{-4}$** | **$1.38*10^{-7}$** | **$5.11*10^{-4}$** |
| *RMSE* | $2.73*10^{-3}$ | $1.28*10^{-4}$ | $1.83*10^{-4}$ | $4.56*10^{-3}$ | $9.45*10^{-5}$ | $3.40*10^{-3}$ | **$5.14*10^{-3}$** | **$1.64*10^{-4}$** | **$4.43*10^{-5}$** |

(-) Not calculated. All models were fitted using non-linear regression; however, non-linear error functions ($\square^2$ and RMSE) were not applied to the Intraparticle Diffusion (ID) model or specific Pseudo-Second Order (PSO) fits due to model formulation constraints.

**Table S5.** Isothermal adsorption parameters obtained from the nonlinear regression of the isothermal models of FR, LA, ELA, SI, ESI, and TE, as well as $R^2$, RMSE, and $\square^2$. The underlined data are the main parameters evaluated in the decision-making about the model in which the data best fit; the highlights in bold blue represent the models with the best fit.

| Isothermal models | Pb(II) | Cd(II) | Ni(II) |
|---|---|---|---|
| **FR** | | | |
| $n_F$ | 3.882 | 1.542 | 1.584 |
| $K_F$ [(mmol $g^{-1}$) (mmol $L^{-1})^{-1/n}$] | 0.277 | 0.086 | 0.097 |
| *$R^2$* | 0.944 | 0.944 | 0.941 |
| *Adj. $R^2$* | 0.922 | 0.922 | 0.917 |
| *$\square^2$* | - | - | - |
| *RMSE* | - | - | - |
| **LA** | | | |
| $q_m$ (mmol $g^{-1}$) | 0.870 | 1.958 | 2.583 |
| $K_L$ (L $mmol^{-1}$) | 0.166 | 0.025 | 0.018 |

| | | | |
|---|---|---|---|
| *$R^2$* | 0.961 | 0.968 | 0.967 |
| *Adj. $R^2$* | 0.945 | 0.955 | 0.954 |
| $\square^2$ | $8.11*10^{-3}$ | $2.08*10^{-1}$ | $1.92*10^{-1}$ |
| *RMSE* | $2.96*10^{-2}$ | $3.37*10^{-1}$ | $4.28*10^{-1}$ |
| ELA | | | |
| $q_m$ (mmol $g^{-1}$) | 0.727 | 0.156 | 0.219 |
| $K_L$ (L $mmol^{-1}$) | 0.194 | 0.347 | 0.234 |
| *$R^2$* | 0.895 | - | - |
| *Adj. $R^2$* | 0.884 | - | - |
| $\square^2$ | $7.86*10^{-3}$ | 34.2 | 31.6 |
| *RMSE* | $2.44*10^{-2}$ | $3.44*10^{-1}$ | $4.65*10^{-1}$ |
| SI | | | |
| $q_{ms}$ (mmol $g^{-1}$) | **1.076** | **1.269** | **1.666** |
| $K_s$ ($L^{ns}$ $mmol^{-ns}$) | **0.269** | **0.008** | **0.003** |
| $n_s$ | **0.566** | **1.702** | **1.825** |
| *$R^2$* | **0.967** | **0.982** | **0.984** |
| *Adj. $R^2$* | **0.942** | **0.969** | **0.972** |
| $\square^2$ | **$6.99*10^{-2}$** | **$2.56*10^{-2}$** | **$1.68*10^{-2}$** |
| *RMSE* | **$1.08*10^{-1}$** | **$7.67*10^{-2}$** | **$8.17*10^{-2}$** |
| ESI | | | |
| $q_{ms}$ (mmol $g^{-1}$) | 0.348 | 0.493 | 0.984 |
| $K_s$ ($L^{ns}$ $mmol^{-ns}$) | 10.83 | 0.015 | 0.006 |
| $n_s$ | 1.774 | 1.524 | 1.413 |
| *$R^2$* | - | 0.308 | 0.461 |
| *Adj. $R^2$* | - | 0.290 | 0.444 |
| $\square^2$ | 16.1 | 13.5 | $2.25*10^{-1}$ |
| *RMSE* | $1.67*10^{-1}$ | $2.16*10^{-1}$ | $1.76*10^{-1}$ |
| TE | | | |
| *a* (J $mol^{-1}$) | 22913.01 | 25815.13 | 18834.36 |
| $K_T$ (L $mmol^{-1}$) | 19.91 | 31.61 | 22.09 |
| *$R^2$* | 0.923 | 0.566 | 0.576 |
| *Adj. $R^2$* | 0.892 | 0.392 | 0.406 |
| $\square^2$ | - | - | - |

| | | | |
|---|---|---|---|
| *RMSE* | - | - | - |

(-) Not calculated. All models were fitted using non-linear regression; however, non-linear error functions (χ$^2$ and RMSE) were not applicable to linear/extended forms where parameter convergence did not yield meaningful non-linear error statistics.

**Table S6.** Adsorption isotherm parameters obtained as a function of temperature using nonlinear regression of the FR, LA, ELA, SI, ESI, and TE isotherm models, as well as $R^2$, RMSE, and χ$^2$. The highlights in bold blue represent the models with the best fit.

| Isothermal models | 21.0 °C | | |
|---|---|---|---|
| | Pb(II) | Cd(II) | Ni(II) |
| **FR** | | | |
| $n_F$ | 3.882 | 1.542 | 1.584 |
| $K_F$ [(mmol g$^{-1}$) (mmol L$^{-1}$)$^{-1/n}$] | 0.277 | 0.086 | 0.097 |
| *$R^2$* | 0.944 | 0.944 | 0.941 |
| *Adj. $R^2$* | 0.922 | 0.922 | 0.917 |
| *□$^2$* | - | - | - |
| *RMSE* | - | - | - |
| **LA** | | | |
| $q_m$ (mmol g$^{-1}$) | 0.870 | 1.958 | 2.583 |
| $K_L$ (L mmol$^{-1}$) | 0.166 | 0.025 | 0.018 |
| *$R^2$* | 0.961 | 0.968 | 0.967 |
| *Adj. $R^2$* | 0.945 | 0.955 | 0.954 |
| *□$^2$* | 8.11*10$^{-3}$ | 2.08*10$^{-1}$ | 1.92*10$^{-1}$ |
| *RMSE* | 2.96*10$^{-2}$ | 3.37*10$^{-1}$ | 4.28*10$^{-1}$ |
| **ELA** | | | |
| $q_m$ (mmol g$^{-1}$) | 0.727 | 0.156 | 0.219 |
| $K_L$ (L mmol$^{-1}$) | 0.194 | 0.347 | 0.234 |
| *$R^2$* | 0.895 | - | - |
| *Adj. $R^2$* | 0.884 | - | - |
| *□$^2$* | 7.86*10$^{-3}$ | 34.2 | 31.6 |
| *RMSE* | 2.44*10$^{-2}$ | 3.44*10$^{-1}$ | 4.65*10$^{-1}$ |

| | | | |
|---|---|---|---|
| **SI** | | | |
| $q_{ms}$ (mmol g$^{-1}$) | **1.076** | **1.269** | **1.666** |
| $K_s$ ($L^{ns}$ mmol$^{-ns}$) | **0.269** | **0.008** | **0.003** |
| $n_s$ | **0.566** | **1.702** | **1.825** |
| $R^2$ | **0.967** | **0.982** | **0.984** |
| *Adj.* $R^2$ | **0.942** | **0.969** | **0.972** |
| $\chi^2$ | **6.99*10$^{-2}$** | **2.56*10$^{-2}$** | **1.68*10$^{-2}$** |
| *RMSE* | **1.08*10$^{-1}$** | **7.67*10$^{-2}$** | **8.17*10$^{-2}$** |
| **ESI** | | | |
| $q_{ms}$ (mmol g$^{-1}$) | 0.348 | 0.493 | 0.984 |
| $K_s$ ($L^{ns}$ mmol$^{-ns}$) | 10.83 | 0.015 | 0.006 |
| $n_s$ | 1.774 | 1.524 | 1.413 |
| $R^2$ | - | 0.308 | 0.461 |
| *Adj.* $R^2$ | - | 0.290 | 0.444 |
| $\chi^2$ | 16.1 | 13.5 | 2.25*10$^{-1}$ |
| *RMSE* | 1.67*10$^{-1}$ | 2.16*10$^{-1}$ | 1.76*10$^{-1}$ |
| **TE** | | | |
| $a$ (J mol$^{-1}$) | 22913.01 | 25815.13 | 18834.36 |
| $K_T$ (L mmol$^{-1}$) | 19.91 | 31.61 | 22.09 |
| $R^2$ | 0.923 | 0.566 | 0.576 |
| *Adj.* $R^2$ | 0.892 | 0.392 | 0.406 |
| $\chi^2$ | - | - | - |
| *RMSE* | - | - | - |

| Isothermal models | 29.0 °C | | |
|---|---|---|---|
| | Pb(II) | Cd(II) | Ni(II) |
| **FR** | | | |
| $n_F$ | 3.185 | 1.732 | 1.729 |
| $K_F$ [(mmol g$^{-1}$) (mmol L$^{-1}$)$^{-1/n}$] | 0.322 | 0.123 | 0.172 |
| $R^2$ | 0.942 | 0.935 | 0.917 |
| *Adj.* $R^2$ | 0.919 | 0.909 | 0.884 |
| $\chi^2$ | - | - | - |
| *RMSE* | - | - | - |
| **LA** | | | |

| | | | |
|---|---|---|---|
| $q_m$ (mmol $g^{-1}$) | 1.383 | 1.850 | 3.152 |
| $K_L$ (L $mmol^{-1}$) | 0.098 | 0.035 | 0.025 |
| *$R^2$* | 0.975 | 0.963 | 0.954 |
| *Adj. $R^2$* | 0.965 | 0.948 | 0.936 |
| $\square^2$ | $2.15*10^{-2}$ | $1.43*10^{-1}$ | $1.48*10^{-1}$ |
| *RMSE* | $7.67*10^{-2}$ | $2.65*10^{-1}$ | $4.58*10^{-1}$ |
| ELA | | | |
| $q_m$ (mmol $g^{-1}$) | 0.728 | 0.158 | 0.213 |
| $K_L$ (L $mmol^{-1}$) | 0.195 | 0.337 | 0.226 |
| *$R^2$* | 0.528 | - | - |
| *Adj. $R^2$* | 0.339 | - | - |
| $\square^2$ | $3.85*10^{-1}$ | 39.4 | 65.7 |
| *RMSE* | $1.71*10^{-1}$ | $3.75*10^{-1}$ | $6.53*10^{-1}$ |
| SI | | | |
| $q_{ms}$ (mmol $g^{-1}$) | **1.240** | **1.276** | **2.130** |
| $K_s$ ($L^{ns}$ $mmol^{-ns}$) | **0.051** | **0.009** | **0.004** |
| $n_s$ | **1.483** | **1.860** | **2.041** |
| *$R^2$* | **0.980** | **0.987** | **0.983** |
| *Adj. $R^2$* | **0.965** | **0.977** | **0.970** |
| $\square^2$ | **$2.34*10^{-3}$** | **$9.75*10^{-3}$** | **$7.96*10^{-3}$** |
| *RMSE* | **$2.27*10^{-2}$** | **$4.76*10^{-2}$** | **$7.18*10^{-2}$** |
| ESI | | | |
| $q_{ms}$ (mmol $g^{-1}$) | 0.360 | 0.528 | 0.925 |
| $K_s$ ($L^{ns}$ $mmol^{-ns}$) | 11.16 | 0.015 | 0.006 |
| $n_s$ | 1.749 | 1.478 | 1.449 |
| *$R^2$* | - | 0.207 | 0.090 |
| *Adj. $R^2$* | - | - | - |
| $\square^2$ | 51.9 | 13.9 | 12.0 |
| *RMSE* | $3.10*10^{-1}$ | $2.35*10^{-1}$ | $3.84*10^{-1}$ |
| TE | | | |
| *a* (J $mol^{-1}$) | 23630.45 | 25811.08 | 15485.23 |
| $K_T$ (L $mmol^{-1}$) | 190.55 | 57.95 | 45.12 |
| *$R^2$* | 0.766 | 0.570 | 0.543 |

| | | | |
|---|---|---|---|
| *Adj. $R^2$* | 0.672 | 0.398 | 0.360 |
| $\chi^2$ | - | - | - |
| *RMSE* | - | - | - |

| Isothermal models | 34.0 °C | | |
|---|---|---|---|
| | Pb(II) | Cd(II) | Ni(II) |
| FR | | | |
| $n_F$ | 2.524 | 1.854 | 1.780 |
| $K_F$ [(mmol $g^{-1}$) (mmol $L^{-1}$)$^{-1/n}$] | 0.362 | 0.151 | 0.231 |
| *$R^2$* | 0.940 | 0.939 | 0.914 |
| *Adj. $R^2$* | 0.916 | 0.915 | 0.880 |
| $\chi^2$ | - | - | - |
| *RMSE* | - | - | - |
| LA | | | |
| $q_m$ (mmol $g^{-1}$) | 2.377 | 1.823 | 3.746 |
| $K_L$ (L $mmol^{-1}$) | 0.062 | 0.042 | 0.028 |
| *$R^2$* | 0.981 | 0.969 | 0.955 |
| *Adj. $R^2$* | 0.973 | 0.957 | 0.937 |
| $\chi^2$ | $4.55*10^{-2}$ | $1.21*10^{-1}$ | $1.37*10^{-1}$ |
| *RMSE* | $1.92*10^{-1}$ | $2.39*10^{-1}$ | $5.24*10^{-1}$ |
| ELA | | | |
| $q_m$ (mmol $g^{-1}$) | 0.724 | 0.166 | 0.213 |
| $K_L$ (L $mmol^{-1}$) | 0.196 | 0.320 | 0.232 |
| *$R^2$* | 0.094 | - | - |
| *Adj. $R^2$* | - | - | - |
| $\chi^2$ | 25.1 | 38.1 | 102.0 |
| *RMSE* | $4.33*10^{-1}$ | $3.87*10^{-1}$ | $8.11*10^{-1}$ |
| SI | | | |
| $q_{ms}$ (mmol $g^{-1}$) | 2.013 | 1.388 | 2.538 |
| $K_s$ ($L^{ns}$ $mmol^{-ns}$) | 0.032 | 0.018 | 0.012 |
| $n_s$ | 1.550 | 1.541 | 2.186 |
| *$R^2$* | 0.990 | 0.972 | 0.988 |
| *Adj. $R^2$* | 0.983 | 0.951 | 0.979 |
| $\chi^2$ | $5.05*10^{-3}$ | $2.03*10^{-2}$ | $4.92*10^{-3}$ |

| | | | |
|---|---|---|---|
| *RMSE* | $5.40*10^{-2}$ | $7.48*10^{-2}$ | $6.73*10^{-2}$ |
| ESI | | | |
| $q_{ms}$ (mmol $g^{-1}$) | 0.367 | 0.525 | 0.931 |
| $K_s$ ($L^{ns}$ $mmol^{-ns}$) | 10.81 | 0.013 | 0.006 |
| $n_s$ | 1.751 | 1.566 | 1.415 |
| $R^2$ | - | 0.128 | - |
| *Adj.* $R^2$ | - | - | - |
| $\square^2$ | 16.8 | 16.0 | 23.6 |
| *RMSE* | $5.68*10^{-1}$ | $2.51*10^{-1}$ | $5.40*10^{-1}$ |
| TE | | | |
| *a* (J $mol^{-1}$) | 15054.95 | 20752.29 | 10259.15 |
| $K_T$ (L $mmol^{-1}$) | 105.69 | 29.48 | 17.12 |
| $R^2$ | 0.693 | 0.658 | 0.624 |
| *Adj.* $R^2$ | 0.570 | 0.521 | 0.474 |
| $\square^2$ | - | - | - |
| *RMSE* | - | - | - |

| Isothermal models | 44.0 °C | | |
|---|---|---|---|
| | Pb(II) | Cd(II) | Ni(II) |
| FR | | | |
| $n_F$ | 2.340 | 2.055 | 2.029 |
| $K_F$ [(mmol $g^{-1}$) (mmol $L^{-1}$)$^{-1/n}$] | 0.443 | 0.196 | 0.331 |
| $R^2$ | 0.934 | 0.951 | 0.937 |
| *Adj.* $R^2$ | 0.908 | 0.931 | 0.912 |
| $\square^2$ | - | - | - |
| *RMSE* | - | - | - |
| LA | | | |
| $q_m$ (mmol $g^{-1}$) | 3.460 | 1.761 | 3.625 |
| $K_L$ (L $mmol^{-1}$) | 0.052 | 0.055 | 0.039 |
| $R^2$ | 0.973 | 0.976 | 0.977 |
| *Adj.* $R^2$ | 0.962 | 0.966 | 0.968 |
| $\square^2$ | $5.75*10^{-2}$ | $8.09*10^{-2}$ | $8.79*10^{-2}$ |
| *RMSE* | $3.14*10^{-1}$ | $1.89*10^{-1}$ | $4.06*10^{-1}$ |
| ELA | | | |

| | | | |
|---|---|---|---|
| $q_m$ (mmol $g^{-1}$) | 0.724 | 0.163 | 0.220 |
| $K_L$ (L $mmol^{-1}$) | 0.198 | 0.323 | 0.210 |
| *$R^2$* | - | - | - |
| *Adj. $R^2$* | - | - | - |
| *$\square^2$* | 69.3 | 45.3 | 112.0 |
| *RMSE* | $7.20*10^{-1}$ | $4.15*10^{-1}$ | $8.81*10^{-1}$ |
| **SI** | | | |
| $q_{ms}$ (mmol $g^{-1}$) | **2.872** | **1.526** | **2.834** |
| $K_s$ ($L^{ns}$ $mmol^{-ns}$) | **0.019** | **0.039** | **0.021** |
| $n_s$ | **1.536** | **1.255** | **1.647** |
| *$R^2$* | **0.982** | **0.978** | **0.990** |
| *Adj. $R^2$* | **0.969** | **0.962** | **0.983** |
| *$\square^2$* | **$7.10*10^{-3}$** | **$3.04*10^{-2}$** | **$1.00*10^{-2}$** |
| *RMSE* | **$9.15*10^{-2}$** | **$1.01*10^{-1}$** | **$1.07*10^{-1}$** |
| **ESI** | | | |
| $q_{ms}$ (mmol $g^{-1}$) | 0.357 | 0.505 | 0.951 |
| $K_s$ ($L^{ns}$ $mmol^{-ns}$) | 11.47 | 0.014 | 0.006 |
| $n_s$ | 1.750 | 1.554 | 1.438 |
| *$R^2$* | - | - | - |
| *Adj. $R^2$* | - | - | - |
| *$\square^2$* | 40.5 | 22.4 | 28.3 |
| *RMSE* | $8.59*10^{-1}$ | $2.85*10^{-1}$ | $6.04*10^{-1}$ |
| **TE** | | | |
| *a* (J $mol^{-1}$) | 10221.74 | 22229.30 | 10334.38 |
| $K_T$ (L $mmol^{-1}$) | 63.27 | 73.50 | 36.65 |
| *$R^2$* | 0.689 | 0.651 | 0.648 |
| *Adj. $R^2$* | 0.565 | 0.511 | 0.507 |
| *$\square^2$* | - | - | - |
| *RMSE* | - | - | - |

(-) Not calculated. All models were fitted using non-linear regression; however, non-linear error functions ($\square^2$ and RMSE) were not applicable to linear/extended forms where parameter convergence did not yield meaningful non-linear error statistics.

# 3. Figures

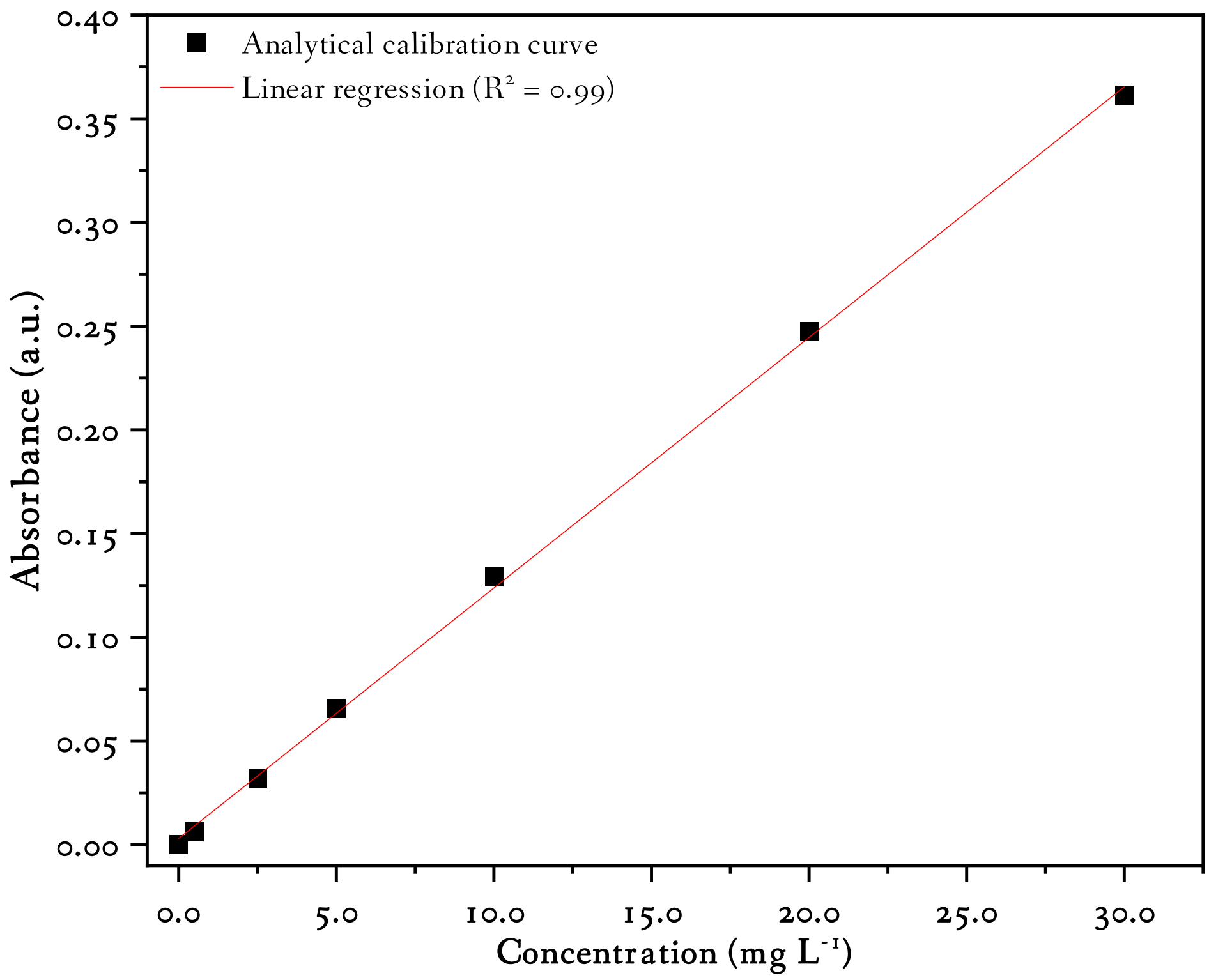
Analytical calibration curve
Linear regression ($R^2$ = 0.99)
Absorbance (a.u.)
0.00
0.05
0.10
0.15
0.20
0.25
0.30
0.35
0.40
0.0
5.0
10.0
15.0
20.0
25.0
30.0
Concentration (mg $L^{-1}$)

**Figure S1.** Analytical calibration curve obtained for total Pb by FAAS.

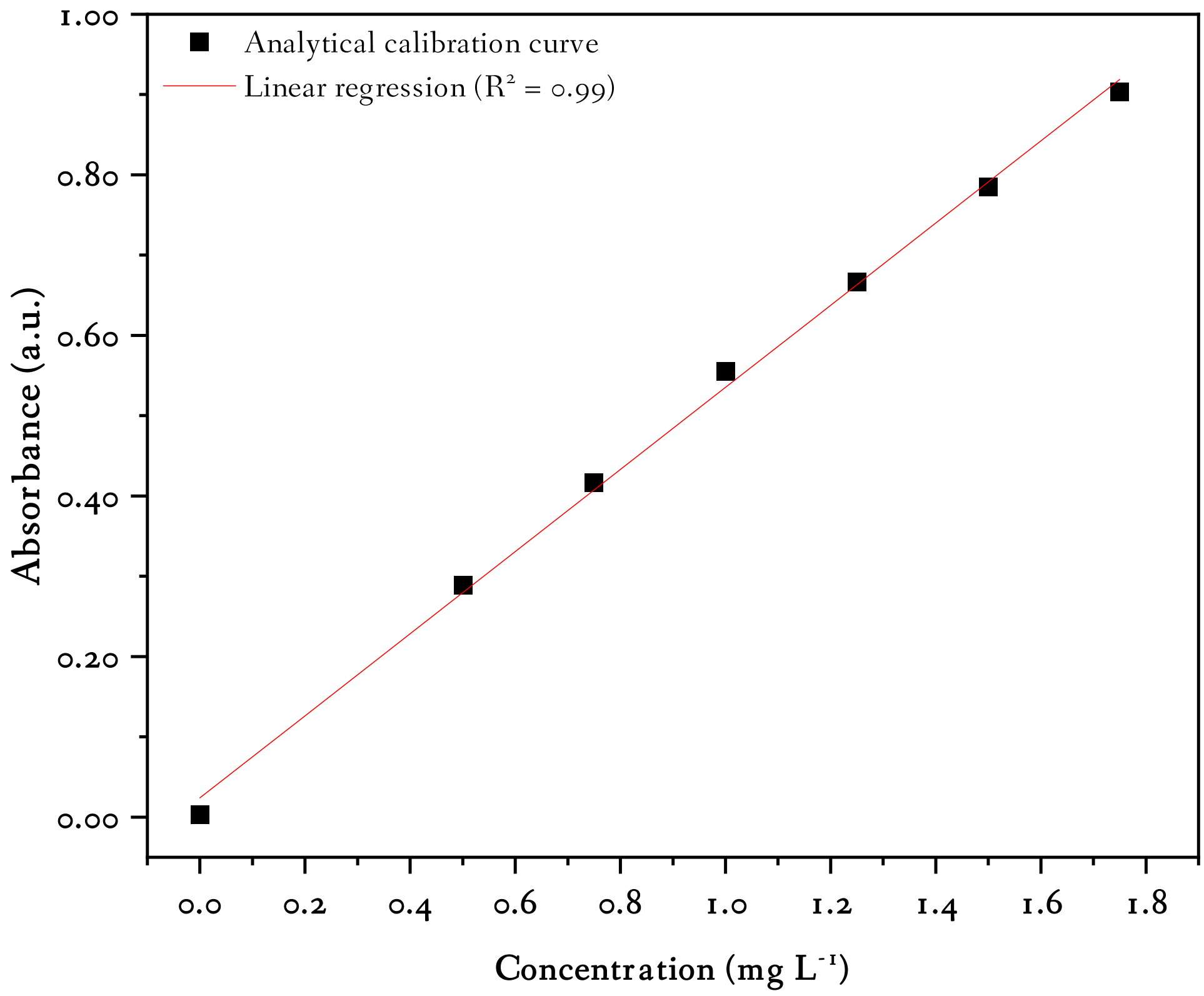


**Figure S2.** Analytical calibration curve obtained for total Cd by FAAS.

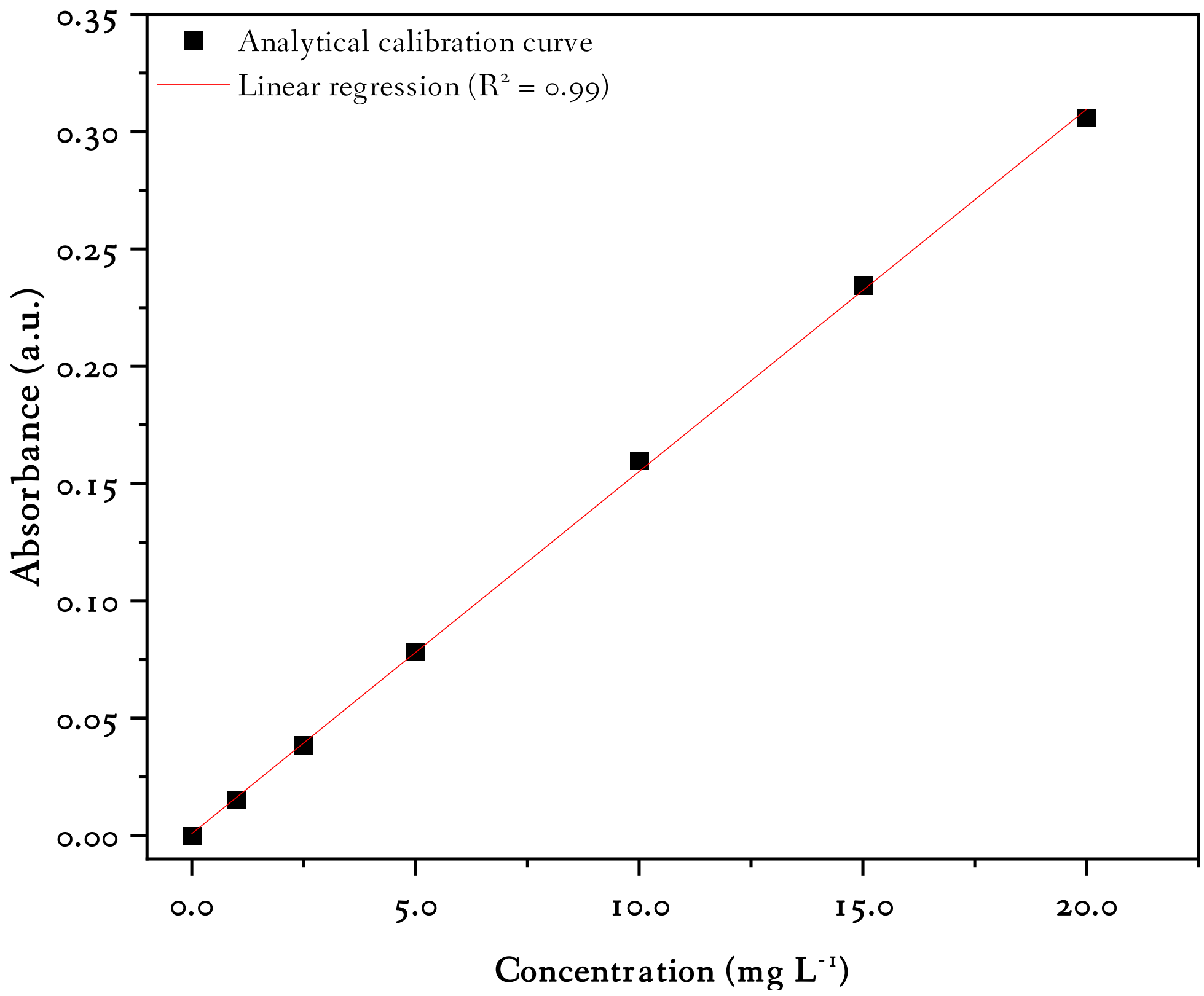


**Figure S3.** Analytical calibration curve obtained for total Ni by FAAS.

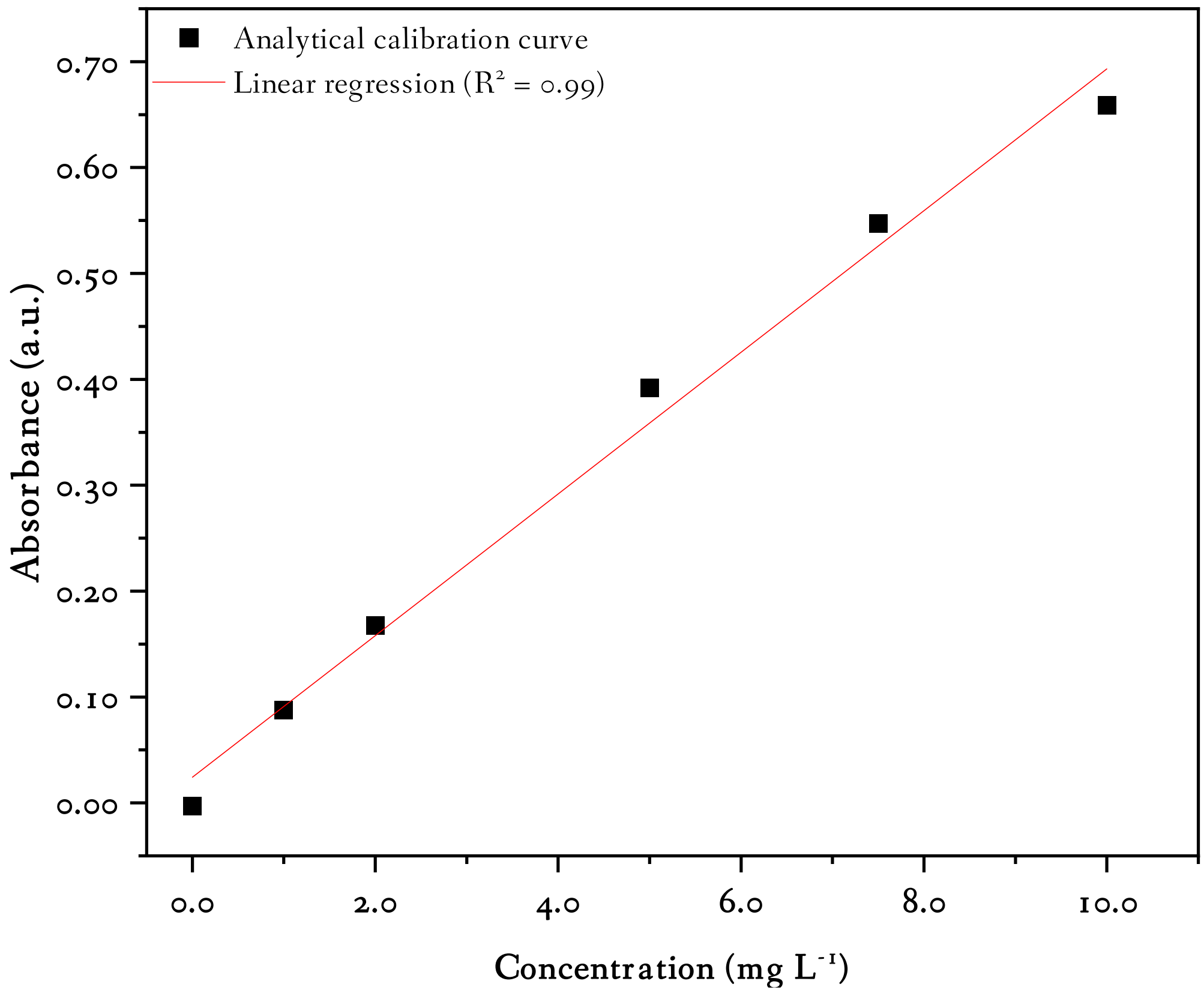


**Figure S4.** Analytical calibration obtained for total Fe by FAAS.

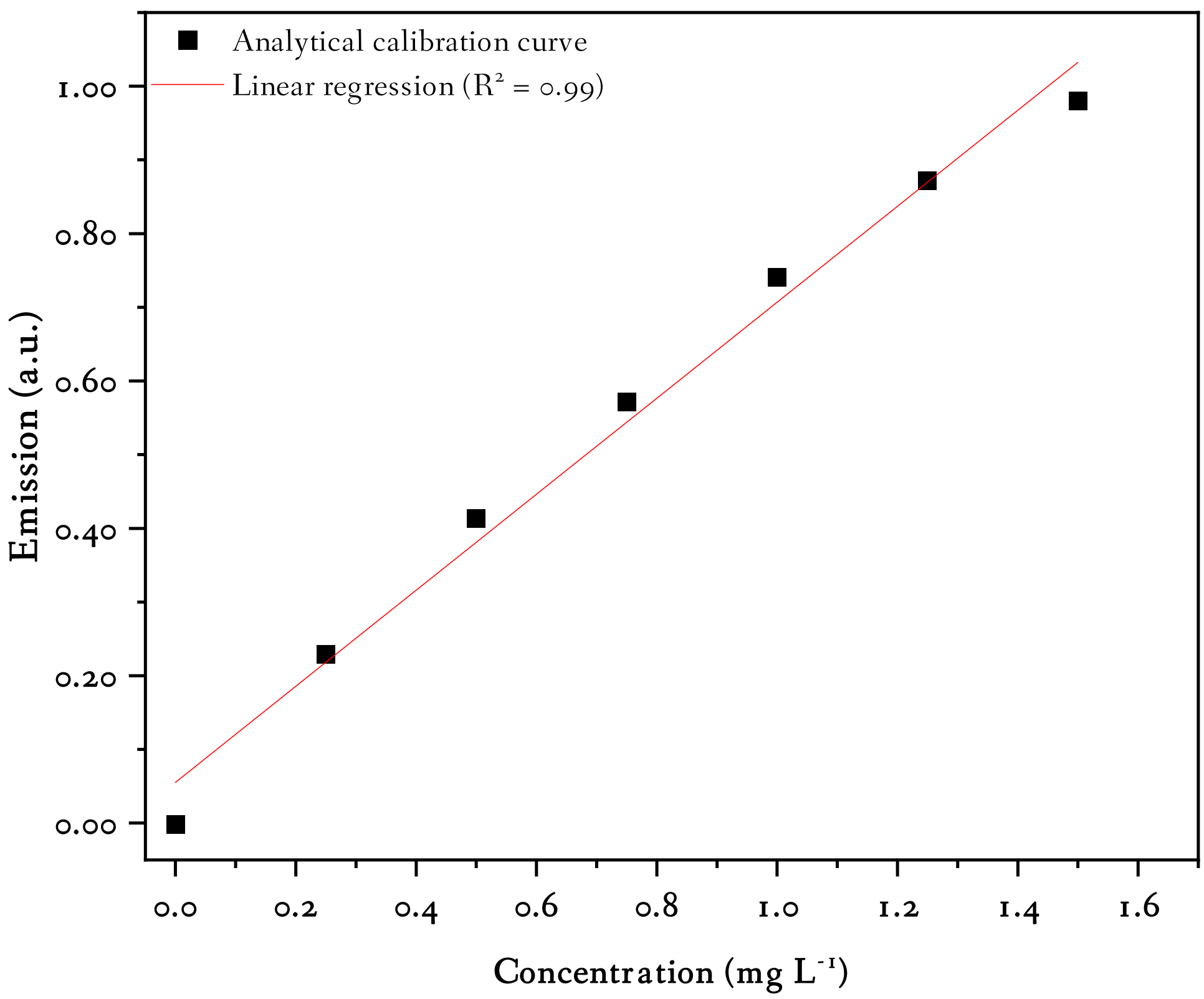


**Figure S5.** Analytical calibration curve obtained for total K by FAAS.

S3.1. *Evaluation of multi- and single-component adsorption*

Preliminary multicomponent adsorption experiments were conducted at initial concentrations of 0.02, 0.25, and 2.00 mmol $L^{-1}$ to identify the most efficient adsorbent under strong competitive conditions (Figure S6a-c). The intermediate initial concentration of 0.25 mmol $L^{-1}$ was selected to represent the competitive screening behavior, as it prevented complete metal depletion by the materials (Figure S6b). At this concentration, the non-activated MC-230 composite exhibited significant Pb(II) adsorption, reflecting a strong affinity for its surface functional groups, particularly the phenolic and lactonic moieties that were characterized in our previous study.[1] However, Cd(II) and Ni(II) adsorption was negligible at 0.25 mmol $L^{-1}$ (Figure S6b), suggesting that intense competition with Pb(II) effectively hinders their retention. The persistent substandard performance of MC-270 further indicates structural characteristics that are not conducive to metal binding, aligning with its lower phenolic content compared to MC-230, as established by our research group.[1]

At a temperature of 700 °C, the process of KOH activation resulted in an enhancement of the material's capacity for adsorption. The enhanced adsorption of Pb(II) in these activated systems can be attributed to its diminished hydrated radius and augmented polarizability, which favor heightened interactions with surface acidic functional groups (e.g., phenolic moieties).[1,7,8]

Regarding the control samples (MACs-C), materials activated at 500 °C demonstrated reduced adsorption capacities despite exhibiting larger surface areas (Table S2) in comparison to their respective MACs. As previously outlined in our structural and textural characterization,[1] this behavior is likely attributable to a predominantly microporous structure that restricts heavy metal accessibility to the active sites.[9,10] In contrast, MAC-270-2-7 exhibited superior performance, successfully adsorbing all three metals.

This high efficiency can be attributed to the structural and textural features previously detailed in our publication.[1] Specifically, the accessible mesoporous network and high surface area (MAC-270-2-7: 703 $m^2$ $g^{-1}$) of the material is three times larger than that of its corresponding control (MAC-270-0-7: 236 $m^2$ $g^{-1}$; Table S2 and Figure S7).

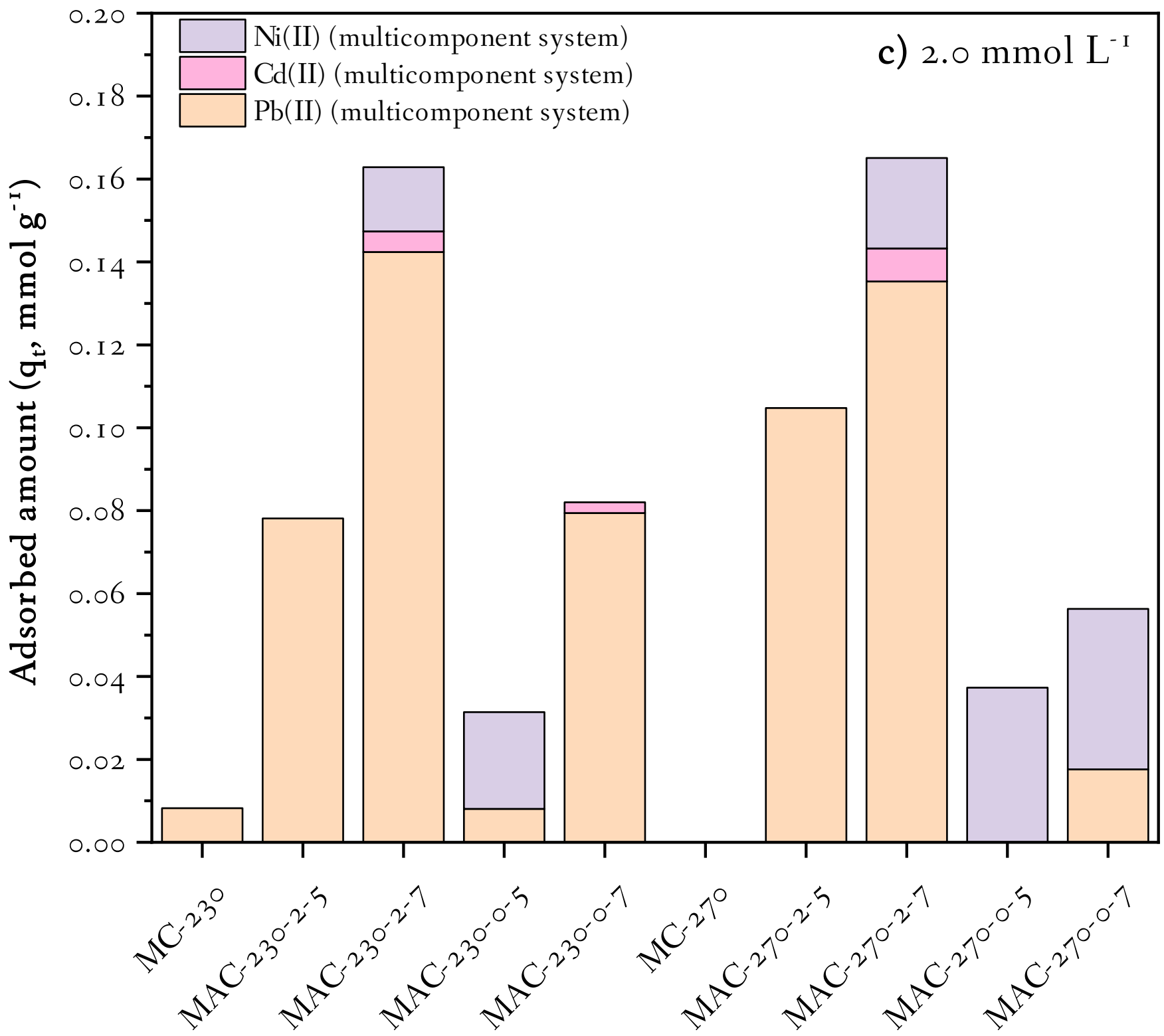

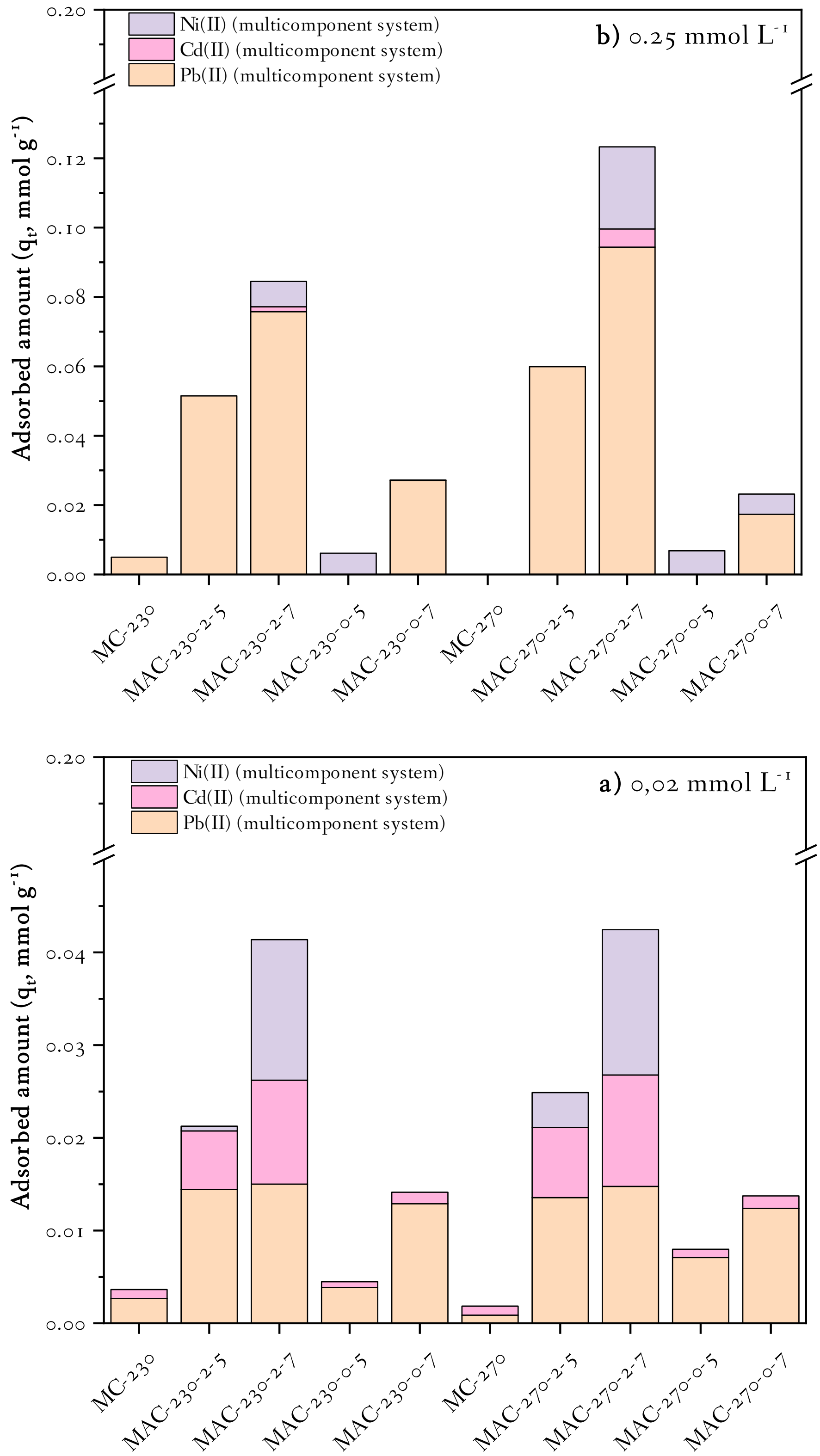


**Figure S6.** Comparison of the adsorbed amounts of Pb(II), Cd(II), and Ni(II) in multicomponent solutions at concentrations of a) 0.02 mmol $L^{-1}$, b) 0.25 mmol $L^{-1}$, and c) 2.0 mmol $L^{-1}$ for each metal. Contact time: 24 h, adsorbent dose: 1.00 g $L^{-1}$, initial pH: 4.5, and temperature: 21.0 ± 0.4 °C.

$N_2$ adsorption-desorption isotherms of the MAC-C are shown in Figure S7 and are classified as type IV(a) according to the International Union of Pure and Applied Chemistry (IUPAC),[11] characteristic of mesoporous solids. A similar behavior was observed for the MACs.[1] The hysteresis of MAC-C at 500 °C was of type H3, resembling that of MAC. In contrast, the MAC-C at 700 °C exhibited H4-type hysteresis, which is similar to H3 but with the adsorption branch combining features of type I and II, indicating stronger adsorption at low relative pressures associated with micropore filling. H4 hysteresis is commonly observed in aggregated zeolite crystals, certain mesoporous zeolites, and micro-mesoporous carbons.[11]

MAC-C at 500 °C exhibited specific surface areas 3.4 and 1.7 times higher than their KOH-activated counterparts.[1] On the other hand, at 700 °C, the controls displayed surface areas 1.2 and 3.0 times lower than those of KOH-activated materials.[1] This behavior is attributed to thermal degradation and volatilization processes during activation, which generate porosity even in the absence of chemical agents, but are prone to collapse at higher temperatures.[9,10] The pores formed by thermal activation are predominantly microporous (Table S2), as also reported in previous studies,[9,10] which are often less accessible or interconnected, potentially limiting adsorption of larger molecules or at higher concentrations. These results highlight the critical role of KOH thermochemical activation in enhancing the textural properties of MAC.

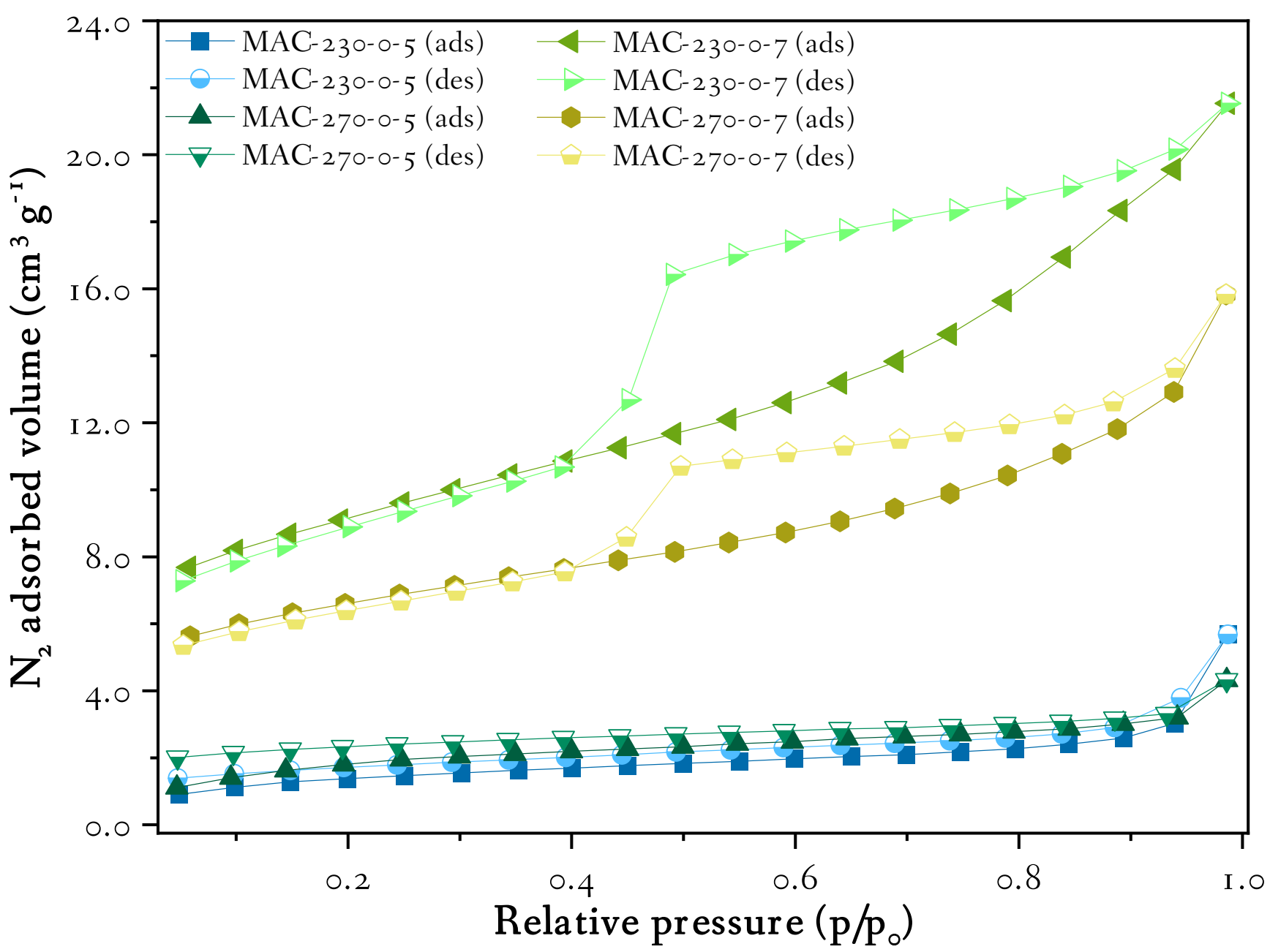


**Figure S7.** $N_2$ adsorption-desorption isotherms of thermally activated control materials without KOH: MAC-230-0-5, MAC-270-0-5, MAC-230-0-7, and MAC-270-0-7.

Finally, single-component experiments at 0.25 mmol $L^{-1}$ (Figure S8) confirmed higher individual adsorption capacities than in multicomponent systems, clearly reflecting the competitive antagonistic effects among the ions. In single-component matrices, MAC-270-2-7 demonstrated its highest individual uptakes (0.14, 0.07, and 0.12 mmol $g^{-1}$ for Pb(II), Cd(II), and Ni(II), respectively). Additionally, the sum of the adsorbed moles in the multicomponent system is lower than the sum of the individual capacities in the single-component system. This finding indicates that the metals actively compete for the same available surface-active sites. Its robust performance across both systems supports its selection as the optimal material for the subsequent studies.

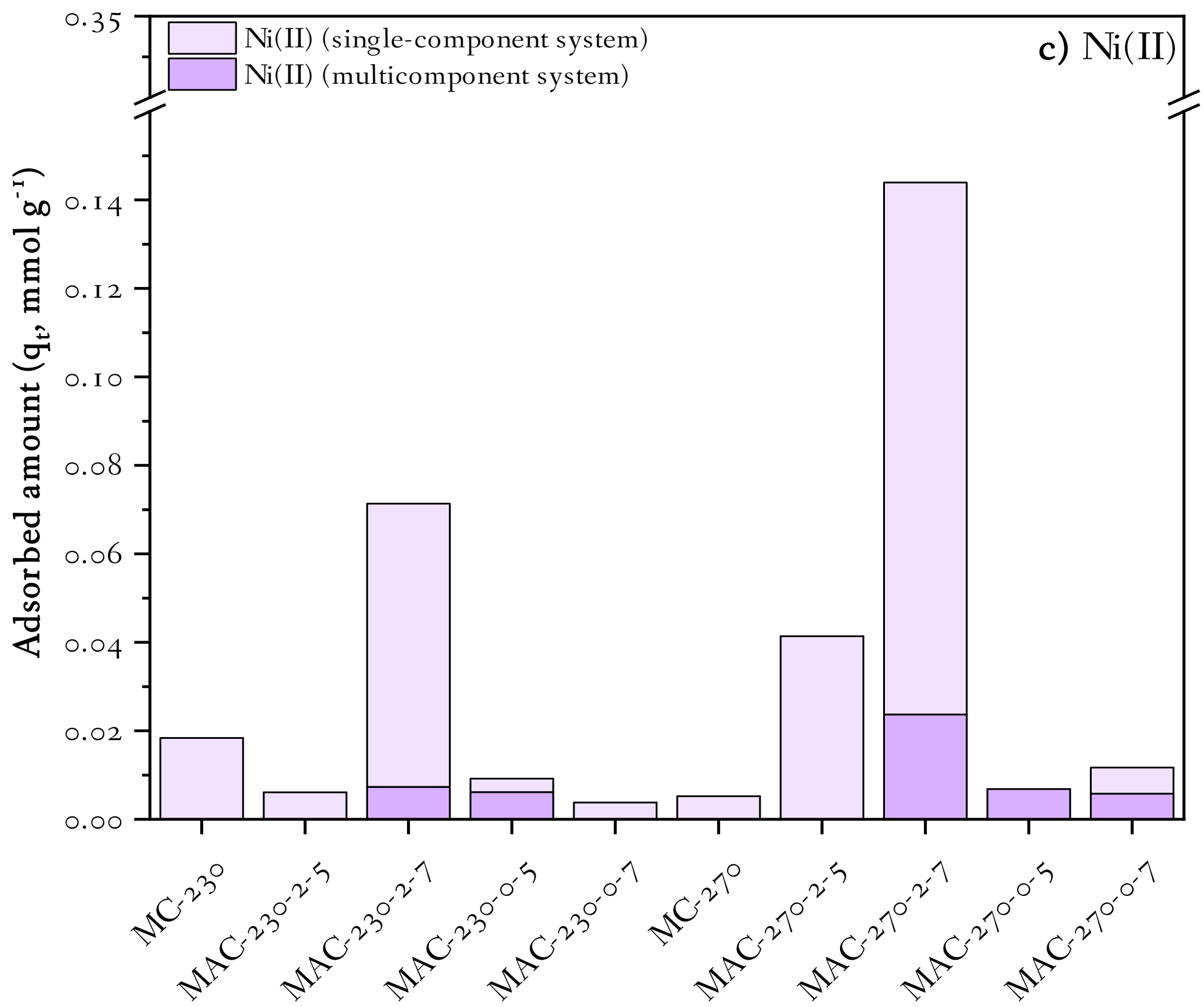

Ni(II) (single-component system)
Ni(II) (multicomponent system)
c) Ni(II)
Adsorbed amount ($q_t$, mmol g$^{-1}$)
0.35
0.14
0.12
0.10
0.08
0.06
0.04
0.02
0.00
MC-230
MAC-230-2-5
MAC-230-2-7
MAC-230-0-5
MAC-230-0-7
MC-270
MAC-270-2-5
MAC-270-2-7
MAC-270-0-5
MAC-270-0-7


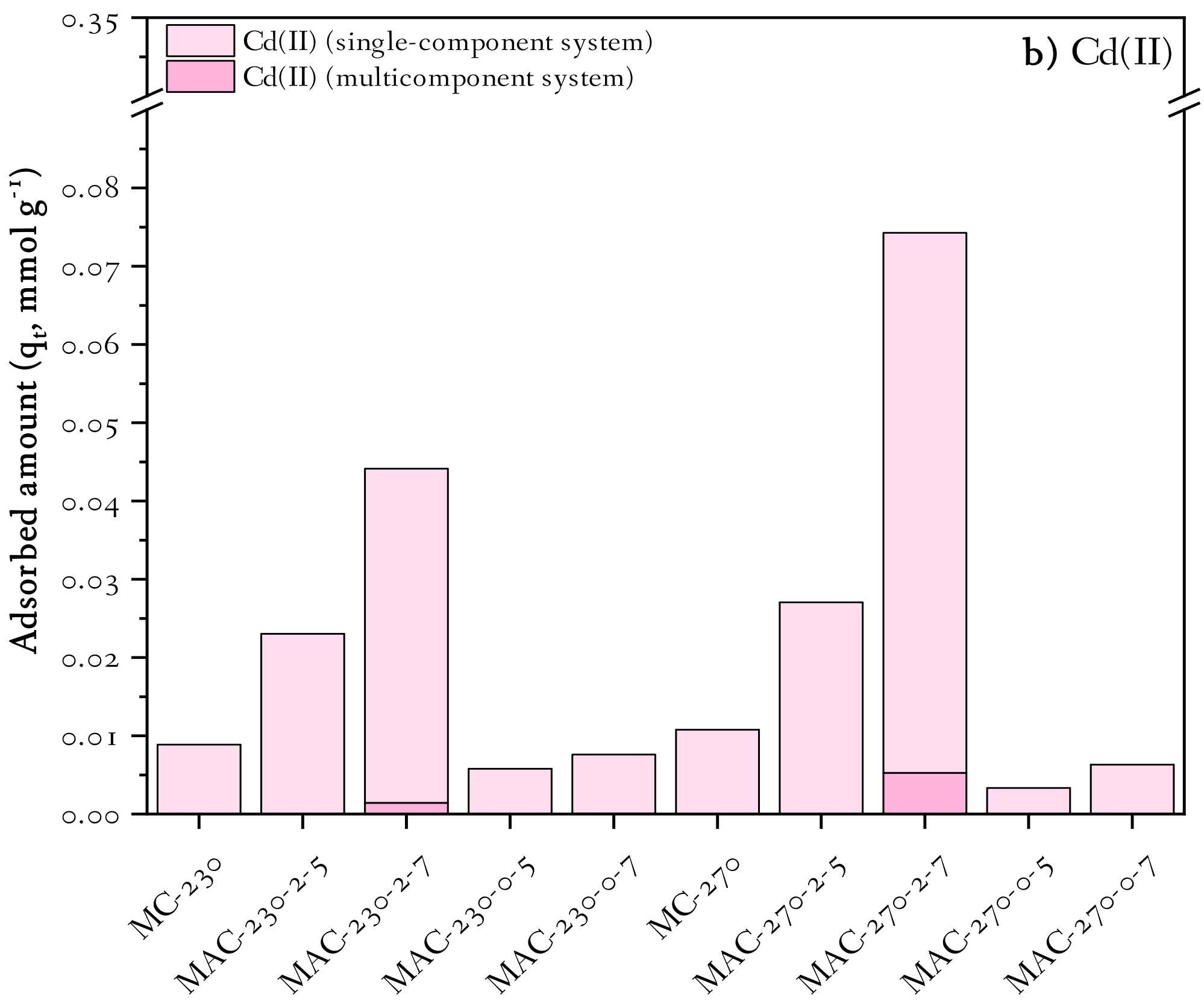

Cd(II) (single-component system)
Cd(II) (multicomponent system)
b) Cd(II)
Adsorbed amount ($q_t$, mmol g$^{-1}$)
0.35
0.08
0.07
0.06
0.05
0.04
0.03
0.02
0.01
0.00
MC-230
MAC-230-2-5
MAC-230-2-7
MAC-230-0-5
MAC-230-0-7
MC-270
MAC-270-2-5
MAC-270-2-7
MAC-270-0-5
MAC-270-0-7

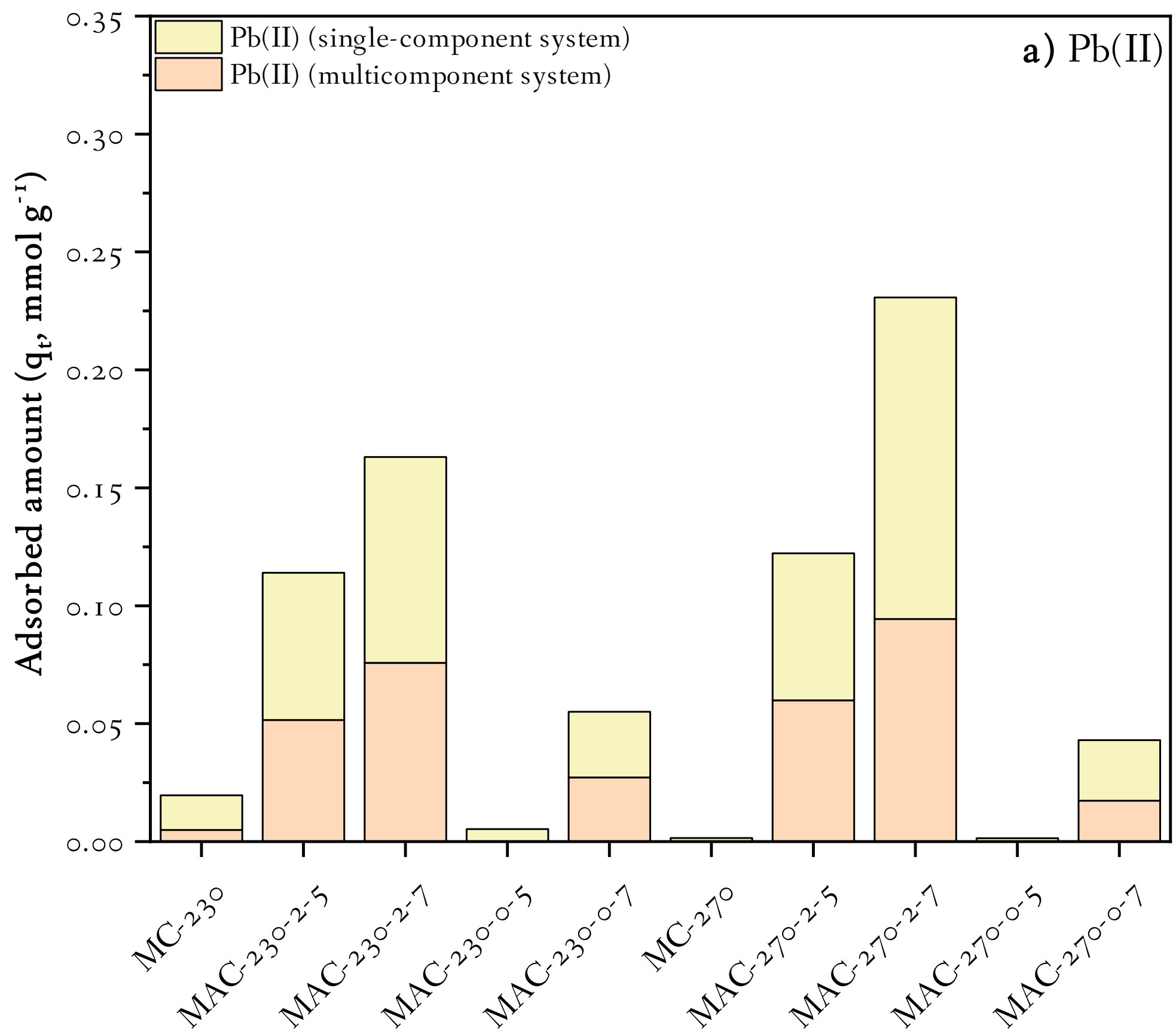


**Figure S8**. Comparison of the adsorbed amounts of a) Pb(II), b) Cd(II), and c) Ni(II) in single and multicomponent solutions at a concentration of 0.25 mmol $L^{-1}$ for each metal. Contact time: 24 h, MAC-270-2-7 dose: 1.00 g $L^{-1}$, initial pH: 4.5, and temperature: 21.0 ± 0.4 °C.

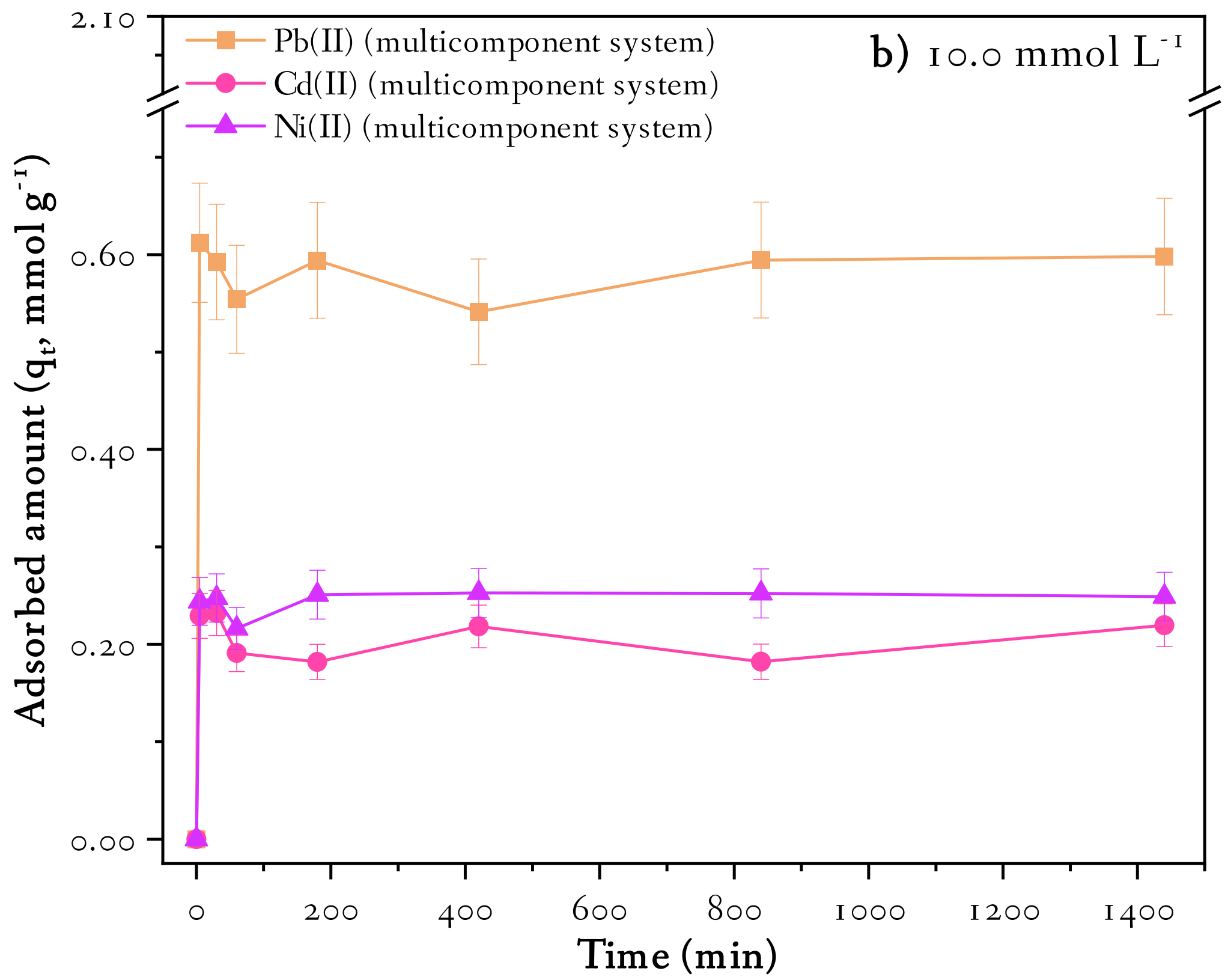

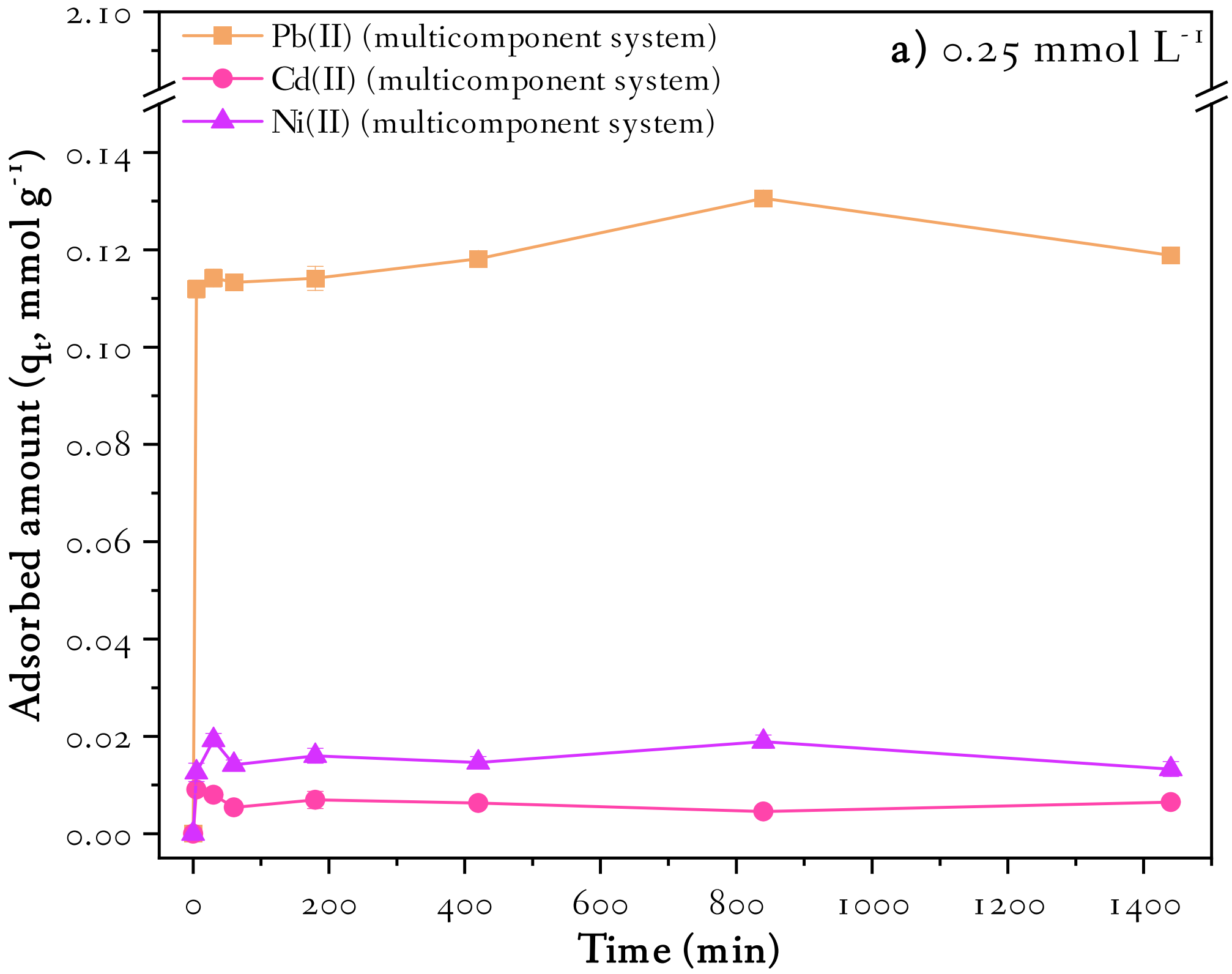


**Figure S9.** Effect of contact time in the multicomponent adsorption of Pb(II), Cd(II), and Ni(II) (5-1440 min). Initial pH: 5.0, MAC-270-2-7 dose: 0.50 g $L^{-1}$, concentration of multicomponent solutions: a) 0.25 mmol $L^{-1}$ and b) 10.0 mmol $L^{-1}$ for each metal. Temperature: 21.0 ± 0.4 °C.

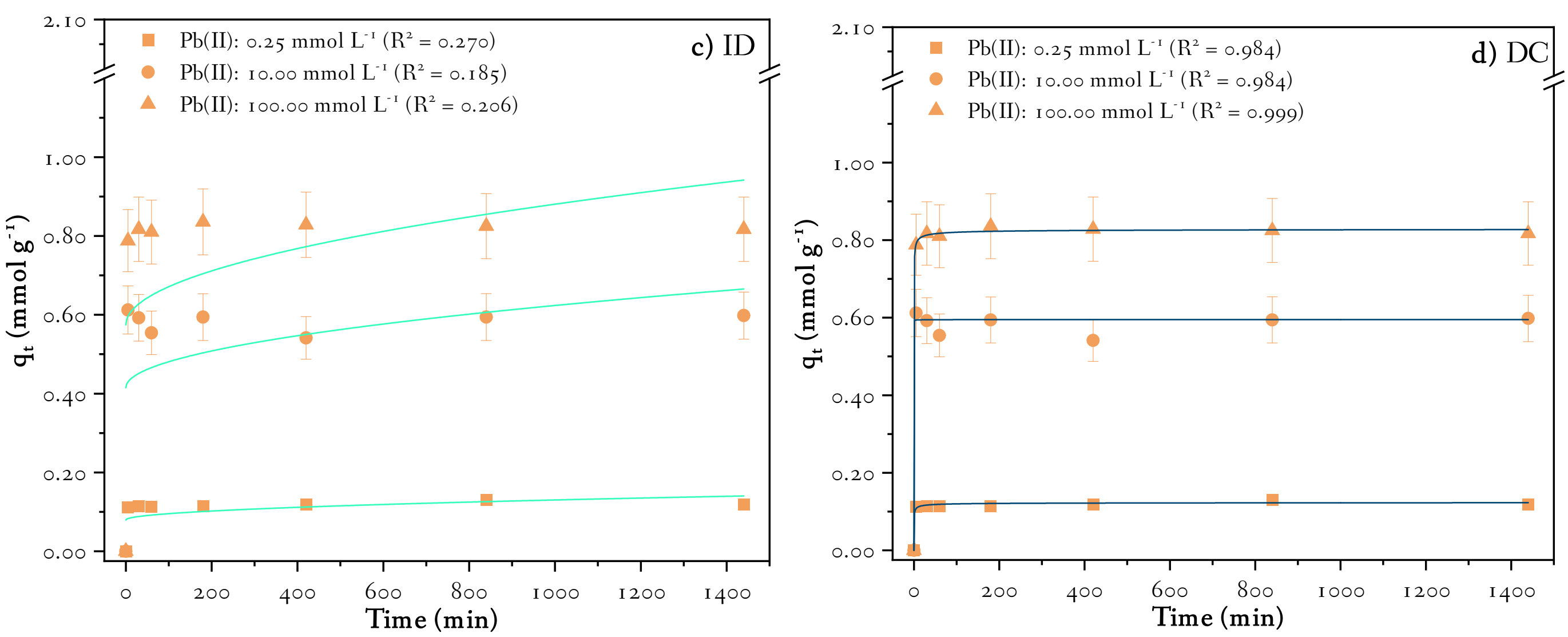

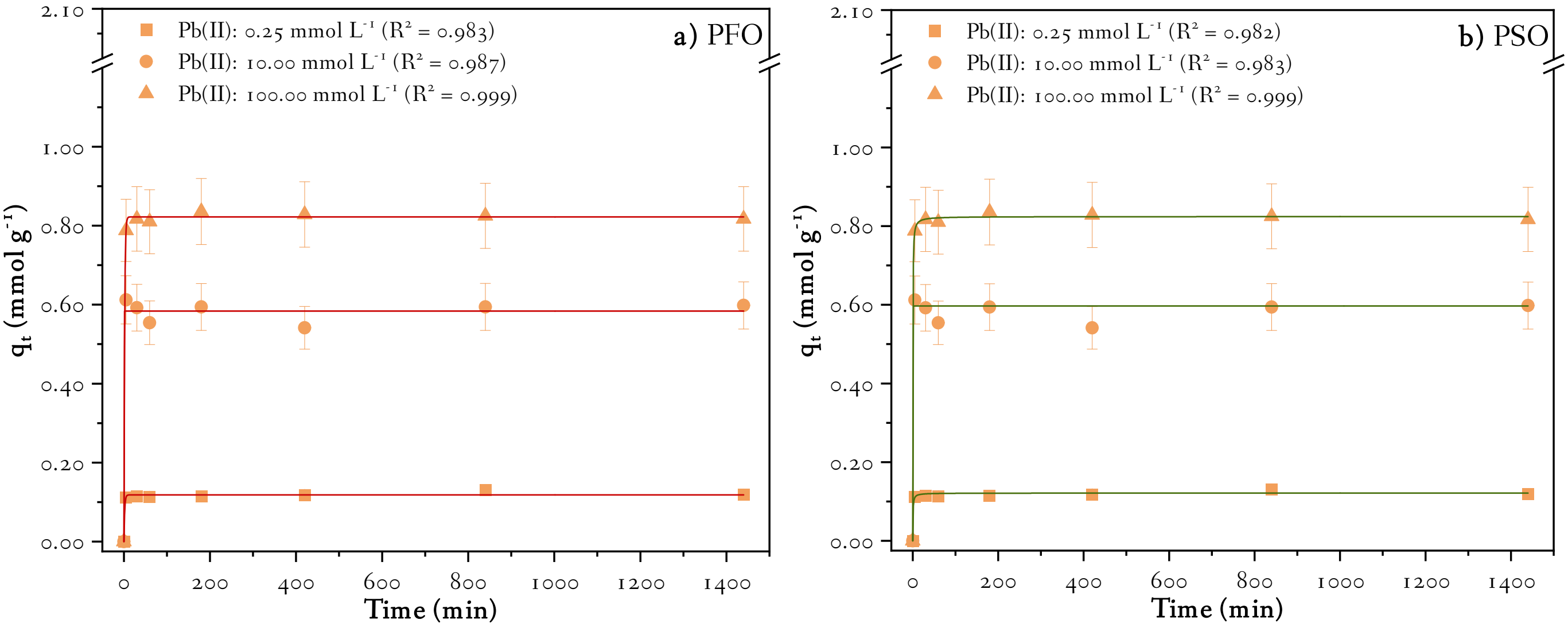


**Figure S10.** Nonlinear adjustments to the kinetic models of a) PFO, b) PSO, c) ID, and d) DC for MAC-270-2-7 in the multicomponent adsorption of Pb(II).

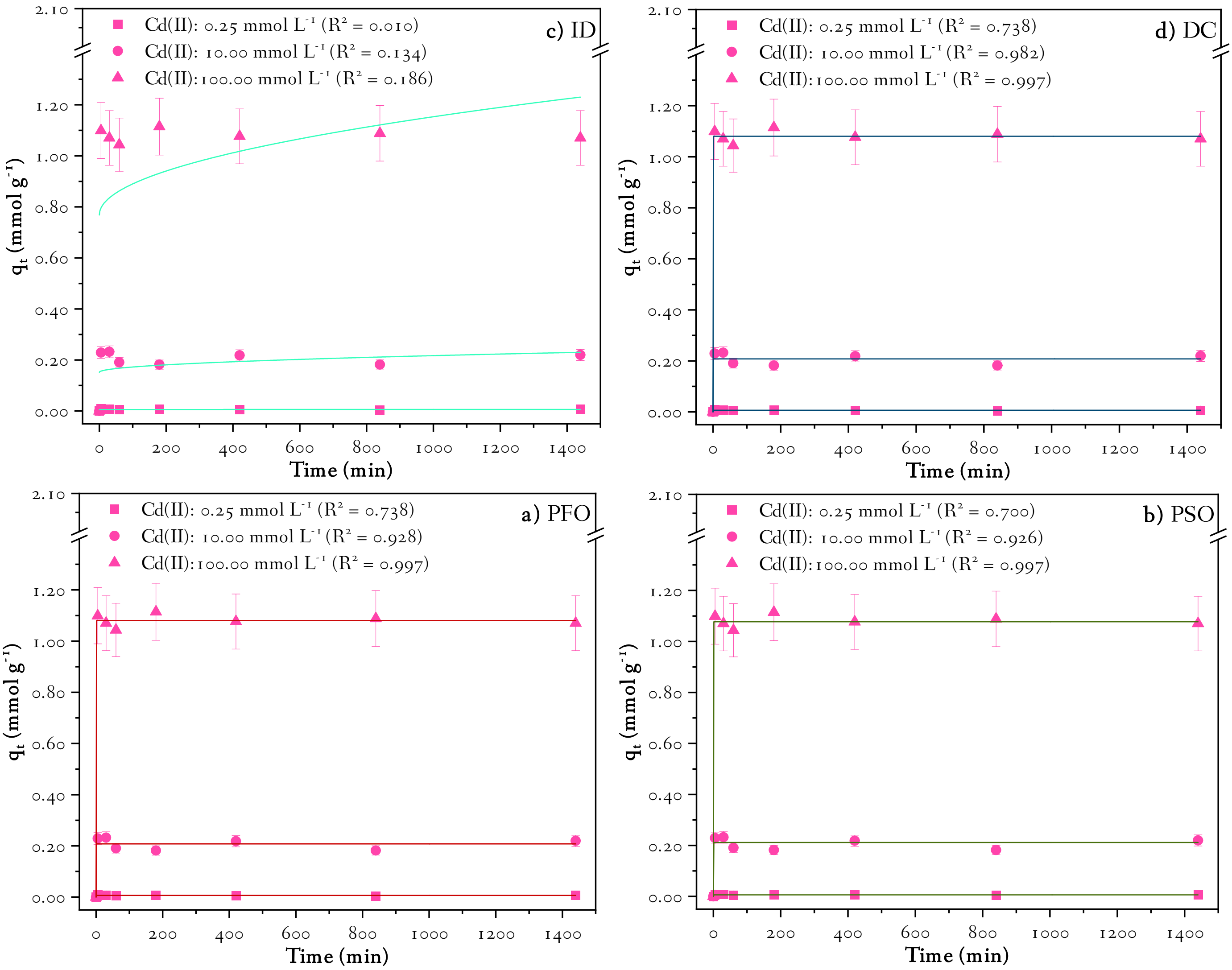

**Figure S11.** Nonlinear adjustments to the kinetic models of a) PFO, b) PSO, c) ID, and d) DC for MAC-270-2-7 in the multicomponent adsorption of Cd(II).

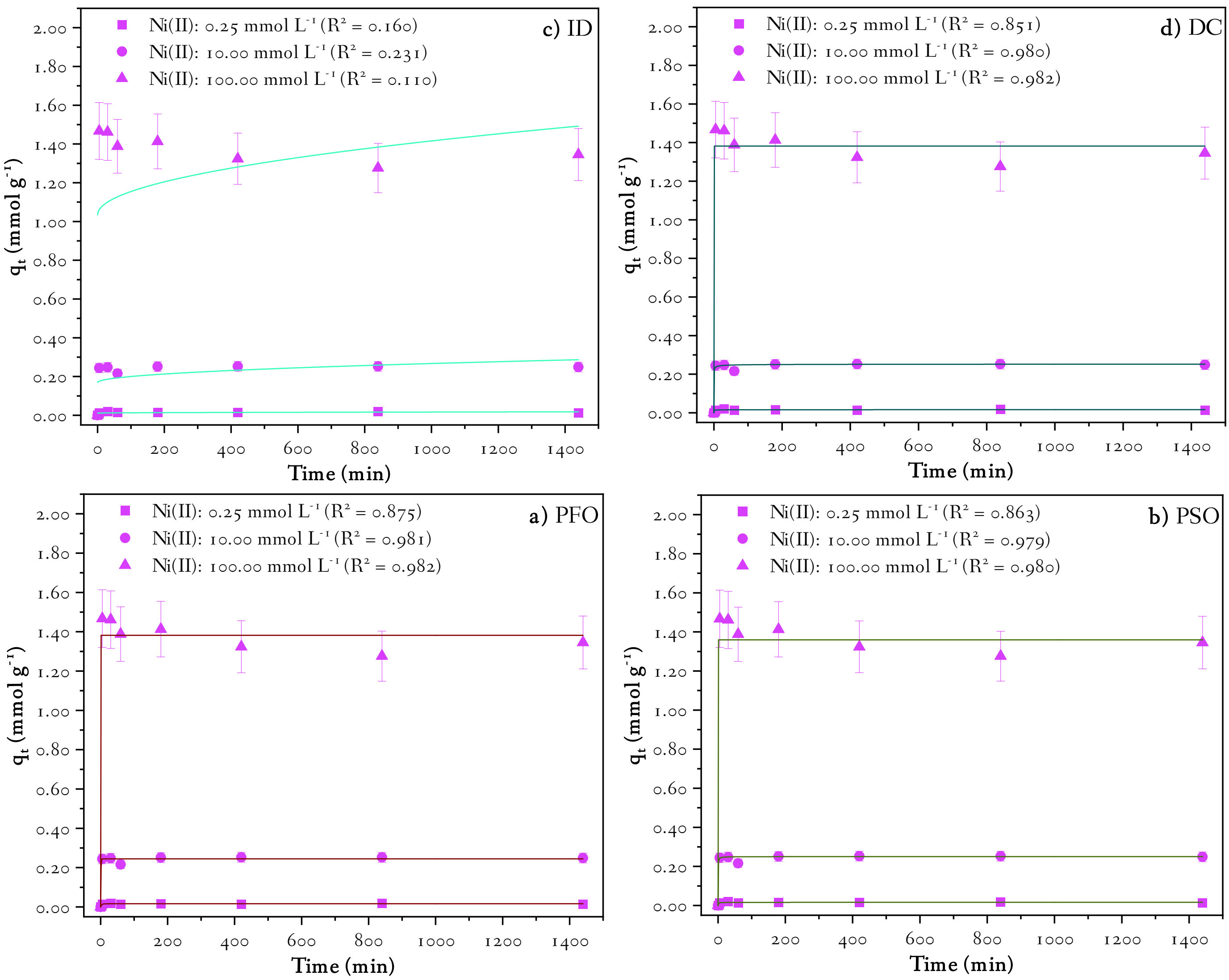


**Figure S12.** Nonlinear adjustments to the kinetic models of a) PFO, b) PSO, c) ID, and d) DC for MAC-270-2-7 in the multicomponent adsorption of Ni(II).

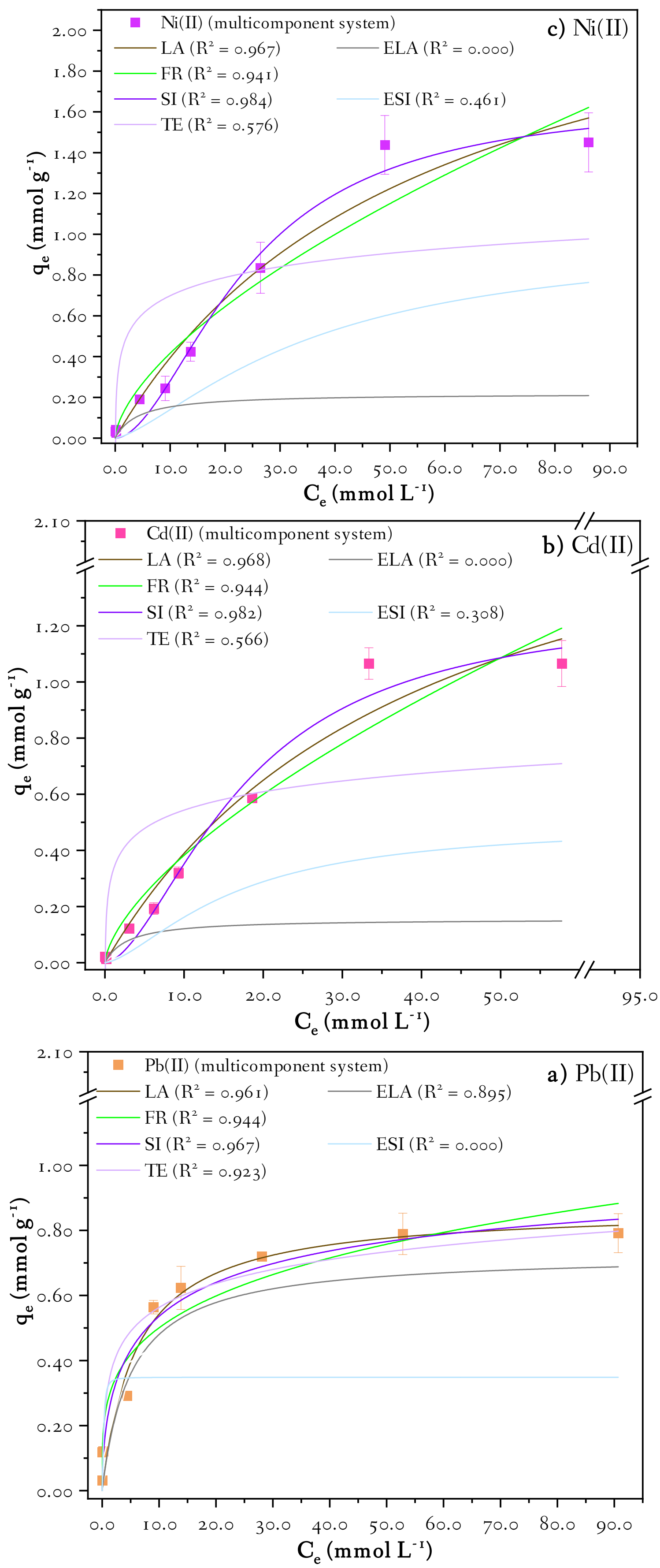


**Figure S13.** Nonlinear adjustments to the isothermal models of LA, FR, SI, TE, ELA, and ESI for MAC-270-2-7 in the multicomponent adsorption of a) Pb(II), b) Cd(II), and c) Ni(II).

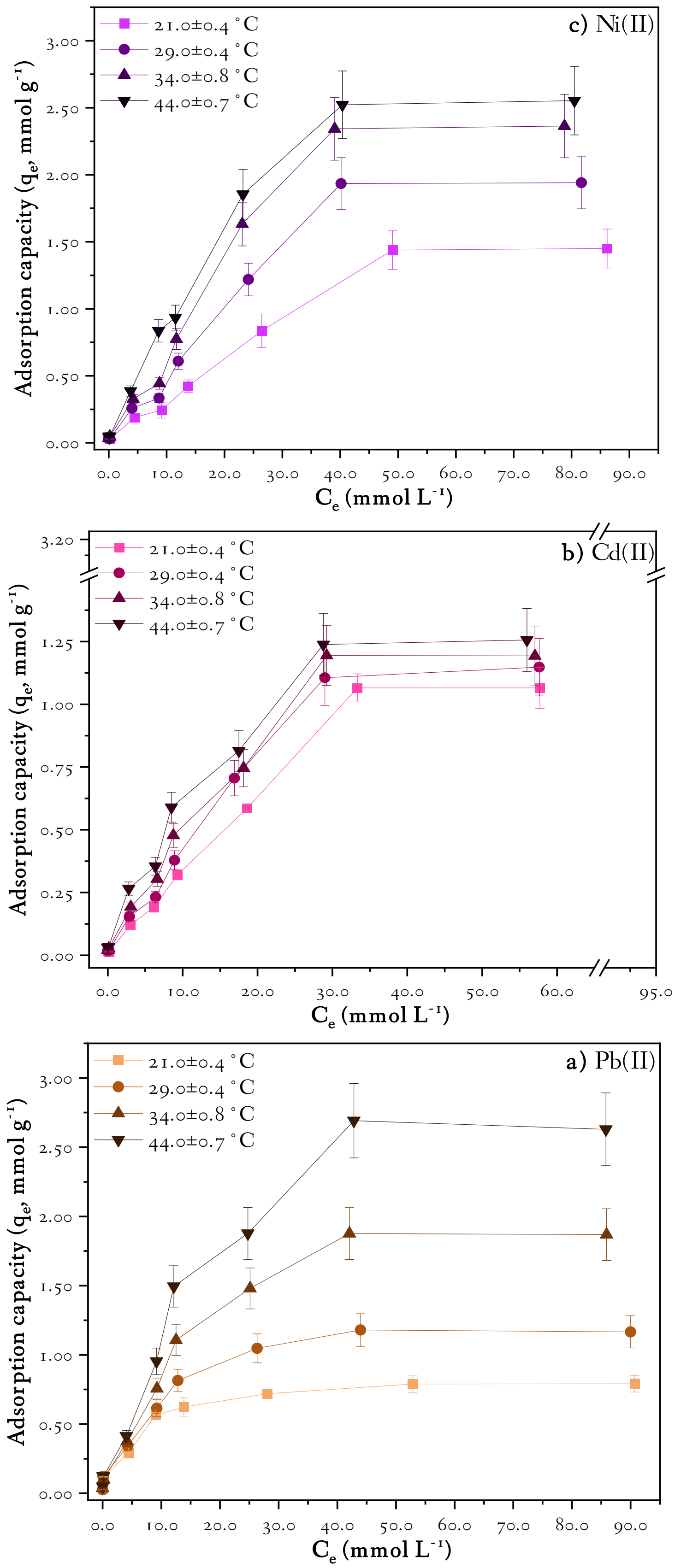


**Figure S14.** Multicomponent adsorption isotherms as a function of temperature for a) Pb(II), b) Cd(II), and c) Ni(II). Dose of MAC-270-2-7: 0.50 g L$^{-1}$, initial pH: 5.0, contact time: 30 minutes, and concentration: 0.02-100.0 mmol L$^{-1}$ for each metal.

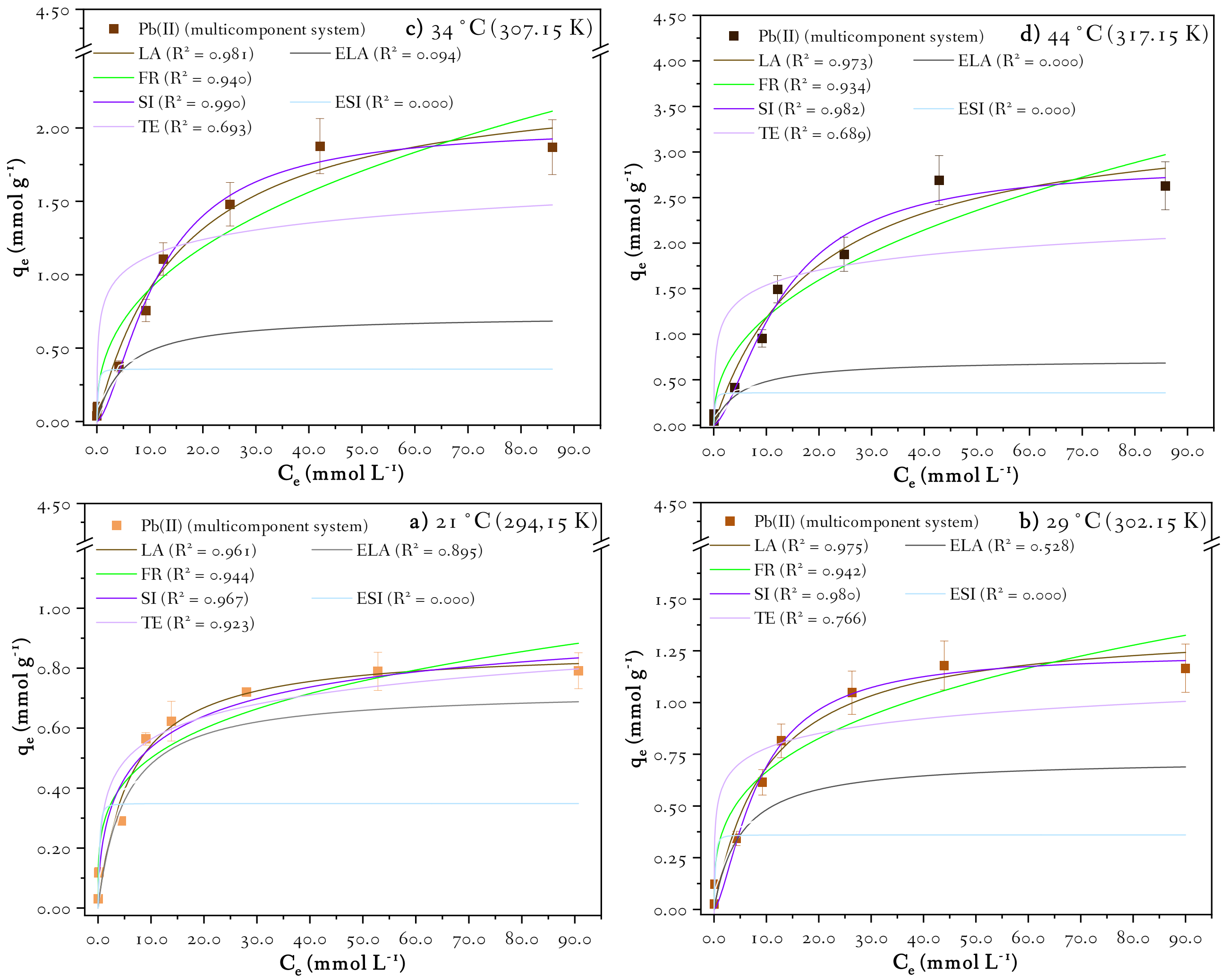


**Figure S15.** Nonlinear fits to the LA, FR, SI, TE, ELA, and ESI isothermal models for MAC-270-2-7 in the multicomponent adsorption of Pb(II) at temperatures of a) 21 °C, b) 29 °C, c) 34 °C, and d) 44 °C.

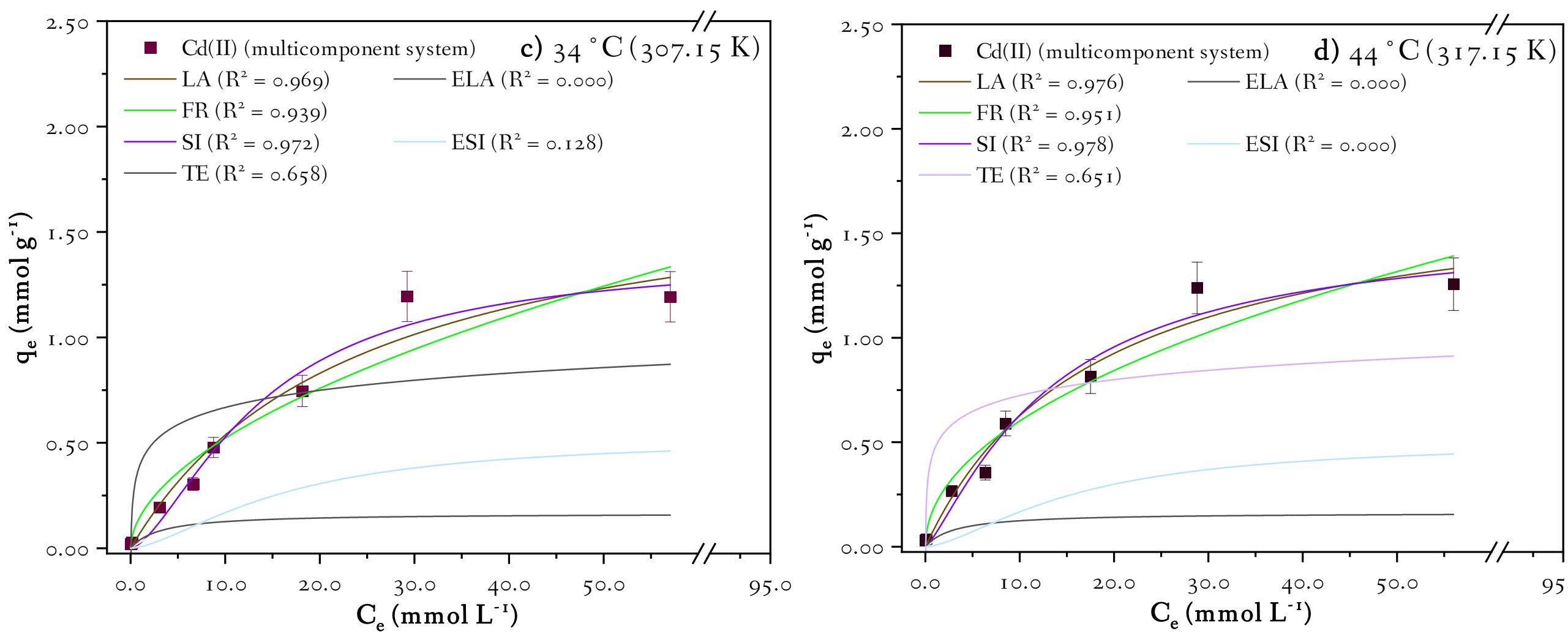

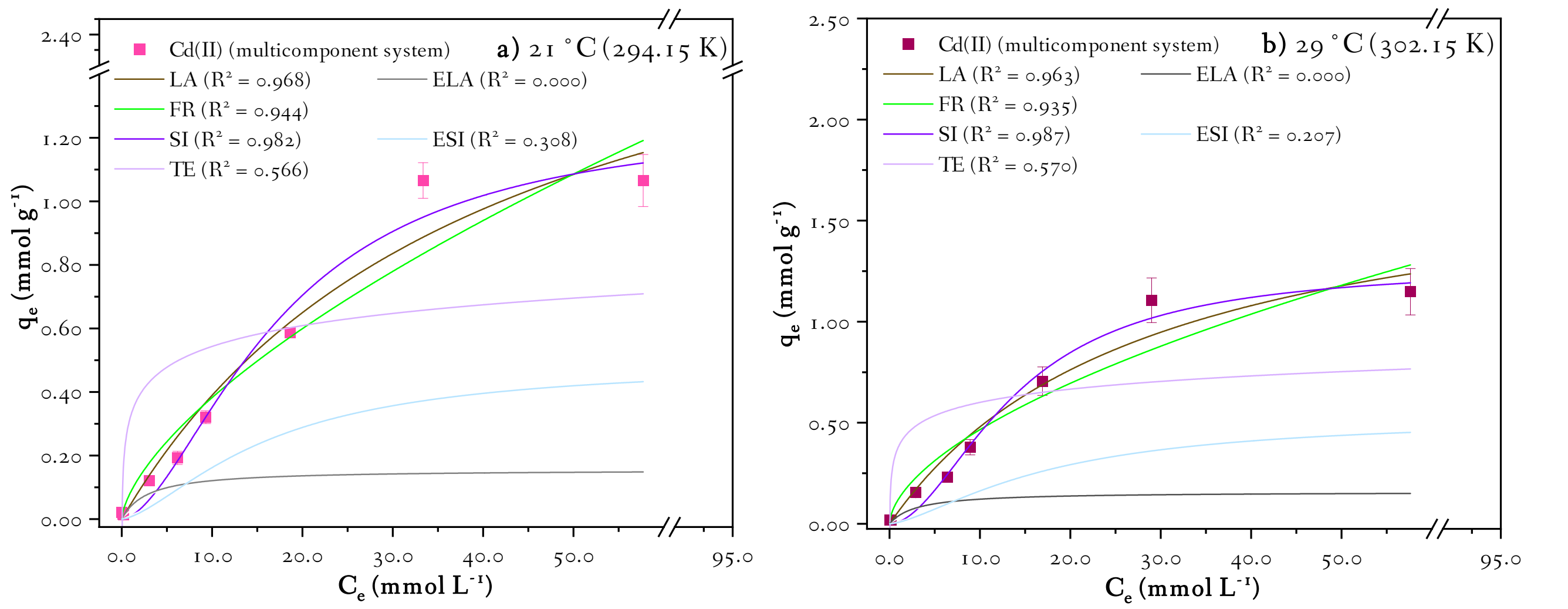


**Figure S16.** Nonlinear fits to the LA, FR, SI, TE, ELA, and ESI isothermal models for MAC-270-2-7 in the multicomponent adsorption of Cd(II) at temperatures of a) 21 °C, b) 29 °C, c) 34 °C, and d) 44 °C.

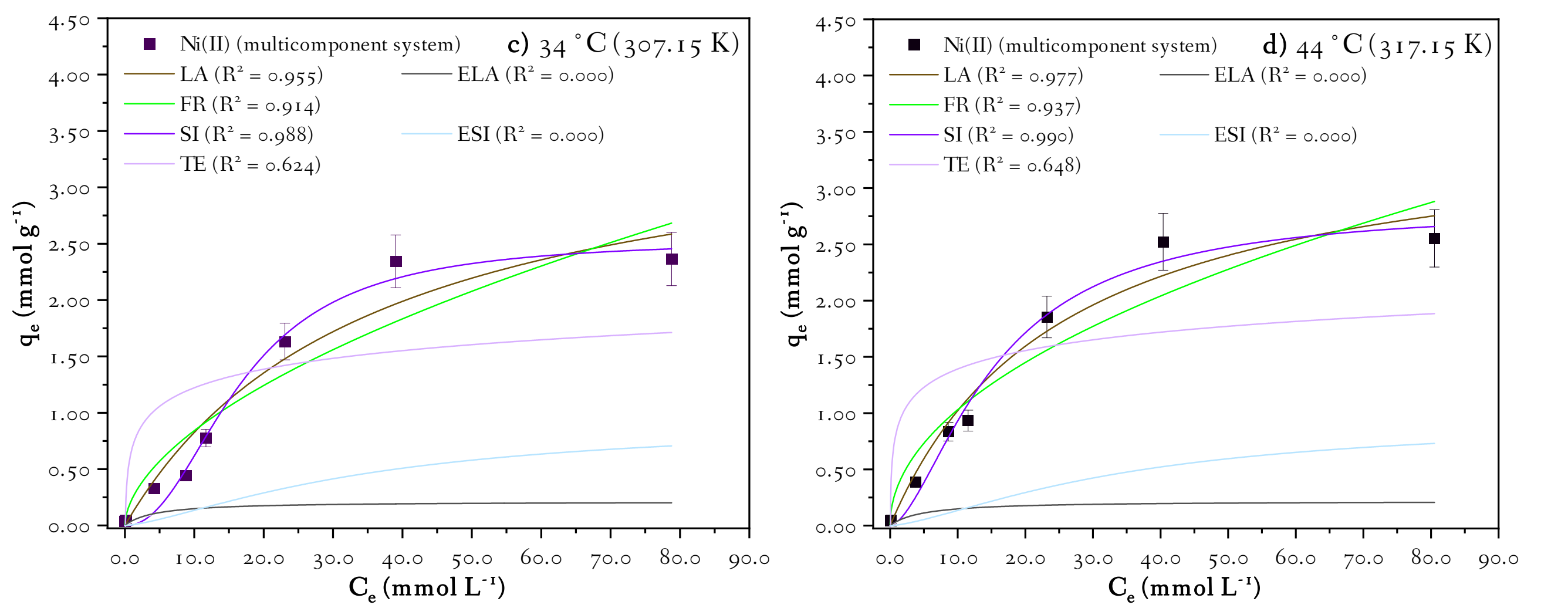


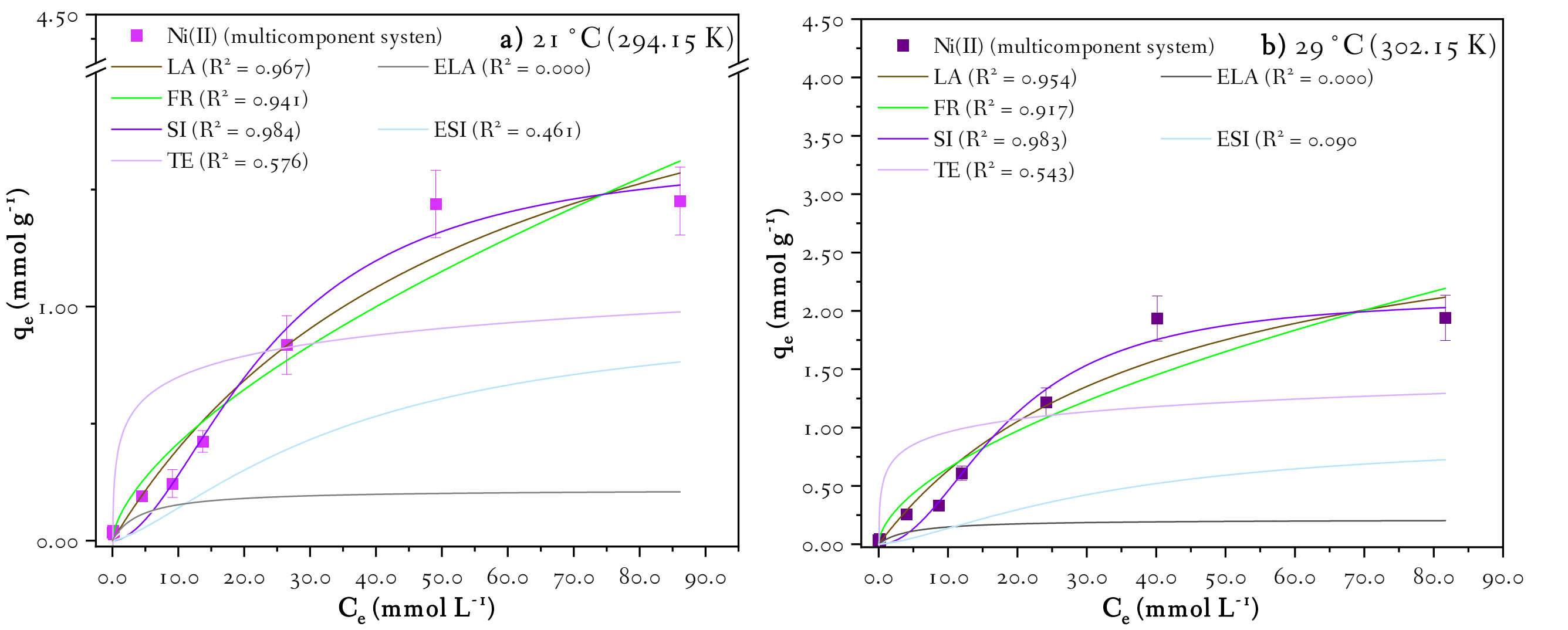

**Figure S17.** Nonlinear fits to the LA, FR, SI, TE, ELA, and ESI isothermal models for MAC-270-2-7 in the multicomponent adsorption of Ni(II) at temperatures of a) 21 °C, b) 29 °C, c) 34 °C, and d) 44 °C.

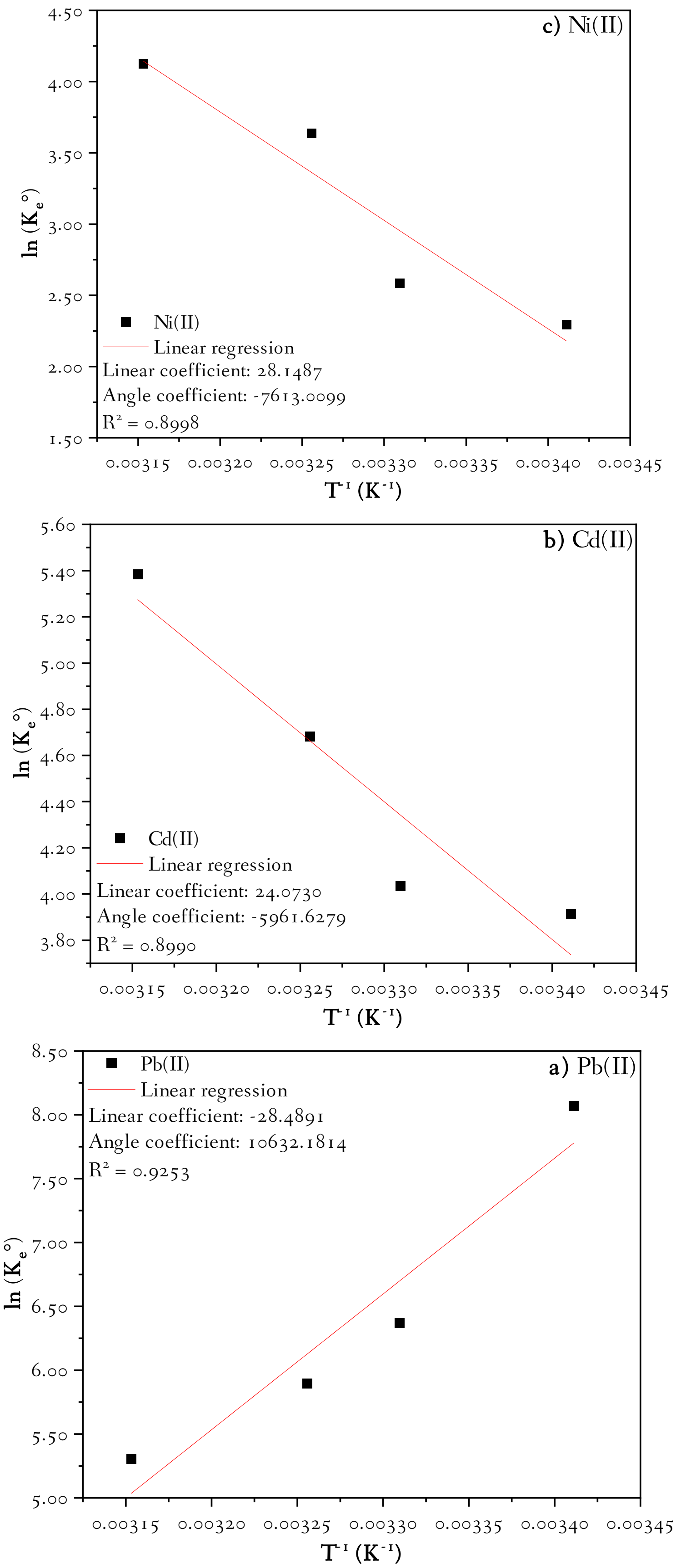


**Figure S18.** Linear adjustments of ln $K_e°$ versus $T^{-1}$ to obtain the thermodynamic parameters of adsorption of a) Pb(II), b) Cd(II), and c) Ni(II).

## References


(1) Nogueira, G. A.; Fregolente, L. G.; Pereira, L. S.; Laranja, M. J.; Moreira, A. B.; Ferreira, O. P.; Bisinoti, M. C. Magnetic Activated Carbonaceous Materials from Sugarcane Bagasse: Preparation, Characterization, and Hexavalent Chromium Removal. *Materials Today Sustainability* **2024**, *28*, 101040. https://doi.org/10.1016/j.mtsust.2024.101040

(2) Lima, E. C.; Hosseini-Bandegharaei, A.; Moreno-Piraján, J. C.; Anastopoulos, I. A Critical Review of the Estimation of the Thermodynamic Parameters on Adsorption Equilibria. Wrong Use of Equilibrium Constant in the Van't Hoof Equation for Calculation of Thermodynamic Parameters of Adsorption. *J. Mol. Liq.* **2019**, *273*, 425–434. https://doi.org/10.1016/J.MOLLIQ.2018.10.048

(3) Qu, J.; Tian, X.; Jiang, Z.; Cao, B.; Akindolie, M. S.; Hu, Q.; Feng, C.; Feng, Y.; Meng, X.; Zhang, Y. Multi-Component Adsorption of Pb(II), Cd(II) and Ni(II) onto Microwave-Functionalized Cellulose: Kinetics, Isotherms, Thermodynamics, Mechanisms and Application for Electroplating Wastewater Purification. *J. Hazard. Mater.* **2020**, *387*, 121718. https://doi.org/10.1016/J.JHAZMAT.2019.121718

(4) Haynes, W. M.; Lide, D. R.; Bruno, T. J. *CRC Handbook of Chemistry and Physics 97 Th Edition*; 2016.

(5) Kontogeorgis, G. M.; Maribo-Mogensen, B.; Thomsen, K. The Debye-Hückel Theory and Its Importance in Modeling Electrolyte Solutions. *Fluid Phase Equilib.* **2018**, *462*, 130–152. https://doi.org/10.1016/J.FLUID.2018.01.004

(6) Sun, L.; Lei, Q.; Peng, B.; Kontogeorgis, G. M.; Liang, X. An Analysis of the Parameters in the Debye-Hückel Theory. *Fluid Phase Equilib.* **2022**, *556*, 113398. https://doi.org/10.1016/J.FLUID.2022.113398

(7) Arcibar-Orozco, J. A.; Rangel-Mendez, J. R.; Diaz-Flores, P. E. Simultaneous Adsorption of Pb(Ii)-Cd(Ii), Pb(Ii)-Phenol, and Cd(Ii)-Phenol by Activated Carbon Cloth in Aqueous Solution. *Water Air Soil Pollut.* **2015**, *226* (1). https://doi.org/10.1007/s11270-014-2197-1

(8) Kong, Z.; Zhang, H.; Zhou, T.; Xie, L.; Wang, B.; Jiang, X. Biomass-Derived Functional Materials: Preparation, Functionalization, and Applications in Adsorption and Catalytic Separation of Carbon Dioxide and Other Atmospheric Pollutants. *Sep. Purif. Technol.* **2025**, *354*, 129099. https://doi.org/10.1016/J.SEPPUR.2024.129099

(9) Dobruskin, V. K. Contribution of the Edge Effect to Physical Adsorption in Micropores of Activated Carbons. *Carbon N. Y.* **2002**, *40* (5), 659–666. https://doi.org/10.1016/S0008-6223(01)00181-6

(10) Vilaplana-Ortego, E.; Maciá-Agulló, J. A.; Alcañiz-Monge, J.; Cazorla-Amorós, D.; Linares-Solano, A. Comparative Study of the Micropore Development on Physical Activation of Carbon Fibers from Coal Tar and Petroleum Pitches. *Microporous and Mesoporous Materials* **2008**, *112* (1–3), 125–132. https://doi.org/10.1016/J.MICROMESO.2007.09.019

(11) Thommes, M.; Kaneko, K.; Neimark, A. V.; Olivier, J. P.; Rodriguez-Reinoso, F.; Rouquerol, J.; Sing, K. S. W. Physisorption of Gases, with Special Reference to the Evaluation of Surface Area and Pore Size Distribution (IUPAC Technical Report). *Pure and Applied Chemistry* **2015**, *87* (9–10), 1051–1069. https://doi.org/10.1515/pac-2014-1117
